# Integration of targeted metabolomics and transcriptomics identifies deregulation of phosphatidylcholine metabolism in Huntington’s disease peripheral blood samples

**DOI:** 10.1007/s11306-016-1084-8

**Published:** 2016-07-27

**Authors:** Anastasios Mastrokolias, Rene Pool, Eleni Mina, Kristina M. Hettne, Erik van Duijn, Roos C. van der Mast, GertJan van Ommen, Peter A. C. ‘t Hoen, Cornelia Prehn, Jerzy Adamski, Willeke van Roon-Mom

**Affiliations:** 1 Department of Human Genetics, Leiden University Medical Center, 2300 RC Leiden, The Netherlands; 2 Department of Biological Psychology, Faculty of Psychology and Education, VU University Amsterdam, Amsterdam, The Netherlands; 3 The EMGO + Institute for Health and Care Research, VU University Medical Center, Amsterdam, The Netherlands; 4 Department of Psychiatry, Leiden University Medical Center, 2300 RC Leiden, The Netherlands; 5 Center for Mental Health Care Delfland, Jorisweg 2, Delft, The Netherlands; 6 Helmholtz Zentrum, München, German Research Center for Environmental Health, Institute of Experimental Genetics, Genome Analysis Center, Neuherberg, Germany; 7 German Center for Diabetes Research, Neuherberg, Germany; 8 Lehrstuhl für Experimentelle Genetik, Technische Universität München, Freising-Weihenstephan, Germany

**Keywords:** Metabolomics, Gene expression, Biomarkers, Disease progression, Neurodegenerative, Integrated analysis

## Abstract

**Introduction:**

Metabolic changes have been frequently associated with Huntington’s disease (HD). At the same time peripheral blood represents a minimally invasive sampling avenue with little distress to Huntington’s disease patients especially when brain or other tissue samples are difficult to collect.

**Objectives:**

We investigated the levels of 163 metabolites in HD patient and control serum samples in order to identify disease related changes. Additionally, we integrated the metabolomics data with our previously published next generation sequencing-based gene expression data from the same patients in order to interconnect the metabolomics changes with transcriptional alterations.

**Methods:**

This analysis was performed using targeted metabolomics and flow injection electrospray ionization tandem mass spectrometry in 133 serum samples from 97 Huntington’s disease patients (29 pre-symptomatic and 68 symptomatic) and 36 controls.

**Results:**

By comparing HD mutation carriers with controls we identified 3 metabolites significantly changed in HD (serine and threonine and one phosphatidylcholine—PC ae C36:0) and an additional 8 phosphatidylcholines (PC aa C38:6, PC aa C36:0, PC ae C38:0, PC aa C38:0, PC ae C38:6, PC ae C42:0, PC aa C36:5 and PC ae C36:0) that exhibited a significant association with disease severity. Using workflow based exploitation of pathway databases and by integrating our metabolomics data with our gene expression data from the same patients we identified 4 deregulated phosphatidylcholine metabolism related genes (*ALDH1B1*, *MBOAT1*, *MTRR* and *PLB1*) that showed significant association with the changes in metabolite concentrations.

**Conclusion:**

Our results support the notion that phosphatidylcholine metabolism is deregulated in HD blood and that these metabolite alterations are associated with specific gene expression changes.

**Electronic supplementary material:**

The online version of this article (doi:10.1007/s11306-016-1084-8) contains supplementary material, which is available to authorized users.

## Introduction

Huntington’s disease (HD) is an autosomal dominant neurodegenerative disorder that presents itself through motor dysfunction, psychiatric disturbances and cognitive decline. The pathology is caused by an expanded CAG repeat in the *HTT* gene, resulting in a mutant huntingtin protein (The Huntington’s Disease Collaborative Research Group 1993). A characteristic of HD is mutant protein aggregate formation and neuronal cell loss in the brain but it is also known that HD patients develop peripheral tissue symptoms such as muscle atrophy, impaired glucose tolerance and weight loss (Lalic et al. 2008; Zielonka et al. 2014). The mutation for HD was discovered more than 20 years ago and much is known about the underlying disease mechanisms (Ross et al. 2014). Moreover, recent studies show that lowering mutant huntingtin protein levels using RNAi is a promising therapeutic approach that is close to clinical trials (Yu et al. 2012; Evers et al. 2011). This highlights/prompts the need for biomarkers that track disease progression and measure clinical trial therapeutic effectiveness.

Deregulation of energy and metabolic pathways have been repeatedly implicated in HD (Acuna et al. 2013; Mochel and Haller 2011; Tang et al. 2013; Johri et al. 2013). Specifically, defects in lipid homeostasis have been proposed as contributors to disease onset (Gulati et al. 2010; Valenza and Cattaneo 2011; Sipione et al. 2002). Additionally, total cholesterol was found to be significantly reduced even outside the brain when human fibroblasts were cultured in lipoprotein-deprived serum (Valenza et al. 2005). Previous studies using HD transgenic models and human caudate samples have shown a deregulation of genes involved in glycosphingolipid metabolism, selected brain gangliosides as well as neutral and acidic lipids. Additionally, Wang and colleagues were able to discover metabolic hormonal plasma signatures in presymptomatic and symptomatic HD patients suggesting that in HD metabolic hormone secretion and energy regulation is affected (Wang et al. 2014). Previous mass spectrometry studies have shown differences in the serum metabolome of transgenic HD mice and wild type controls with a similar trend in human samples implicating changes in fatty acid breakdown and certain aliphatic amino acids (Underwood et al. 2006). Consequently such approaches that use mass spectrometry metabolomics on brain as well as non-nervous system tissue constitute a promising avenue for discovering novel HD metabolomics biomarkers (Schnackenberg and Beger 2007).

Longitudinal studies have shown promising results in clinical and imaging HD biomarker discovery, but many of these biomarkers are either expensive or subject to inter-rater variability (Tabrizi et al. 2013). A good biomarker should identify changes before clinical manifestation, should be easily obtained and should respond robustly to disease-modifying interventions. Increasingly, metabolomics technology is used in biomarker studies because it can identify intermediate biomarkers of deregulated genomic pathways (Nishiumi et al. 2014; Wang et al. 2013). Furthermore, metabolomics identifies changes that occur downstream of the gene expression level. This applies particularly well in HD since it is recognized that the mutant protein causes genome wide transcriptional deregulation (Hodges et al. 2006; Runne et al. 2008). The mutant huntingtin protein is ubiquitously expressed, and gene expression deregulations can be found in various HD tissues and organs. Furthermore, metabolite changes in blood may reflect changes in tissues that have been in contact with blood (Diamanti et al. 2013) and as it is impossible to measure molecular biomarkers in the brain, peripheral blood has been proposed as a viable alternative (Sassone et al. 2009). Nonetheless, the cellular heterogeneity of blood together with the data complexity produced by non-targeted mass-spectrometric protocols, make it difficult to quantify the levels of all metabolites simultaneously. Therefore, we have used a targeted metabolomics approach that measures the concentration of a selected group of HD relevant, key biological compounds (such as amino acids, acyl carnitines, hexoses and glycerophospholipids) in a semi-high throughput manner to identify such metabolomics markers.

The aim of this study was to detect metabolic markers of HD status and progression as well as disease deregulated metabolic pathways. Our approach was based on targeted mass-spectrometry using the Biocrates Absolute*IDQ*^TM^ p150 (Romisch-Margl et al. 2012) kit to measure metabolite levels in serum from HD carriers and controls. We then tested for the association of the metabolite levels with HD mutation status, and well accepted clinical progression scores and stages such as the Unified Huntington’s Disease Rating Scale (UHDRS) total motor score (TMS) and the total functional capacity (TFC) score based stages. Since the integration of disparate biological data types like metabolomics and transcriptomics can provide a more complete picture of diseases we correlated our metabolomics data with our publicly available whole genome gene expression profiling data from the same patient cohort and investigated functional relationships between the metabolite changes and the gene expression changes.

## Materials and methods

### Metabolite measurements

Metabolite concentrations were determined using the targeted metabolomics kit Absolute*IDQ*™ p150 (Biocrates Life Sciences AG, Innsbruck, Austria) and flow injection electrospray ionization tandem mass spectrometry (FIA-ESI–MS/MS). A total of 163 different metabolites were quantified simultaneously by the platform in 10 µL serum. The metabolite panel consists of 14 amino acids, Hexose (H1), free carnitine (C0), 40 acylcarnitines (Cx:y), hydroxylacylcarnitines (C(OH)x:y), and dicarboxylacylcarnitines, 15 sphingomyelins (SMx:y) and N-hydroxyacyloylsphingosylphosphocoline (SM(OH)x:y), 77 phosphatidylcholines (PC, aa = diacyl, ae = acyl-alkyl) and 15 lyso-phosphatidylcholines. Lipid side chains are denoted as Cx:y, where x represents the number of carbons in the side chain and y the number of double bonds. The assay procedures of the p150 kit as well as the metabolite nomenclature have been described in detail previously (Romisch-Margl et al. 2012). Sample handling was performed by a Hamilton Microlab STAR^TM^ robot (Hamilton Bonaduz AG, Bonaduz, Switzerland) and an Ultravap nitrogen evaporator (Porvair Sciences, Leatherhead, U.K.), beside standard laboratory equipment. Mass spectrometric (MS) analyses were done on an API 4000 LC–MS/MS System (Sciex Deutschland GmbH, Darmstadt, Germany) equipped with a 1200 Series HPLC (Agilent Technologies Deutschland GmbH, Böblingen, Germany) and a HTC PAL auto sampler (CTC Analytics, Zwingen, Switzerland) controlled by the software Analyst 1.5. Data evaluation for quantification of metabolite concentrations and quality assessment was performed at the Genome Analysis Center of the Helmholtz Zentrum München using the Met*IDQ*™ software package, which is an integral part of the Absolute*IDQ*™ kit. Internal standards served as reference for the calculation of metabolite concentrations in µM.

### Serum collection

Peripheral blood was collected from 29 presymptomatic, 68 symptomatic and 36 control, non-fasting individuals with institutional review board approval and after informed consent. For serum sample isolation, blood was collected in BD vacutainer Z tubes (no additives) and was allowed to clot for 1 h at room temperature. Tubes were spun at 1300 g for 10 min at room temperature, were aliquoted and stored at −80 °C. Detailed information about the UHDRS clinical scores and CAG repeat lengths of all the patients and controls as well as gender age and BMI information can be found in Supplementary File 1.

### Quality controls

To ensure the robustness of downstream statistical analyses, all data provided from the MetIQ software package were subjected to three quality control steps. For the first step the coefficient of variance was calculated for each experimental plate. To achieve this five aliquots of a reference plasma pool were measured on each plate together with the cohort samples. The coefficient of variance was calculated as the standard deviation to mean ratio for all five reference samples per metabolite and per experimental plate. All metabolites with a mean coefficient of variance of all plates, higher than 25 % were excluded from further analysis. All metabolites with a missing value rate larger than 5 % were also excluded. In the second step any outlying data points with a value greater than mean ± 5 SD of all measurements for this metabolite were excluded. Additionally, two Huntington disease samples were excluded due to high BMI values (outliers). For samples with less than, or equal to, three independent outlying points only the independent data points themselves were excluded. After these quality control steps 114 out of 163 metabolites and 133/138 samples remained. After the above steps, when missing values were detected these were imputed using the R package “mice”. Finally, all metabolite concentrations were transformed using natural logarithm and before applying the experimental linear modeling analysis.

### Statistical analysis

To identify significant differences between the HD and control samples, the statistical analysis software R (Version 3.1.2, http://www.r-project.org/) was used. After the metabolite concentrations were log-transformed, linear modelling statistical tests were applied. In specific, in the first model (disease status) the HD mutation carriers’ and control individuals’ groups were coded as the main covariate and tested in a linear model using gender (categorical) age and BMI as additional (continuous) covariates. In the second model (disease severity) a four group categorical variable vector was used as the main covariate. The following groups were defined: Group 1—(n = 36) control, Group 2—(n = 29) pre-symptomatic (TMS score ≤5), Group 3—(n = 31) symptomatic (TMS > 5, TFC score 13–7) and Group 4—(n = 37) advanced symptomatic individuals (TMS score >5, TFC score of 0–6). For both models the final *P* value that was used to judge the validity of our findings was extracted using the ANOVA function on the two nested linear models; the reduced linear model containing only the covariates of gender, age and BMI and the full model additionally containing the main disease group categorical covariate. Since many identified metabolites showed a high degree of correlation (see Supplementary File 2), the Bonferroni method was judged too strict for multiple testing correction. Therefore, the experiment-wide significance threshold that was used was 1.34E−03. This value was calculated using the matrix spectral decomposition method and the eigenvalues of the metabolites correlation matrix (matSpDlite) (Li and Ji 2005; Cheverud 2001; Nyholt 2004). Bar plots for all metabolites that passed quality control were created using the *Platform for RIKEN Metabolomics* (PRIMe) tool for Microsoft excel (Tsugawa et al. 2015). The PLS-DA analysis across controls, presymptomatics and symptomatic HD carriers was performed using the corresponding function of the MetaboAnalyst v.3.0 online tool for metabolomics data (Xia et al. 2015).The top, pair ratio associations for all possible metabolite pair ratios were calculated through log transformation of the ratios and the p-gain value was calculated from the individual *P* values of the ratio metabolites. In specific, p-gain is defined as the fold decrease in the *P* value of association for the pair of metabolites compared to the lower of the two *P* values for the single metabolites (Suhre et al. 2010a).

### Integration of metabolomics with transcriptomics analysis

Gene expression data from our previously published dataset (Mastrokolias et al. 2015) were extracted using the scaled data object from the voom function of the limma package designed for RNAseq data analysis (Law et al. 2014). The data for both the genes and the metabolites were regressed for the effect of age, gender and BMI. The gene expression data were regressed for cellular hemoglobin percentage (hemoglobin alpha and beta sequencing count tags) as a proxy of the cell reticulocyte count (Mastrokolias et al. 2012). For the extraction of the genes that were related to the metabolites the metabolic pathway databases from the Kyoto Encyclopedia of Genes and Genomes KEGG (Kanehisa et al. 2012) (release 63) and BioCyc (version 16) were accessed for retrieving background knowledge for each metabolite(Dharuri et al. 2013). Two interrogation schemes were employed: pathway scheme and reaction scheme. In a pathway scheme, for a given metabolite, all the pathways that it participated in were determined followed by the retrieval of all the genes that participated in these pathways. In a reaction scheme, given a metabolite, all the reactions that it was part of and the compounds that participated in these reactions were determined (Dharuri et al. 2013).

For the integrated metabolomics and transcriptomic pathway analysis, the community driven resource of curated pathways WikiPathways (Kutmon et al. 2015) was used to identify common pathways. The WikiPathways human pathway collection is the largest and most active collection per species. In terms of coverage of unique human genes, WikiPathways is comparable to KEGG. To investigate which metabolite-gene pathways overlapped we used all 10 significant metabolites (8 phosphatidylcholines and 2 amino acids) and the top 200 genes from our above linear modeling, as the input to the WikiPathways Web Service. Since pathway information for individual phosphatidylcholines is lacking, we also included the compounds at the phosphatidylcholine compound class level and their isomers (1,2-diacyl-sn-glycero-3-phosphocholine, alkyl,acyl-sn-glycero-3-phosphocholine and 1,2-diacyl-sn-glycero-3-phosphocholine(1+)) according to the Chemical Entities of Biological Interest ontology (Hastings et al. 2013).

## Results

### The metabolomic dataset

**Fig. 1:**
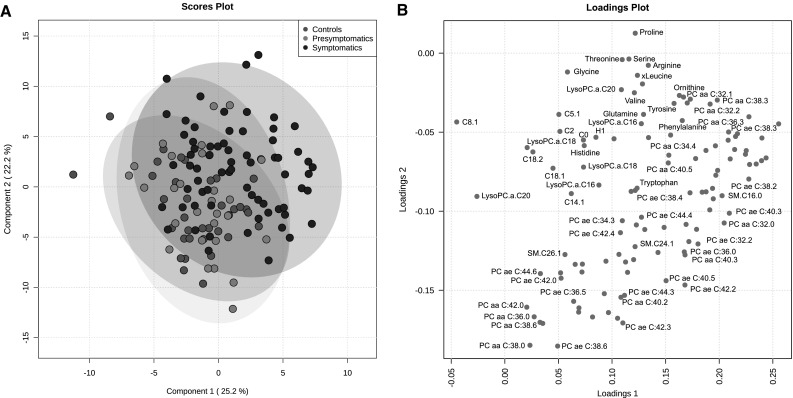
*Diagram* representing principal component analysis performed using Metaboanalyst v 3.0. **a** Principal component analysis results represent HD presymptomatic, symptomatic patients and control group separation based on the *top* 2 most significantly contributing components. *Colored circles* represent 95 % confidence intervals. *Colored dots* represent individual samples. **b** *Loading* plots of the first two principal components for the platform metabolites. Some metabolite names have been omitted next from their corresponding metabolite symbol for figure clarity purposes

**Fig. 2:**
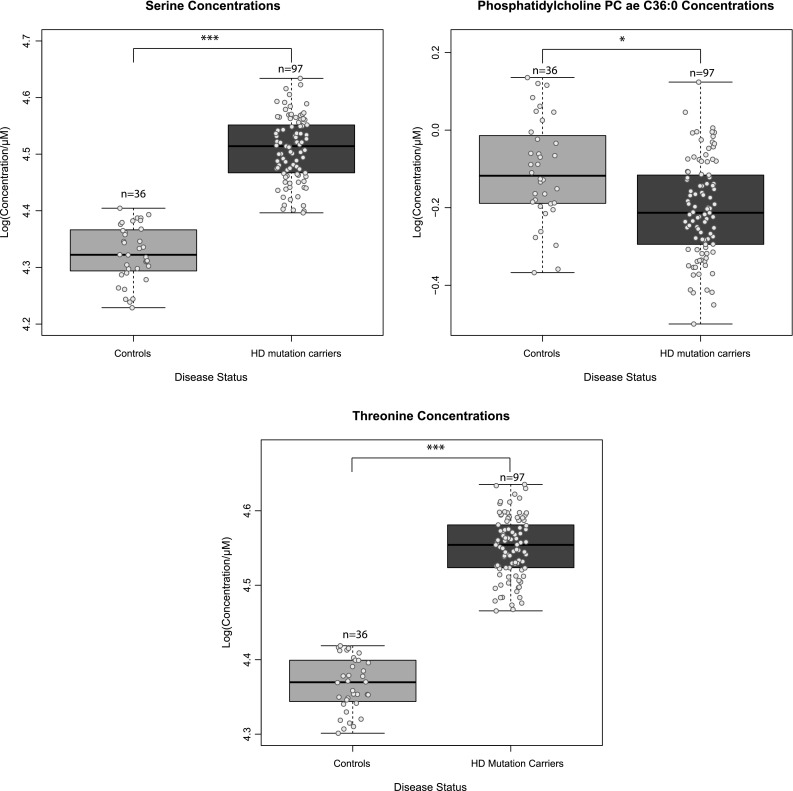
*Boxplots* of concentration levels of metabolites that were significantly different between control individuals and HD mutations carriers. *Numbers* represent the group sizes and asterisks represent significance values from linear modelling analysis. *Colored dots* represent individual sample concentrations. *Asterisks* represent significance probability values [Pr(>F)] from the ANOVA calculation of the single (full—see methods) linear model accounting for disease status, gender, age and BMI. *P value <0.05, ***P value <0.001

**Fig. 3:**
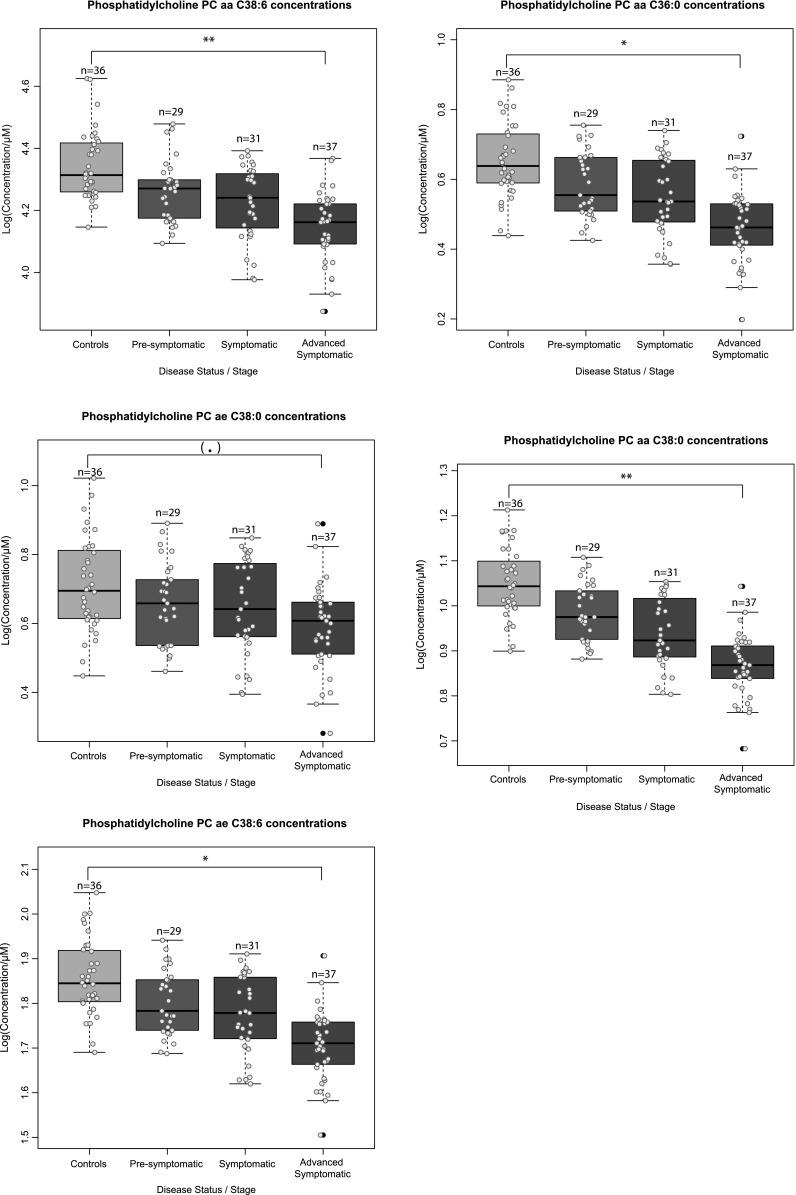
*Boxplots* of concentration levels of significant metabolites between 4 groups—controls, presymptomatic, symptomatic and advanced symptomatic HD mutation carriers. *Numbers* represent the group sizes and *asterisks* represent significance values from linear modelling analysis. *Colored dots* represent individual sample concentrations. *Black dots* represent outliers. *Asterisks* represent significance probability values [Pr(>F)] from the ANOVA calculation of the single (full—see methods) linear model accounting for disease stage group, gender, age and BMI. (.) *P* value <0.1, *P value <0.05, **P value <0.01

Using the Biocrates p150 kit we quantified serum concentrations of 163 metabolites in 133 serum samples from 97 HD mutation carriers and 36 controls. After quality control, 114 of the initial 163 metabolites could be reliably detected and these were used for further analysis. These 114 metabolites consisted of 14 amino acids, 7 carnitines, 10 lyso-phosphatidylcholines, 69 phospatidylcholines, 5 hydroxysphingomyelins, 8 sphingomyelins and 1 hexose. Supplementary File 3 contains data distributions of all 163 metabolites, the metabolites that were excluded and their concentrations relative to the platform limit of detection (LOD) and lower limit of quantification (LLOD). In order to analyze the group structure of the metabolomic dataset across controls, presymptomatic and symptomatic HD patients, we performed a partial least square discriminant analysis (PLS-DA). The symptomatics group (group 3 and 4 combined, TMS >5) exhibited a clear shift from the control (group 1) and the pre-symptomatic (group 2) samples. The scores plot for the symptomatics, pre-symptomatics and control groups and for the first two principal components can be seen in Fig. 1a while the relative contributions and the relationships between the metabolites can be seen in the loadings plot of Fig. 1b. The observed concentration levels for all 114 metabolites across all four groups can be seen in Supplementary File 4. To identify which metabolites were significantly different in HD, the concentration changes of the detected metabolites were tested using a linear regression model between HD mutation carriers versus control individuals and a linear regression model using four disease severity stage groups as described in the Sect. 2. We identified 3 metabolites significantly changed in the HD mutation carriers versus controls analysis and 8 metabolites significantly changed in the 4 disease stage group analysis that associated with disease progression (adj.*P*.val < 1.34E−03) (see Table 1). In the two group analysis the amino acids serine and threonine were higher in HD mutation carriers while the phosphatidylcholine acyl-alkyl C 36:0 average level was lower (see Fig. 2). In the 4 group analysis, 8 metabolites in total passed the significance threshold. These 8 metabolites were exclusively acyl alkyl and di-acyl phosphatidylcholines and were lower in HD versus controls and associated with increasing disease progression (for the top 5 metabolites see Fig. 3). In the 4 group analysis serine was also in the top 10 metabolites but failed to pass the adjusted *P* value significance level.

**Table 1:** Table of the top 10 metabolites resulting from linear modeling analysis of metabolite concentrations between HD mutation carriers and controls and accounting for disease status (top) or disease progression group (bottom) and gender, age and BMI

Metabolite ID	HD mutation carriers versus controls analysis	*P* value	Concentration change
**Ser**	**Serine**	**3.08E−05**	**Higher in HD**
**PC ae C36:0**	**Phosphatidylcholine acyl-alkyl C 36:0**	**6.61E−05**	**Lower in HD**
**Thr**	**Threonine**	**1.02E−03**	**Higher in HD**
PC ae C42:0	Phosphatidylcholine acyl-alkyl C 42:0	2.35E−03	Lower in HD
PC ae C44:3	Phosphatidylcholine acyl-alkyl C 44:3	3.72E−03	Lower in HD
PC aa C38:6	Phosphatidylcholine diacyl C 38:6	4.60E−03	Lower in HD
PC ae C38:0	Phosphatidylcholine acyl-alkyl C 38:0	8.95E−03	Lower in HD
Arg	Arginine	1.80E−02	Higher in HD
PC aa C36:0	Phosphatidylcholine diacyl C 36:6	1.99E−02	Lower in HD
PC aa C40:6	Phosphatidylcholine diacyl C 40:6	2.33E−02	Lower in HD

Metabolite ID	Disease progression (4) group analysis	*P* value	Concentration change
**PC aa C38:6**	**Phosphatidylcholine diacyl C 38:6**	**1.01E−04**	**Lower in HD**
**PC aa C36:0**	**Phosphatidylcholine diacyl C 36:0**	**4.22E−04**	**Lower in HD**
**PC ae C38:0**	**Phosphatidylcholine acyl alkyl C 38:0**	**4.42E−04**	**Lower in HD**
**PC aa C38:0**	**Phosphatidylcholine diacyl C 38:0**	**5.48E−04**	**Lower in HD**
**PC ae C38:6**	**Phosphatidylcholine acyl alkyl C38:6**	**5.63E−04**	**Lower in HD**
**PC ae C42:0**	**Phosphatidylcholine acyl alkyl C 42:0**	**1.06E−03**	**Lower in HD** ^a^
**PC aa C36:5**	**Phosphatidylcholine diacyl C 36:5**	**1.16E−03**	**Lower in HD** ^b^
**PC ae C36:0**	**Phosphatidylcholine acyl alkyl C 36:0**	**1.19E−03**	**Lower in HD** ^a^
PC ae C40:1	Phosphatidylcholine acyl alkyl C40:1	1.72E−03	Lower in HD
Ser	Serine	1.96E−03	Higher in HD

*P* values represent significance probability values [Pr(>F)] from the two-way ANOVA calculation on two (nested) linear models accounting for disease status or disease stage respectively (measurement variables) and gender, age and BMI (nominal variables). Metabolites that pass the adjusted *P* value threshold are highlighted in bold. Concentration changes were obtained from the fitted data of the metabolites using the full linear statistical model (see above) and disease state and stage respectively as the main covariate

^a^Upper quartile range higher in earlier HD symptomatics (group 3) versus HD presymptomatics (group2)

^b^Higher in earlier symptomatics

### Association of metabolite pair ratios with disease status and severity

Previous studies have shown that calculating the ratios of individual metabolite concentrations can reduce dataset variation. Furthermore, such metabolite ratio changes have been connected to altered enzymatic reactions and pathways, can be used as an approximation of the associated enzymatic activity (Petersen et al. 2012; Gieger et al. 2008) and ratios of specific pairs of metabolites have been suggested as biomarkers (Ceglarek et al. 2002; Perdelli et al. 2002). For this reason, we calculated all the pairwise ratios of the detected metabolites and tested for the association of their ratios using the same two linear models. The resulting associations were ranked according to their p-gain values. The results for the two group (HD vs. controls) analysis and the four group analysis can be seen in Supplementary File 5. We observed that in the two group analysis the results were dominated by inter-phosphatidylcholine ratios, as well as ratios of phosphatidylcholines to serine and threonine. Additionally, one of the top p-gain values was that of the arginine to carnitine ratio. Most of the ratios of the sphingolipids and sphingolipids to amino acids were lower in HD carrier samples. The metabolite pair associations with controls, pre-symptomatic and the 2 symptomatic groups revealed similar results. The highest p-gain values were exhibited by inter-phosphatidylcholine ratios but also phosphatidylcholines and hydroxy-sphingomyelin C16:1 (SM.OH.C16:1). The 4 group disease progression analysis was also characterized by the absence of any amino acids in the top p-gain analysis similar to the individual metabolite analysis. These results confirm the the changes in phosphatidylcholines levels in the disease and strengthen the potential of the use of (pairs of) phosphatidylcholines as markers of disease progression since variation is reduced.

### Integration of metabolomics with transcriptomics

**Fig. 4:**
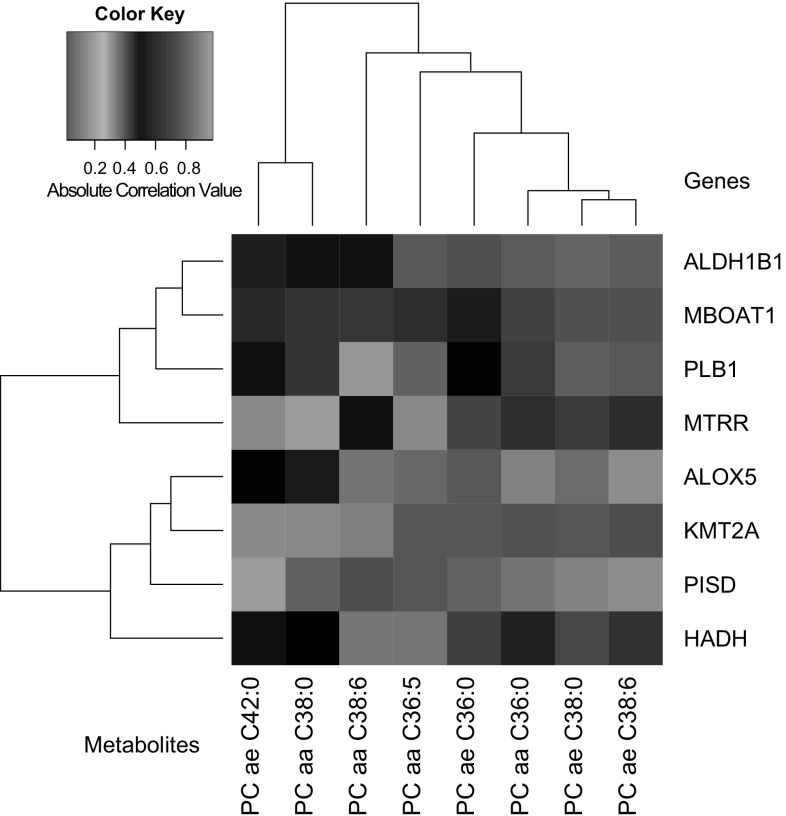
Heatmap of correlation values between gene expression levels and phosphatidylcholines metabolite concentrations. The selected genes shown here are genes identified using our previous gene expression data and that participate in phosphatidylcholine KEGG and BioCyc pathways and reactions. Phosphatidylcholines shown here are the statistically significant phosphatidylcholine metabolites identified from the 4 group linear modelling analysis. *Color key* represents absolute correlation values

**Fig. 5:**
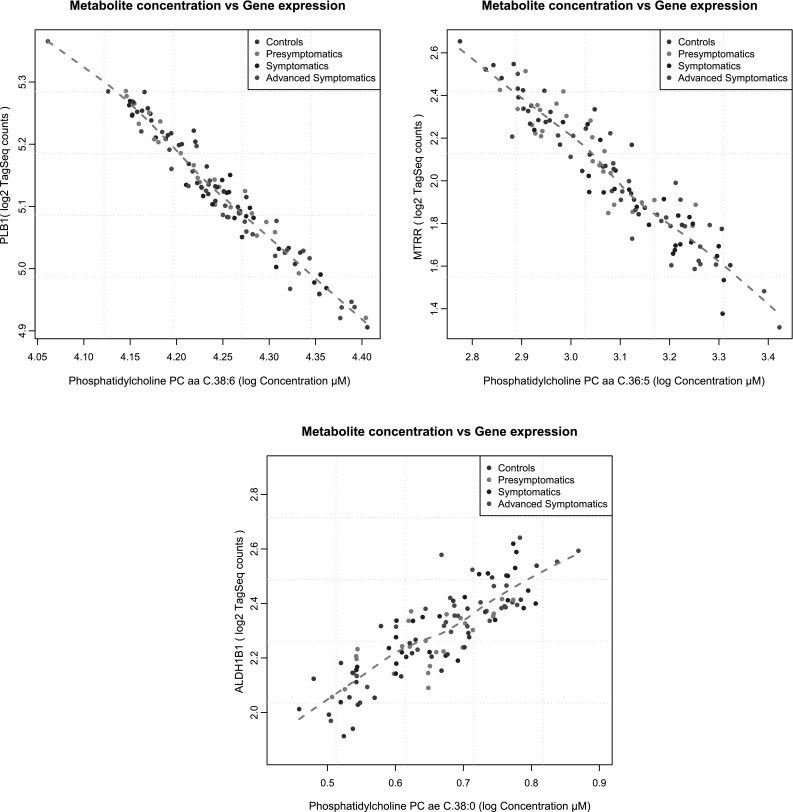
Plots of selected phosphatidylcholine metabolites versus their corresponding gene that participates in a phosphatidylcholine pathway or reaction. The 3 plots represent the most highly correlated metabolite to gene pairs from the integration of metabolites to gene expression data analysis. *Different colored dots* represent individual samples from each disease state group and *brown lines* represent loess fit lines

To further explore potential molecular connections of the identified metabolites with HD relevant or novel disease state/progression mechanisms we combined our targeted metabolomics dataset with our previously published next-generation sequencing gene expression data from the same patient and control cohort (Mastrokolias et al. 2015). For our initial analysis we focused on the phosphatidylcholine metabolites since these exhibited significant statistical associations with HD disease progression scores. Using the previously published methodology of Dharuri et al. (2013) we extracted the genes from the KEGG (Kanehisa et al. 2010) and BioCyc (Romero et al. 2005) databases that corresponded to phosphatidylcholine related metabolic pathways. Consequently, we reanalyzed the previously published gene expression data from the same cohort, using the same linear model with the metabolomics dataset. We extracted the top 200 differentially expressed genes and compared them with the phosphatidylcholine pathways related genes from the KEGG and BioCyc databases. We identified 8 genes that were present in both the differentially expressed gene list and the above two databases lists. These genes were *ALOX5* (arachidonate 5-lipoxygenase)*, ALDH1B1* (aldehyde dehydrogenase 1 member 1)*, KMT2A* (lysine specific methyltransferase 2A)*, MBOAT1* (membrane bound O-acyltransferase DC1), *MTRR* (methionine synthase reductase)*, PISD* (phosphatidylserine decarboxylase)*, PLB1* (phospholipase B1) and *HADH* (hydroxyacyl-CoA dehydrogenase). The correlation values of each of the 8 genes with the 8 significant metabolites from our 4 group linear modeling analysis are represented in Fig. 4. We observed that for the genes *MBOAT1*, *PLB1*, *ALDH1B1* and *MTRR* the correlations with the majority of the 8 metabolites were high (r > 0.6) while for the other 4 genes the correlations were average or poor. The highest associations were observed between the genes *ALDH1B1*, *MTRR* and *PLB1* with phosphatidylcholines PC ae C.38:0, PC aa C36:5 and PC aa C38:6 (see Fig. 5). Additional genes that were present both in the BioCyc and KEGG databases and our previous sequencing-based gene expression gene lists and for the amino acid serine were also *NPL* (N-acetylneuraminate pyruvate lyase), *PGLYRP1* (peptidoglycan recognition protein 1) and *TKTL1* (transketolase-like 1). Finally, for the serine and threonine metabolites we could not identify any unique common genes. It should be noted however that the above phosphatidylcholine related gene *ALDH1B1* was also present in our threonine KEGG reaction list and similarly *PISD* and *PLB1* were also present in our serine KEGG reaction list.

To expand on the above findings, we performed a second analysis using the WikiPathways Web Service and all 10 significant metabolites (8 phosphatidylcholines and 2 amino acids) from both of the above metabolomics linear models, in order to investigate further connections between potential metabolomics and transcriptomic pathways. The metabolite-gene pathways with the highest overlap of genes and metabolites we identified were glycerophospholipid biosynthesis (containing genes *PLB1*, *PISD* and Serine and 1,2-diacyl-sn-glycero-3-phosphocholine (1+)) and phase II conjugation (containing gene *MTRR* and serine and threonine), supporting the results from our first pathway analysis. All the overlapping pathways reported for the Wikipathways analysis can be seen in Supplementary File 6.

## Conclusions

The current study targeted approach of the Biocrates technology has been successfully applied in many cohort studies (Draisma et al. 2015; Vouk et al. 2016; Illig et al. 2010). Comparison of this type of data with data obtained from non-targeted platforms has shown strong positive correlations for metabolites named for the same compounds. Furthermore such a comparison has shown that the results obtained are complementary and informative for future studies of comprehensive metabolomic analyses with different platforms (Suhre et al. 2010b; Yet et al. 2016).Using a well-defined, UHDRS-based linear model, we discovered a total of 10 metabolites whose concentrations showed significant associations with Huntington’s disease state and severity stages. Eight of the 10 metabolites were phosphatidylcholines while the other two were the amino acids serine and threonine. These results are in agreement with the results of Tsang et al. that have reported a decrease of phosphatidylcholine levels in frontal cortex lipid extracts of a 3-NP treated HD rat model (Tsang et al. 2009). Phosphatidylcholine is a major membrane phospholipid and has been shown to have a role in neuronal differentiation and cell fate determination (Marcucci et al. 2010). In the past, oral administration of lecithin and other choline containing dietary sources have been suggested as a replacement therapy for HD and as a potential substrate source for brain acetylcholine synthesis (Rosenberg and Davis 1982). The current study shows an increase of serine and threonine in HD patients as shown from the HD versus controls linear modeling. Serine has an important role in the metabolism of purines and pyrimidines since it is the precursor of several other amino acids. It is also a precursor to numerous other metabolites, including sphingolipids and folate, which is the principal donor of one-carbon fragments in biosynthesis. As such, one explanation for the increased serine levels could be that in Huntington’s disease these amino acids are intended for the production of phospholipids whose levels are decreasing with disease severity. Moreover, the d-serine amino acid isomer can act as a neuromodulator since it can activate NMDA receptors. NMDA receptors have been implicated in a range of processes including memory, learning and development and their excessive stimulation can be involved in a number of neurodegenerative conditions including HD (Hardingham and Bading 2010).

The second of the two amino acids whose levels were altered in HD, threonine, is an essential amino acid and together with serine constitute the only two proteinogenic amino acids. Threonine can be converted to pyruvate while in an intermediate step it can undergo thiolysis to produce acetyl-coA. In a less common pathway threonine can also be converted to a-ketobutyrate via serine dehydratase. It has been previously suggested that pyruvate can have a neuroprotective effect in neurological diseases by, among others, enhancing brain to blood glutamate efflux, scavenging H_2_O_2_ and having an anti-inflammatory action (Zilberter et al. 2015). Furthermore it has been shown that pyruvate administration can have a neuroprotective effect in a quinolinic acid rat model of HD (Ryu et al. 2003). Additionally, the inability of the excess threonine to undergo thiolysis and produce acetyl-CoA could result in reduced energy production (Krebs cycle) as well as an insufficient synthesis of acetylcholine. The increased levels of threonine in the mutation carriers could therefore represent a compensatory mechanism in an attempt to produce more substrates for the generation of the above neuroprotective molecules such as pyruvate and/or the inability of the threonine metabolizing enzymes to properly process the present levels of this amino acid.

Using the pairwise combination of all the individual metabolites we discovered a series of metabolites ratios (mainly phosphatidylcholines) that changed gradually with disease severity. These consisted of changes in inter acyl-alkyl-phosphatidylcholines ratios but also changes in the sphingomyelins to phosphatidylcholines ratios. Sphingomyelins are a group of sphingolipids found in mammalian cell membranes and especially membranes that surround nerve cell axons (Slotte 2013). A decreased ratio of sphingomyelins to lipids across disease stages could indicate an increased vulnerability and damage of nerve cell axons. Even though the role of sphingolipids and gangliosides in brain damage has been investigated since the 1970s (Wherrett and Brown 1969; Higatsberger et al. 1981; Heipertz et al. 1977) it is not until recently that strong evidence has been presented in support of the role of gangliosides and their biosynthetic genes in autophagic and apoptotic signaling (Takamura et al. 2008; Desplats et al. 2007). Additionally, the ratio of arginine to carnitine metabolites was among the top results for the two group (HD vs. controls) metabolite pair analysis with modest p-gain values. Arginine is a non-essential amino acid that is also a precursor of nitric oxide (NO) a molecule involved in neurotransmission and inflammation, both of which processes are thought to be deregulated in HD (Andre et al. 2016). It has been previously postulated that increased dietary l-arginine could accelerate motor symptom and weight loss events in HD models, through changes in cerebral blood flow and the regulation of NO and nitric oxide synthase (Deckel et al. 2000; Deckel 2001). Furthermore it has been also shown that arginine uptake by HD patients separated them in two distinct metabolic profile groups indicative of a complex and idiomorphic function of this molecule across different individuals (Salvatore et al. 2011). Since the levels of arginine were already found higher in HD in the individual metabolite analysis, the increased arginine to carnitine ratio in HD patients could indicate an arginine-concomitant decrease in the levels, of the antioxidant and lipid regulator molecule, of carnitine. This is further supported by the study of Cuturic et al., who showed that catabolism and chronic anticonvulsant administration in HD institutionalized patients predisposed to low serum carnitine and that supplementation with levocarnitine improved motor and cognitive measures in these patients (Cuturic et al. 2013). Finally apart from their potential roles in deregulated HD molecular pathways these ratios could also serve as potential biomarkers of disease severity/progression since by calculating individual metabolite ratios the dataset variation is reduced and the biomarker robustness is increased.

Moreover, we integrated a previously published gene expression data with the current metabolomics dataset from the same cohort. In specific by generating bioinformatics workflow-based metabolite specific gene sets we identified a group of 8 genes that were decreased in phosphatidycholine metabolic pathways and also found deregulated in our HD patients. Three of these transcriptomics deregulated genes (*MTRR*, *PLB1* and *ALDH1B1*) exhibited especially high correlation with specific diacyl and acyl-alkyl phosphatidylcholines that were downregulated in HD in the metabolomics dataset. More specifically, *MTRR* is involved in the proper function of methionine synthase and folate metabolism (Wolthers et al. 2007; Leclerc et al. 1998). Mutations in the *MTRR* gene are thought to be responsible for multiple disorders and especially those affected through the deregulation of the folate cycle and homocysteine metabolism (Mitchell et al. 2014; Mandaviya et al. 2014). In the past, increased levels of plasma total homocysteine have been found in HD patients and it has been hypothesized that these increased homocysteine levels are a contributing factor to neurodegeneration in these patients (Andrich et al. 2004). The second of the three genes whose expression was highly correlated with metabolite levels, PLB1 is a membrane-associated phospholipase with phospholipase A2 activity that exhibits preferential hydrolysis at the sn-2 position of diacyl-phospholipids. A recent study by Fonteh et al. and in Alzheimer’s disease patients cerebrospinal fluid has shown that a significant increase in this phospholipase A2 activity accompanies the glycerophospholipid decrease observed in late onset AD patients (Fonteh et al. 2013). This is in agreement with our data since the levels of PLB1 exhibited an inverse correlation with all 8 of the metabolites that were statistically significantly associated with HD progression. Thus in a similar fashion with the findings of Fonteh et al. the increased PLB1 phospholipase A2 expression levels in our HD blood samples could be indicative of perturbation of membrane structures with a concomitant disruption of cellular transport and clearances processes as well as a resulting inflammation overactivation (Stephenson et al. 1996; Sun et al. 2004). Finally, this integrated “-omics” analysis showed that potential pathways affected from the deregulation of the above genes and changed metabolite concentrations were glycerophospholipid biosynthesis, vitamin B12 and folate metabolism. It has been previously shown that low choline and folate levels are interrelated and that the de novo synthesis of phosphatidycholine is insufficicent to maintain choline levels when the levels of the previous two compounds are also low (Jacob et al. 1999). Low folate has been associated with cardiovascular disease, a pathology that also affects HD patients and according to some surveys is the leading cause of death in patients (Abildtrup and Shattock 2013; Mihm et al. 2007). The administration of choline has been shown to reduce total plasma homocysteine levels (Olthof et al. 2005), an indicative cardiovascular disease risk factor, while folate and vitamin B12 supplementation has been considered as an additional supplementation therapy for many neuropsychiatric disorders (Stanger et al. 2009; Kifle et al. 2009).

Novel findings from our study include the serum upregulation of serine and threonine levels as well as the inverse association of the levels of a group of 8 phospatidylcholine metabolites with disease progression. The lower level of these metabolites support the evidence found regarding altered lipid metabolism in neurodegenerative disorders as well as the use of phosphatidylcholine as a potential therapeutic avenue (Growdon 1987; Adibhatla and Hatcher 2008; Adibhatla et al. 2006). The increased amino acid level findings are in partial agreement with an older study that also identified increased serine levels but instead in the Broadmann’s area 10 of HD patients (Bonilla et al. 1988). On the other hand, these results are in contrast with the findings of a study by Gruber et al. that reported decreased levels of serine and 4 more amino acids in HD mutation carriers, in plasma samples (Gruber et al. 2013). Previous studies by Mochel et al. have identified valine, leucine and isoleucine metabolite levels to be decreased in plasma samples of HD patients versus presymptomatic and control individuals (Mochel et al. 2007, 2011). We could not validate this finding in our serum samples using the Biocrates platform. A possible explanation for this could be the different platforms and protocols that were used to measure the metabolites. Additionally, the differences could be attributed to the different group sizes and the different UHDRS score thresholds that were used to differentiate between presymptomatic, early and mild HD patient groups. Another potential limitation or reason in regard to the disagreement of some our results with previous studies could be that our study was performed using serum samples while the previous studies were performed using plasma In specific, using the Biocrates platform employed in the current study it has been shown that serum exhibits higher sensitivity than plasma due to the fact that metabolite concentrations are generally higher in serum samples (Yu et al. 2011; Kronenberg et al. 1998). An additional limitation of the study could be the potential effect of drug treatment on the metabolomics profile of the individuals used for the current study, which was not taken into account since this information was not available for all study participants (especially controls). Considering the great disease phenotypic variation and the different progression rates that characterize Huntington’s disease mutation carriers our results will require further validation and refinement in even larger groups before they are used in a clinical trial setting. Such additional validation experiments can reduce the intergroup metabolite concentration overlap and clearly define the concentration thresholds that can be used to distinguish between disease progression/stages. Finally, further research would have to be performed to determine if the current metabolic changes are specific for Huntington’s disease or might also partly track changes in other similar neuromuscular disorders and could therefore have additional potential diagnostic applicability.

The present study is according to our knowledge the first study that uses a targeted metabolomics approach in peripheral blood serum samples and in such a large cohort of HD patient peripheral blood samples, with so many pre-symptomatic patients. Obtaining a disease specific metabolomic profile of HD could greatly improve our understanding of the disease pathology. Additionally, these profiles can potentially be used for patient screening as well as drug safety and effectiveness assessment. This could allow for earlier diagnosis something which is very important for HD where disease progression rates and clinical evaluation scores can be highly variable. Serum samples can also be collected noninvasively allowing for longitudinal studies as well as their use both in the preclinical and clinical settings. Our findings combined with the reproducibility and standardization of platforms such as the one used in this study demonstrates the potential of metabolomics to identify disease changes as well as prospective disease progression biomarkers.

## Electronic supplementary material

Below is the link to the electronic supplementary material.

Supplementary material 1 (DOCX 16 kb)

Supplementary material 2 (PDF 240 kb)

Supplementary material 3 (PDF 2398 kb)

Supplementary material 4 (PDF 392 kb)

Supplementary material 5 (DOCX 18 kb)

Supplementary material 6 (DOCX 13 kb)

Supplementary material 7 (DOCX 12 kb)
