# Supplementary material for: Integration of targeted metabolomics and transcriptomics identifies deregulation of phosphatidylcholine metabolism in Huntington’s disease peripheral blood samples
Source: Metabolomics. 2016 Jul 27;12:137. doi: 10.1007/s11306-016-1084-8 (PMC4963448; doi:10.1007/s11306-016-1084-8)
Supplement: Supplementary file 1 — Supplementary material 1 (DOCX 16 kb) [file 11306_2016_1084_MOESM1_ESM.docx]

**Supplementary File 1** Cohort characteristics for the peripheral blood serum samples used for Biocrates targeted metabolomics profiling.

| **Cohort Groups**  Total n = 133 | | **Gender**  **(n)** | **CAG**  **Repeat**  **Mean** | **Age Mean (Range)^1^** | **TMS^2^ Mean (Range)** | **TFC^3^ Mean (Range)** | **BMI^4^ Mean (Range)^a^** |
| --- | --- | --- | --- | --- | --- | --- | --- |
| Group 1 (n=36)  **Controls**  (CAG ≤ 35) | | Female  (19) | 21.1 | 41.6 (25-65) | 1.3 (0-7) | 12.7 (11-13) | 24.6 (20-32) |
|  |  | Male  (17) | 21.3 | 39 (23-62) | 1.1 (0-8) | 12.9 (12-13) | 25.5 (19-31) |
| **HD** | Group 2 (n=29)  **Pre-symptomatics**  (TMS ≤ 5) | Female  (17) | 43.1 | 42.2 (26-60) | 2.8 (0-5) | 12.1 (8-13) | 24.8 (20-33) |
|  |  | Male  (12) | 41.6 | 44.2 (32-60) | 2.3 (0-5) | 12.3 (7-13) | 23.9 (21-27) |
|  | Group 3 (n=31)  **Symptomatics**  (TMS >5, TFC 13-7) | Female  (14) | 43.9 | 52.5 (23-64) | 22.4 (8-49) | 10.9 (8-13) | 23.7 (18-30) |
|  |  | Male  (17) | 43.7 | 51.8 (33-74) | 19.1 (7-50) | 10.8 (7-13) | 25.9 (21-32) |
|  | Group 4 (n=37)  **Advanced Symptomatics**  (TMS >5, TFC 6-0) | Female  (23) | 45.1 | 51.3 (33-76) | 50.8 (7-89) | 3.1 (0-6) | 24.5 (17-32) |
|  |  | Male  (14) | 44.9 | 54.9 (32-69) | 51.3 (8-86) | 2.4 (0-5) | 26.4 (16-33) |

^1^ Range values have been rounded to the nearest whole number.

^2^TMS == Total Motor Score rating (0-124) of the unified Huntington’s disease rating scale (UHDRS).

^3^ TFC == Total Functional Capacity scale of Huntington’s disease patients (13-0).

^4^BMI==Body mass index
