# Supplementary figures and images for: Integration of targeted metabolomics and transcriptomics identifies deregulation of phosphatidylcholine metabolism in Huntington’s disease peripheral blood samples
Source: Metabolomics. 2016 Jul 27;12:137. doi: 10.1007/s11306-016-1084-8 (PMC4963448; doi:10.1007/s11306-016-1084-8)

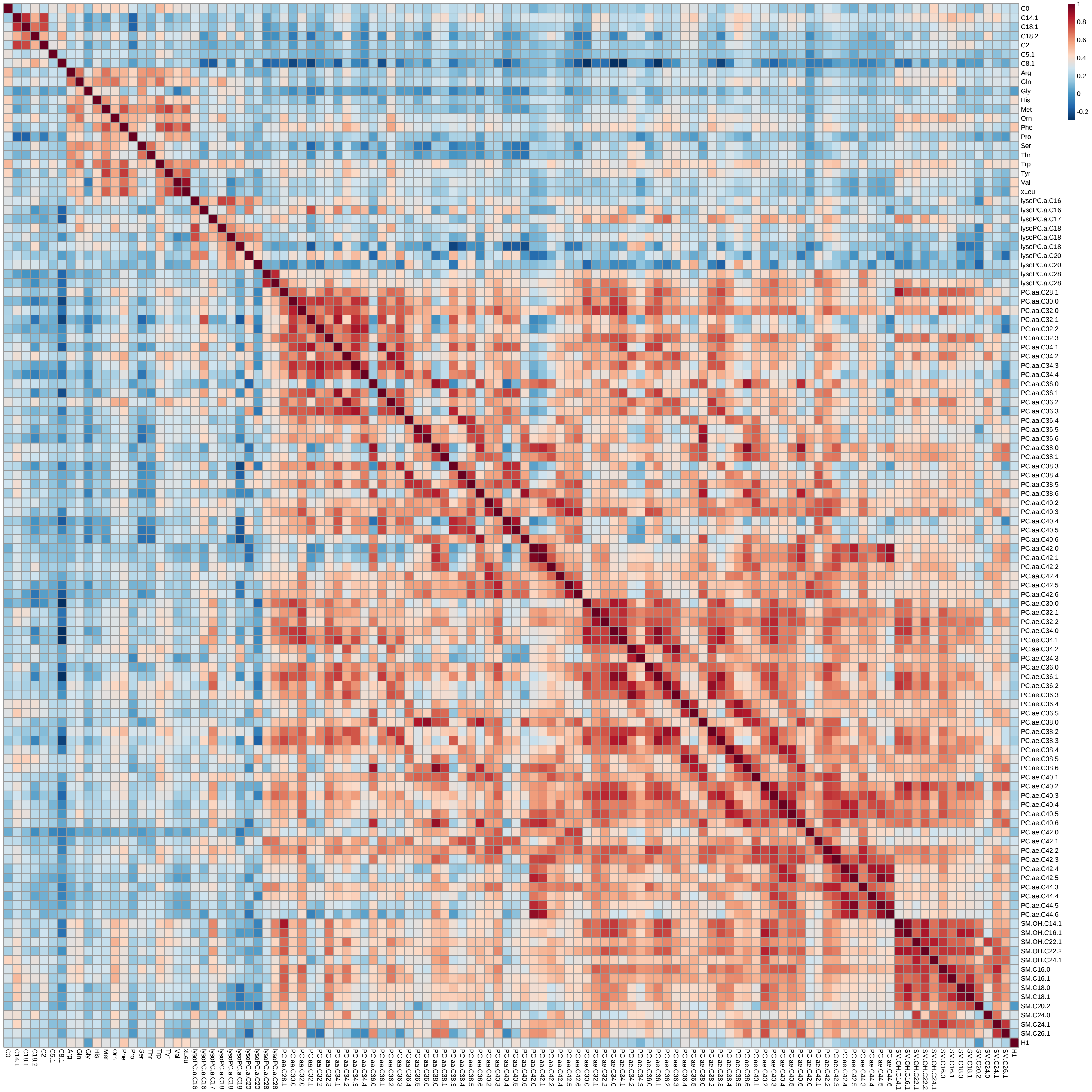

Supplement: Supplementary file 2 — Supplementary material 2 (PDF 240 kb) [file 11306_2016_1084_MOESM2_ESM.pdf]

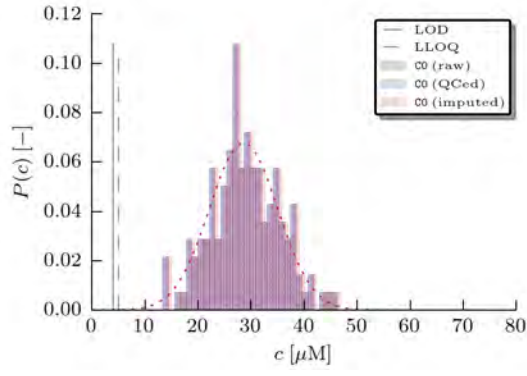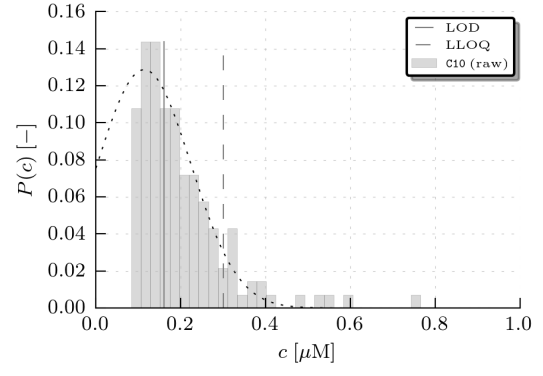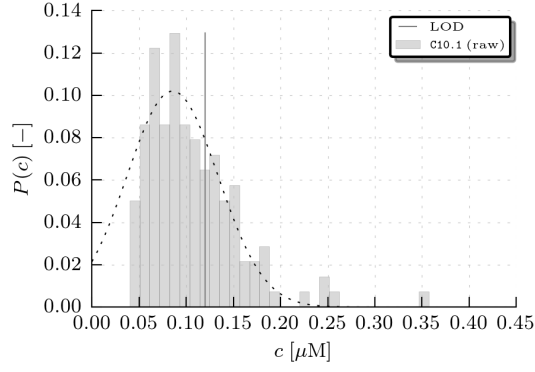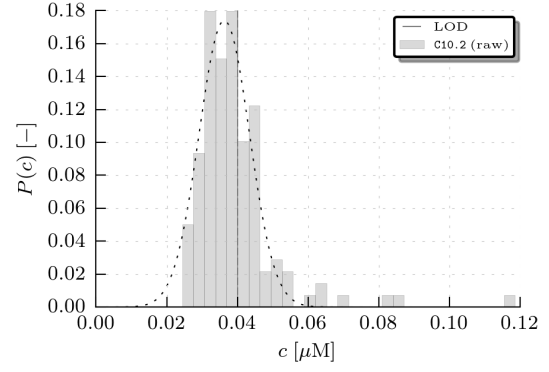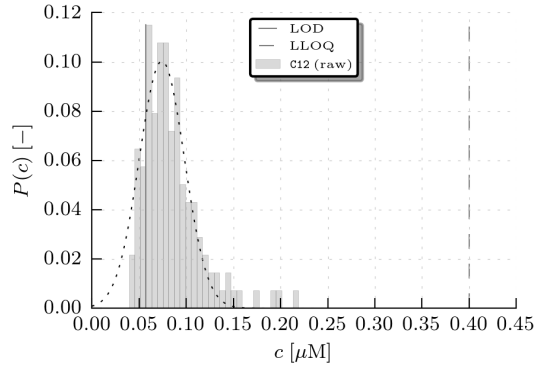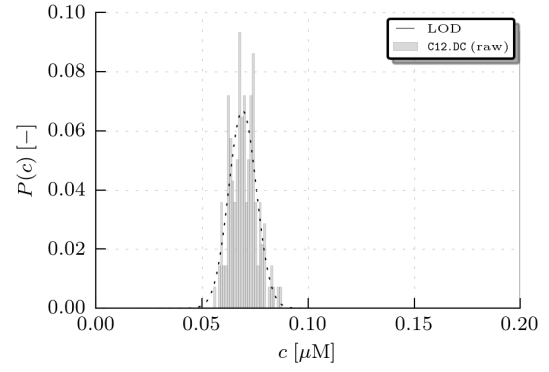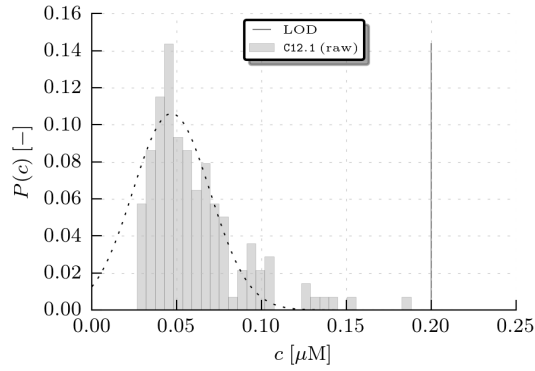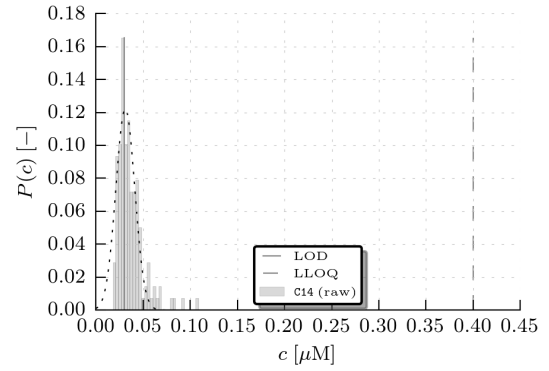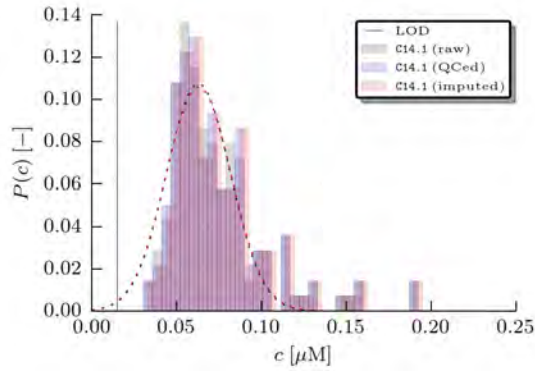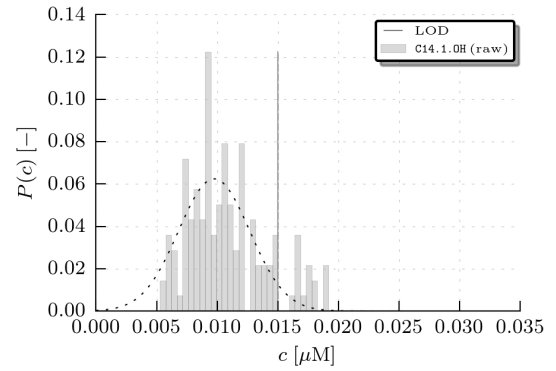

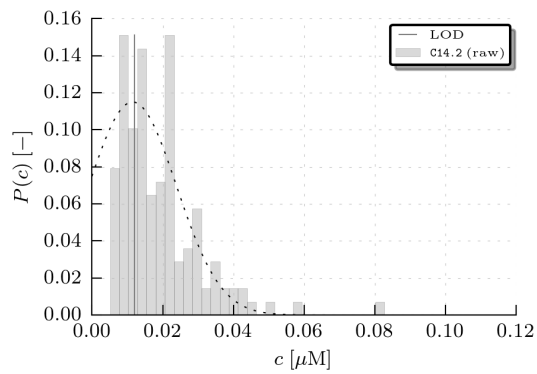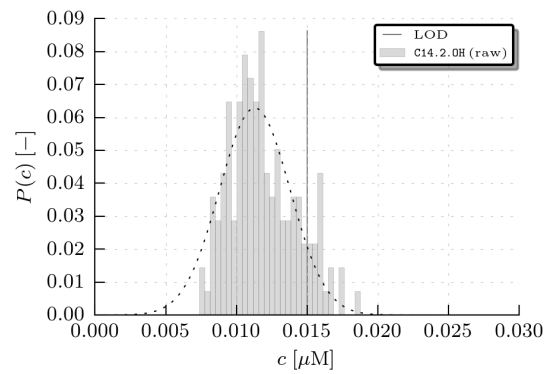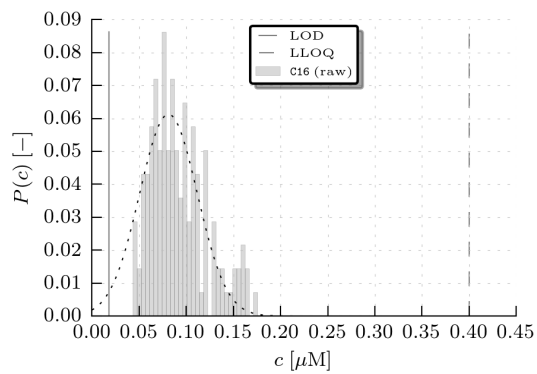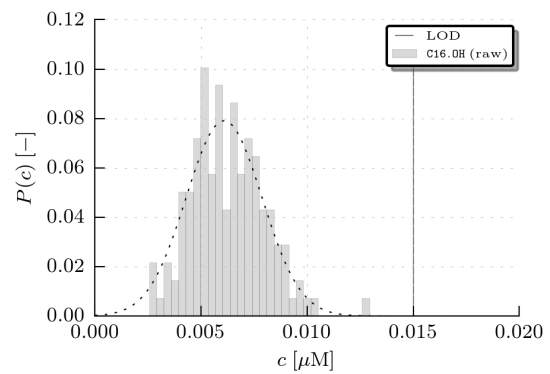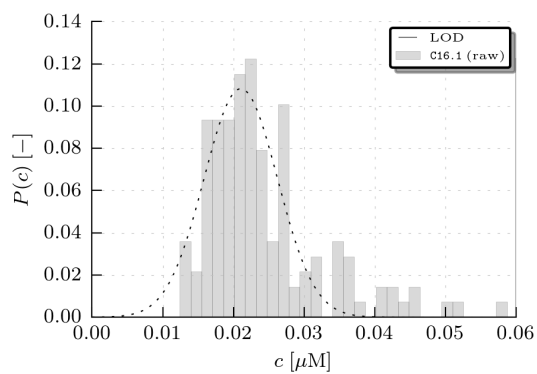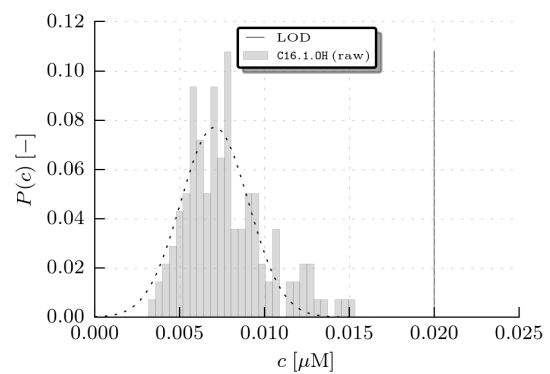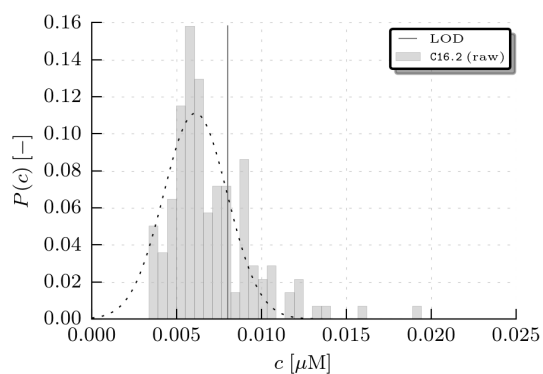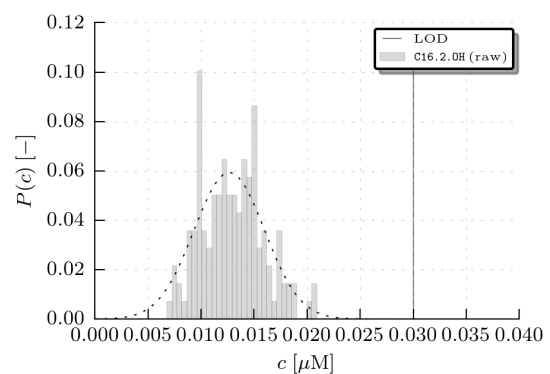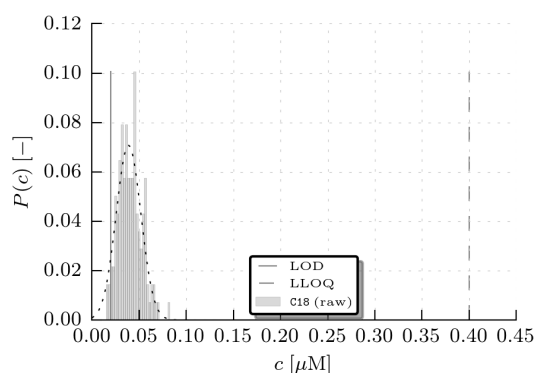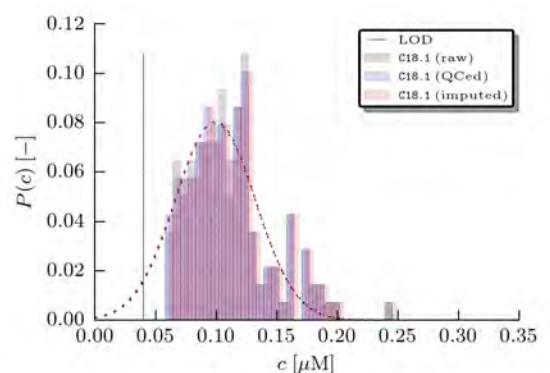

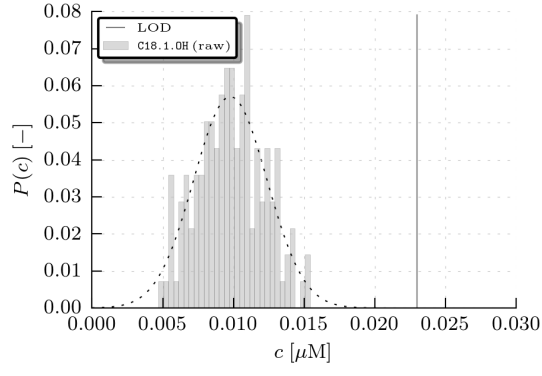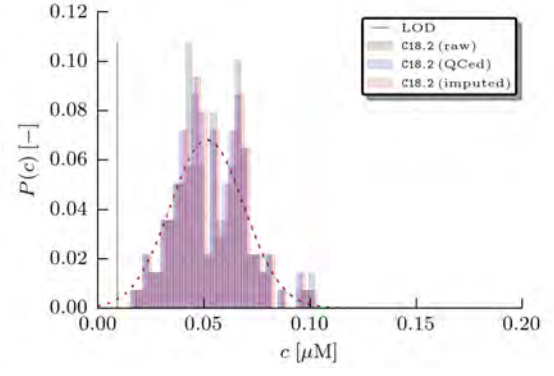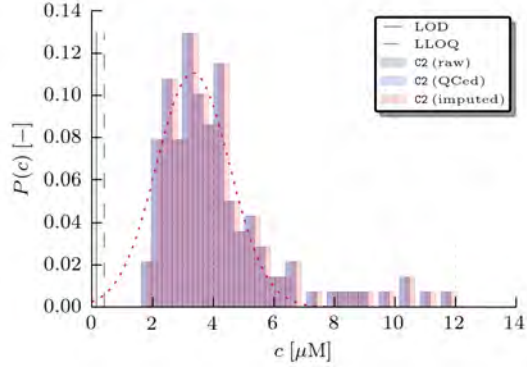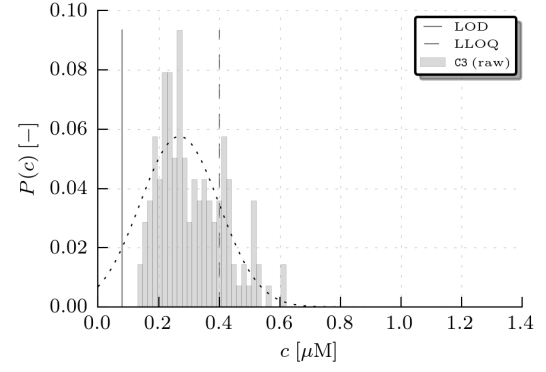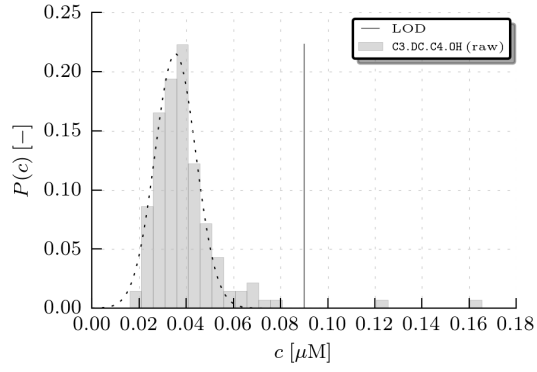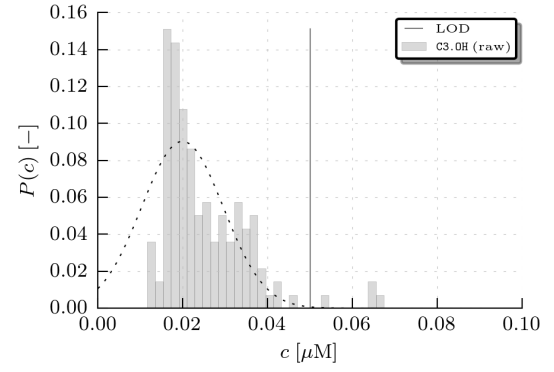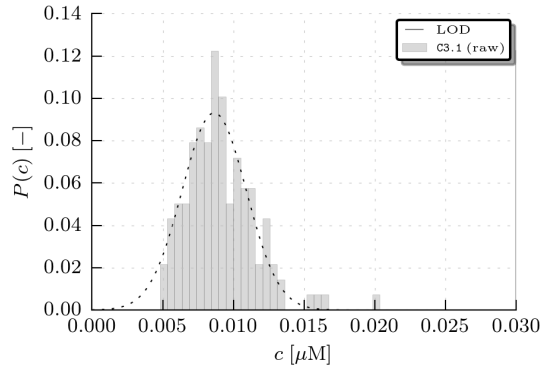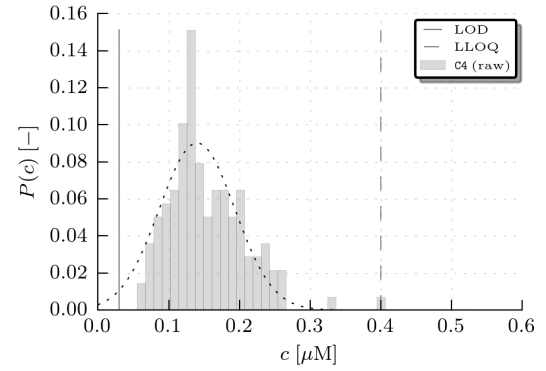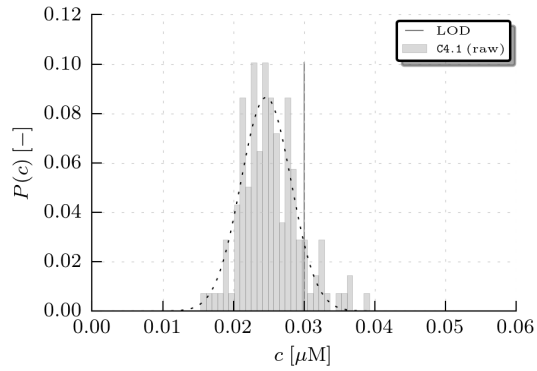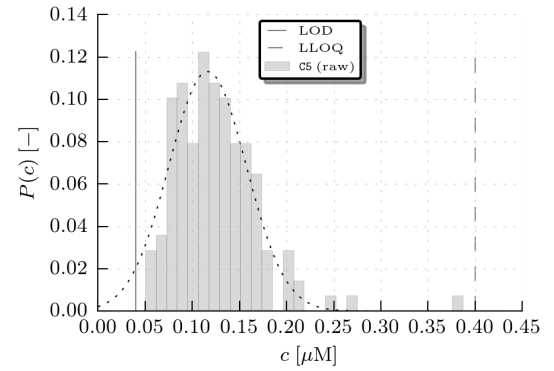

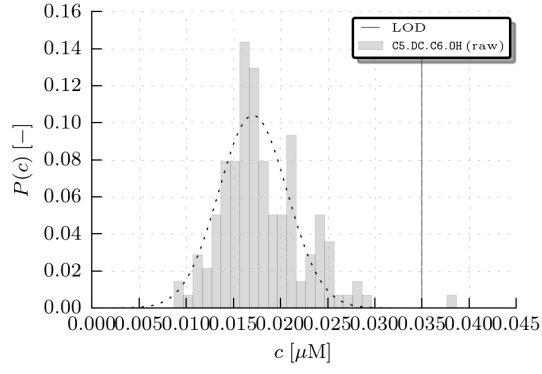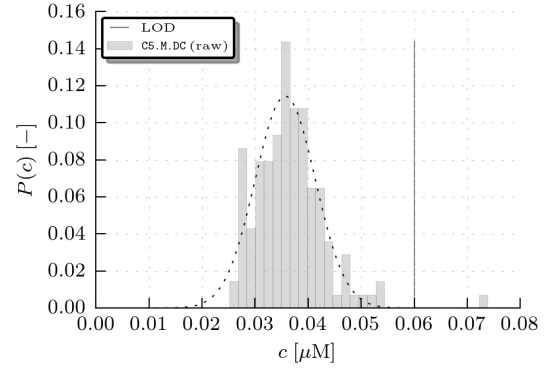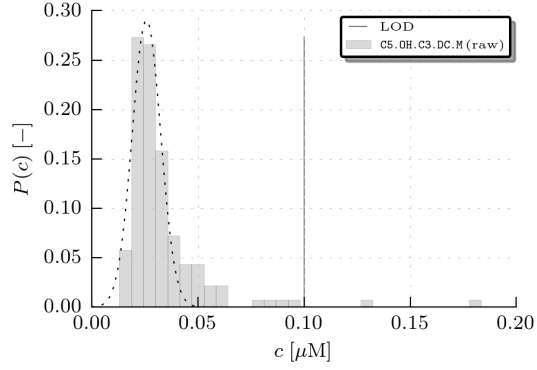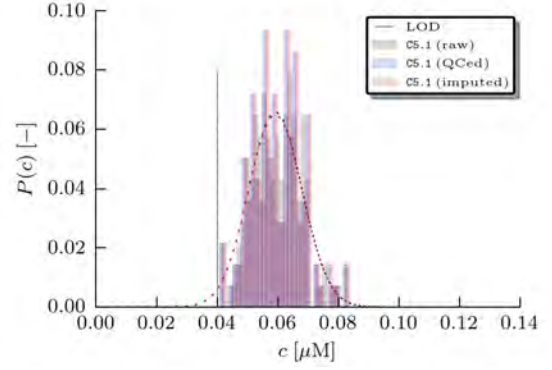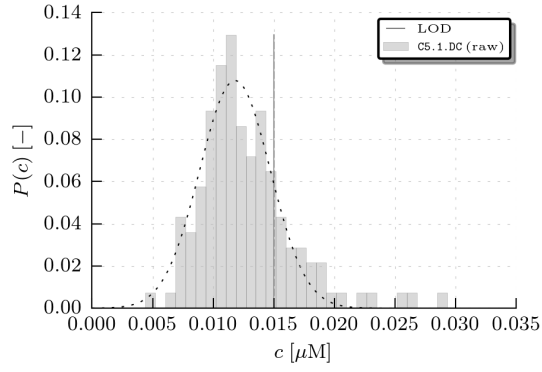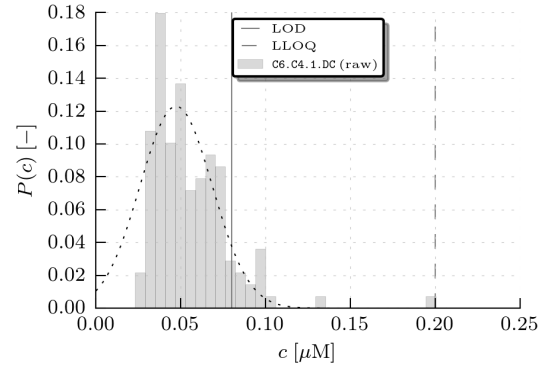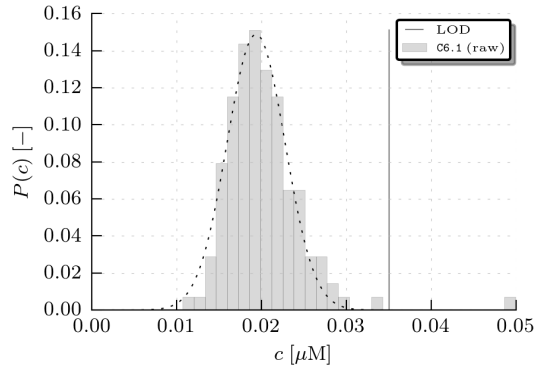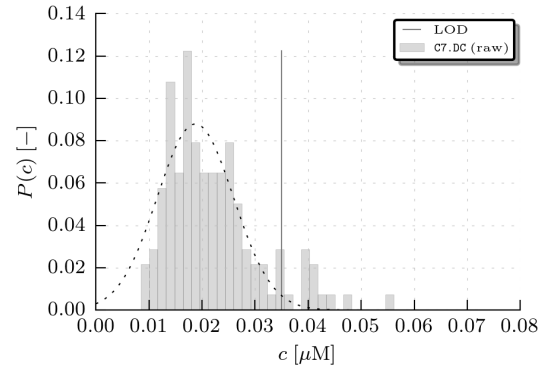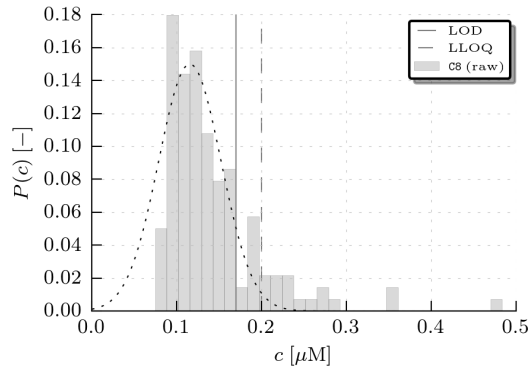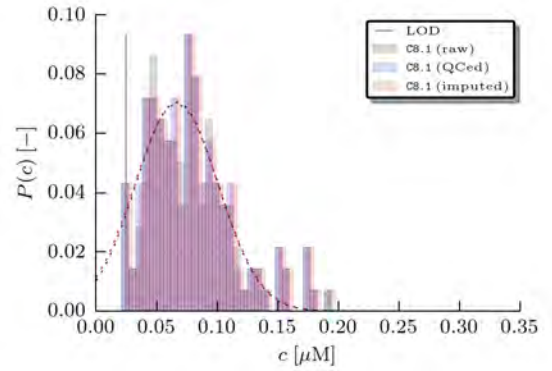

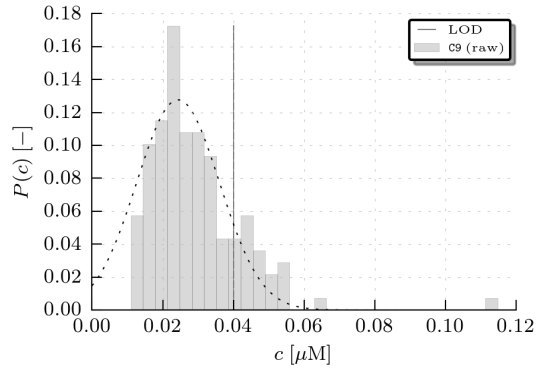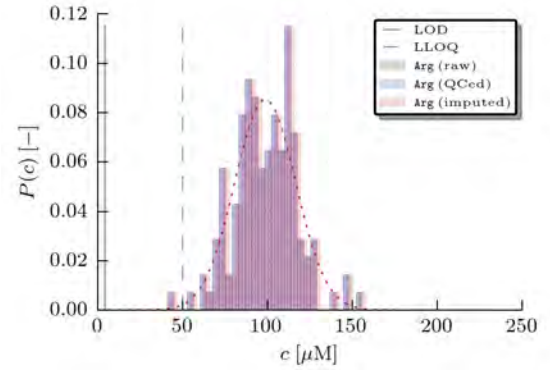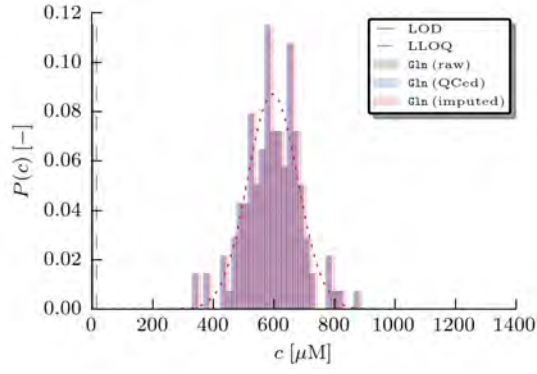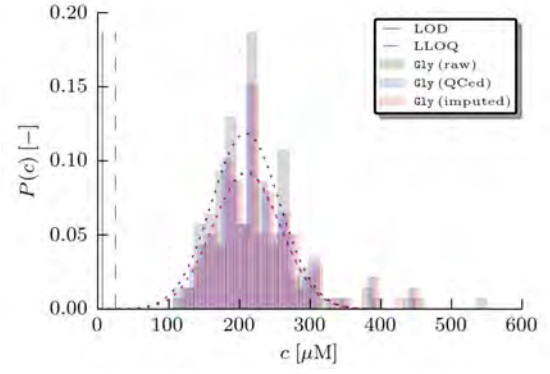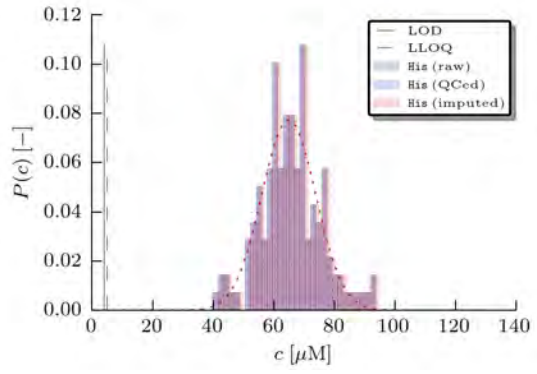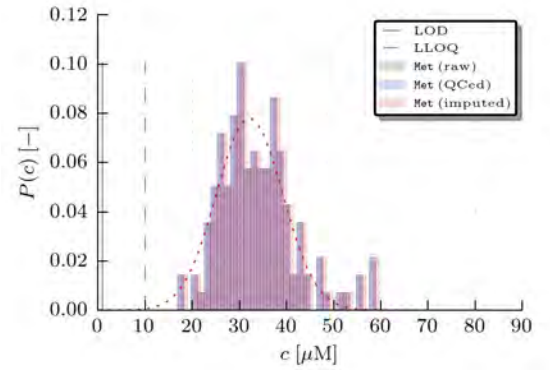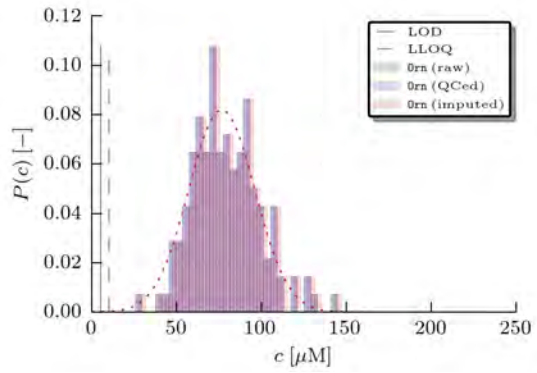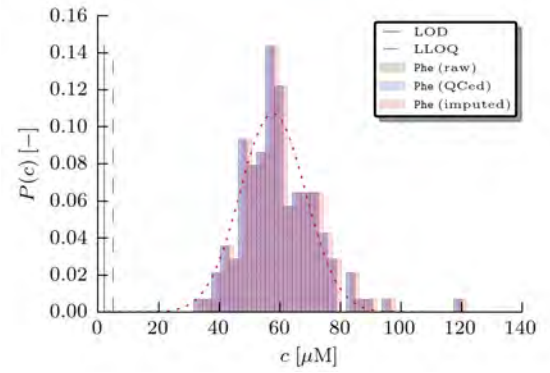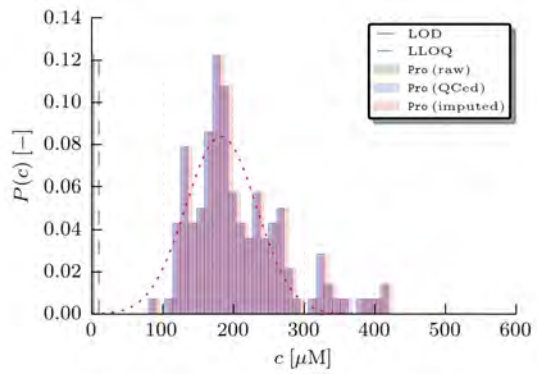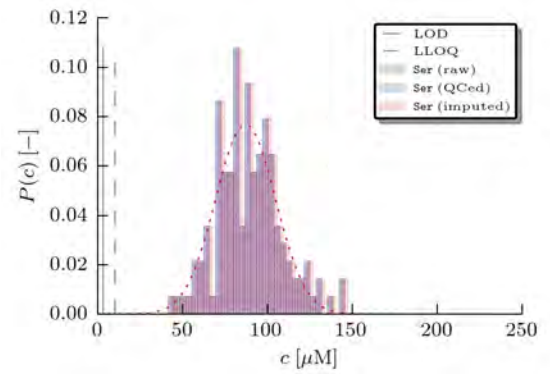

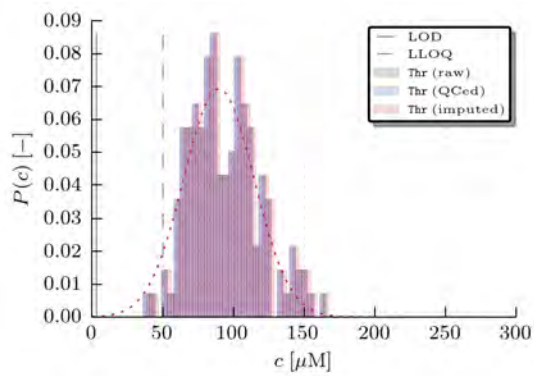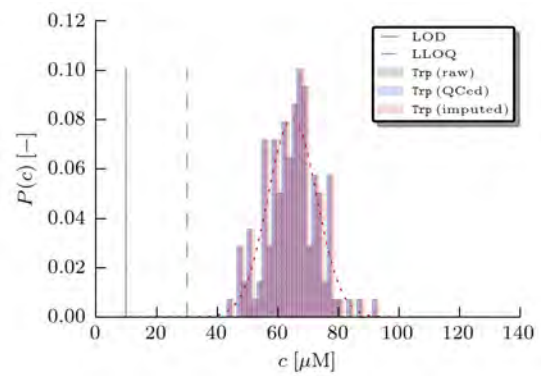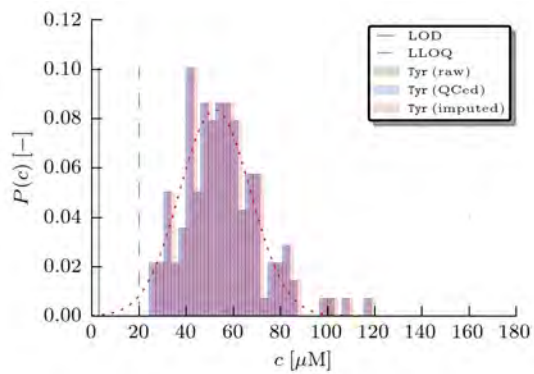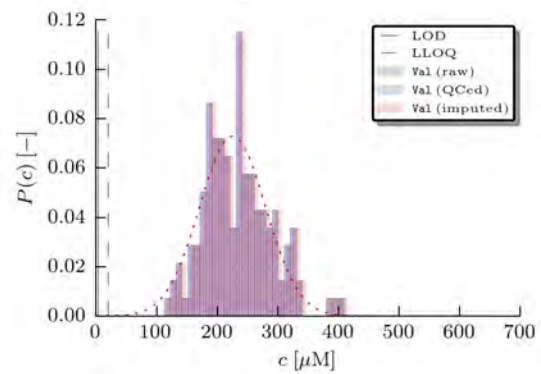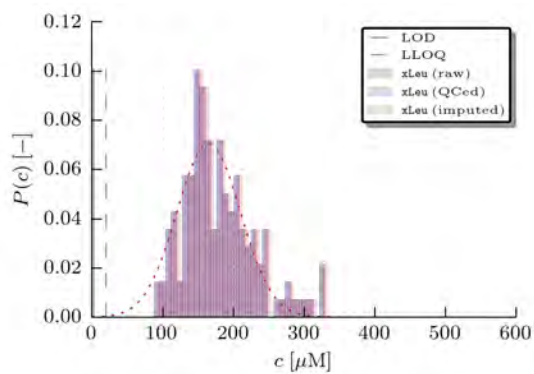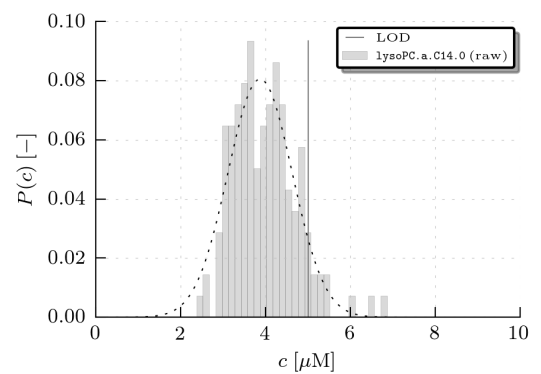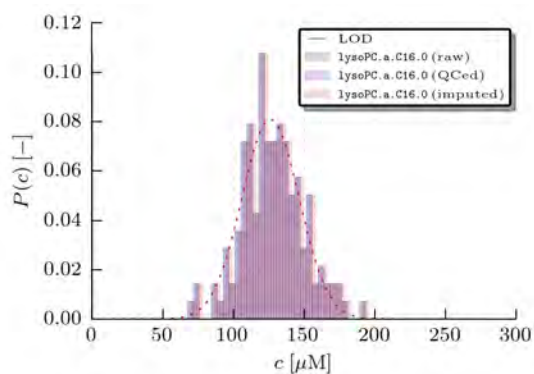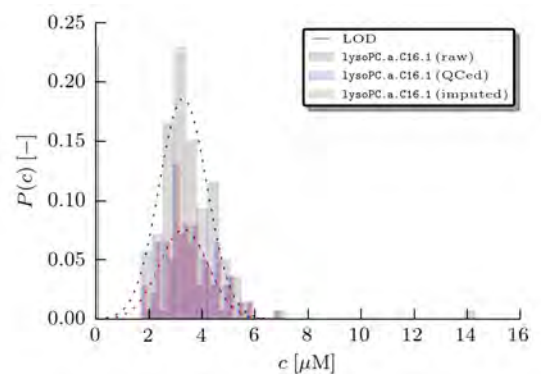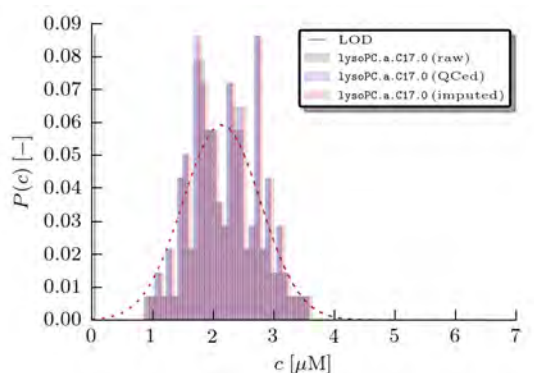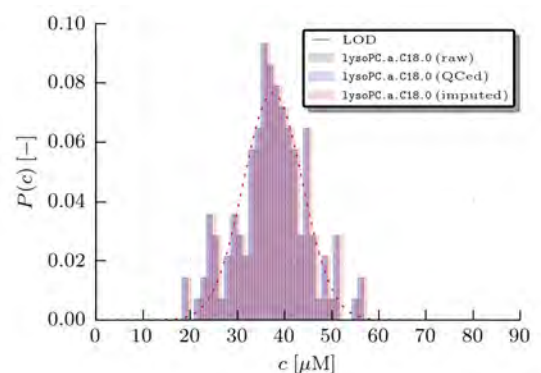

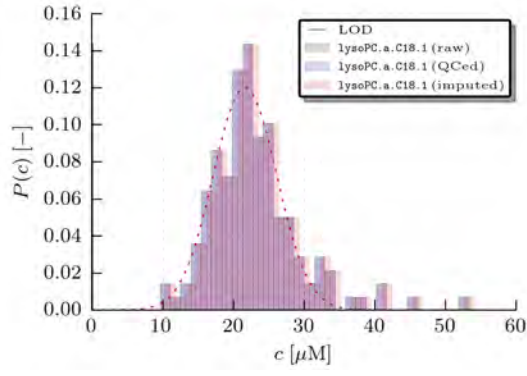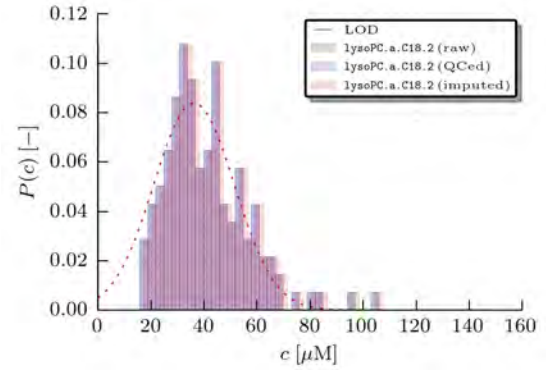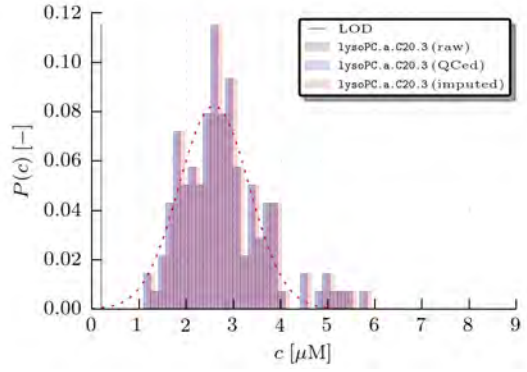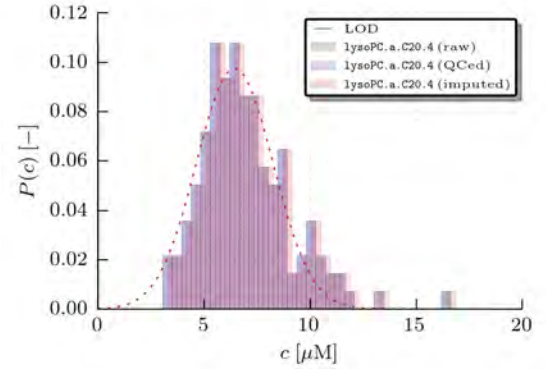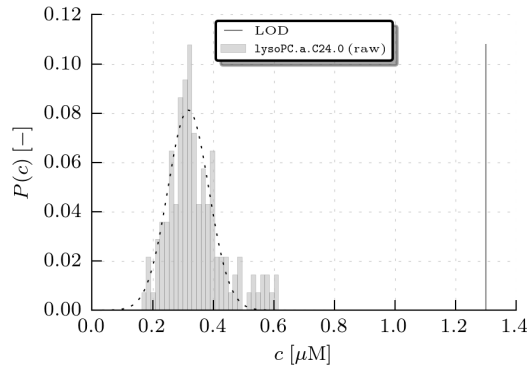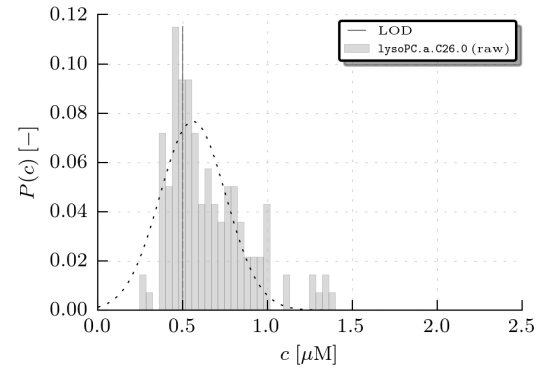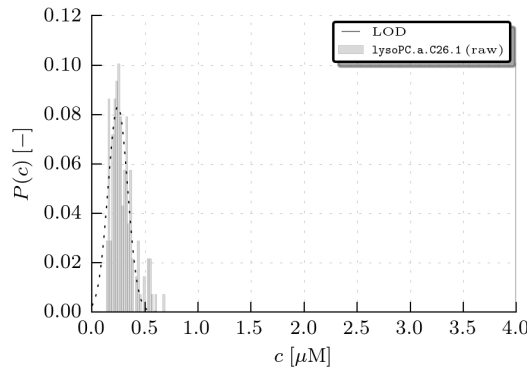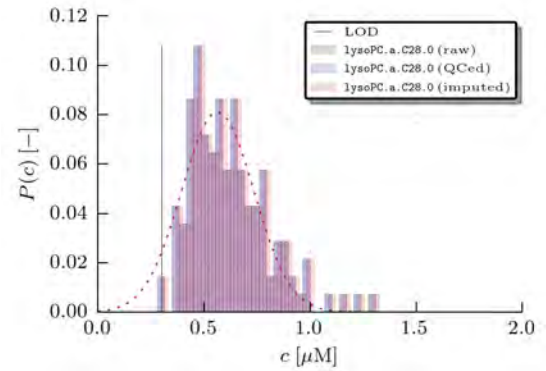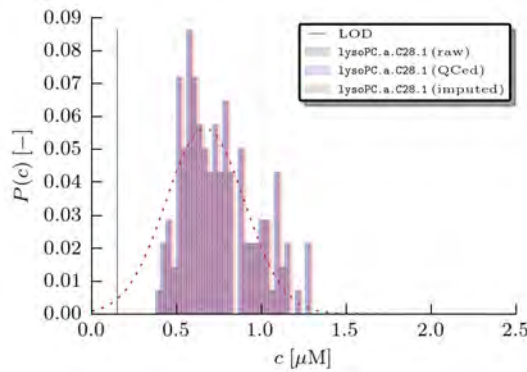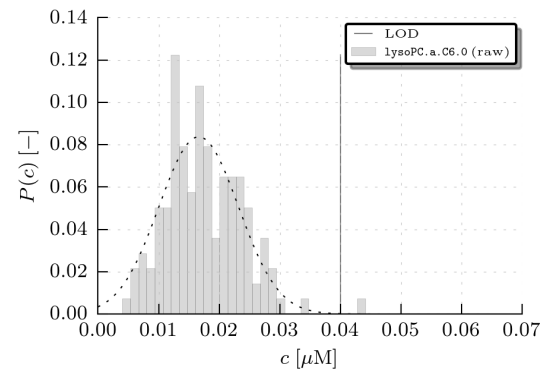

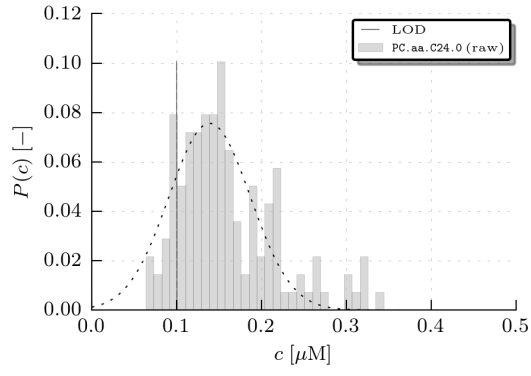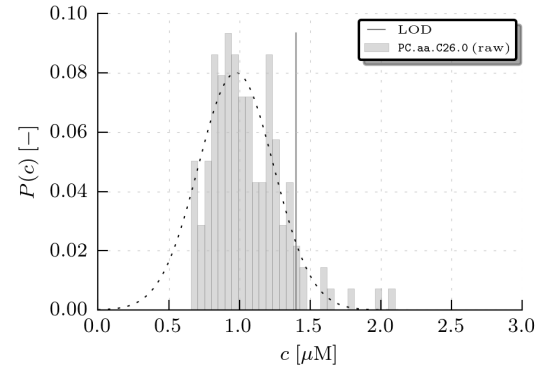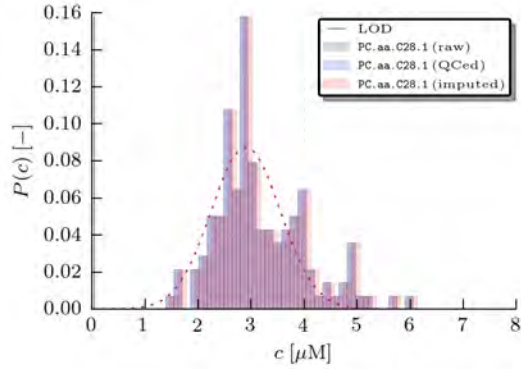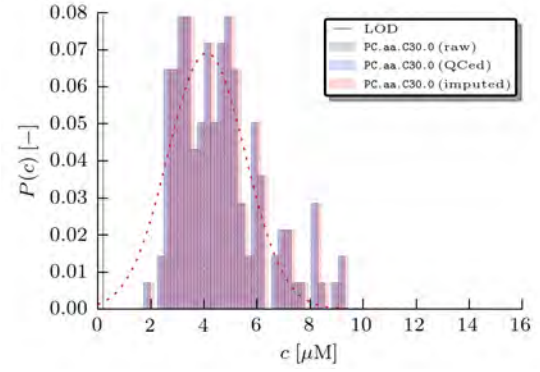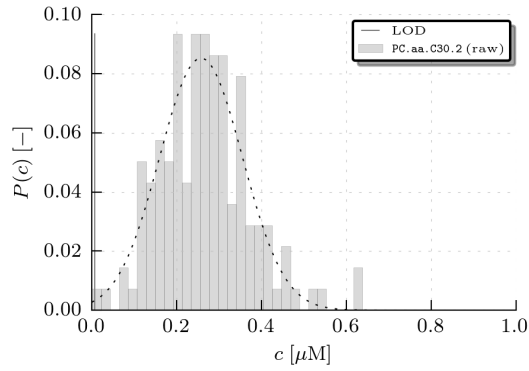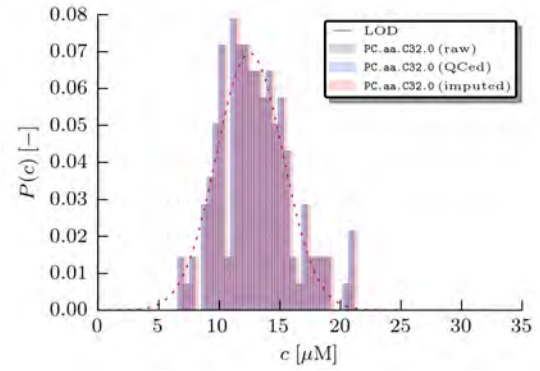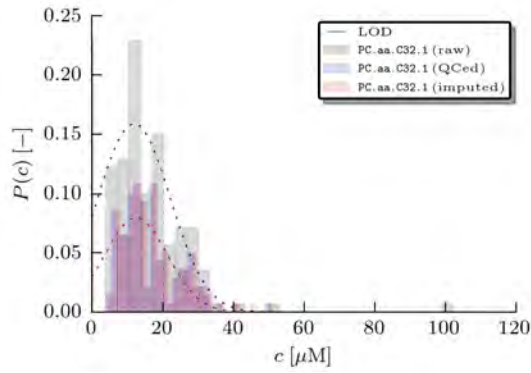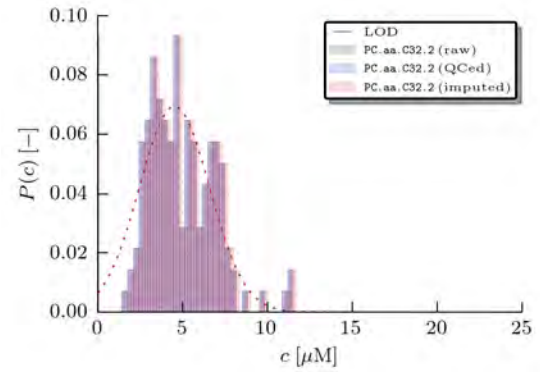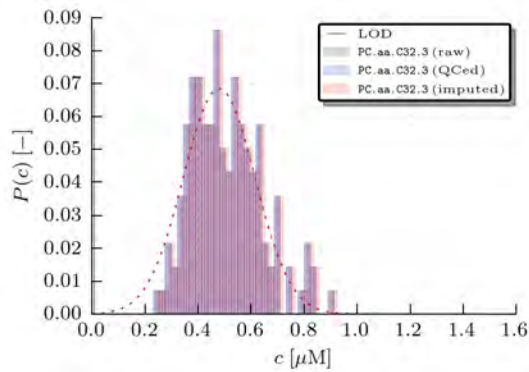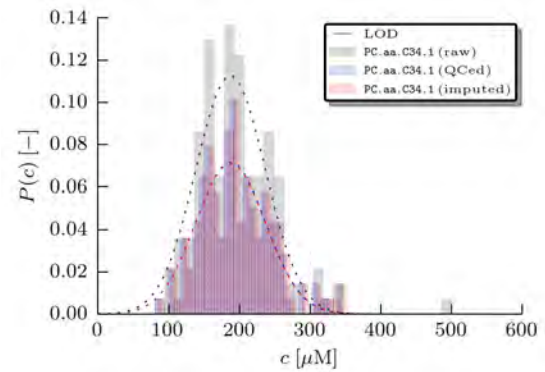

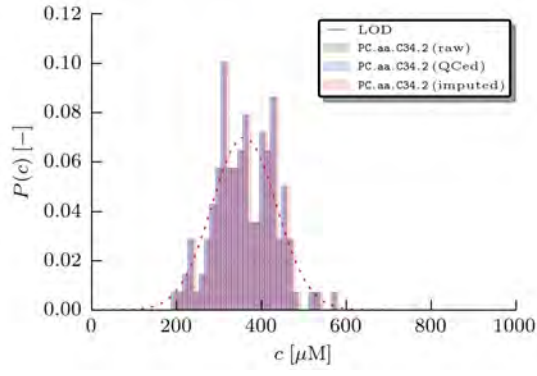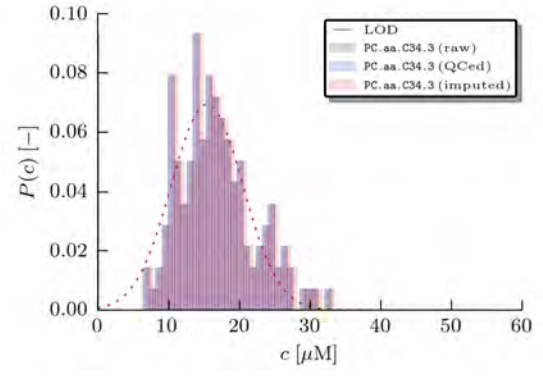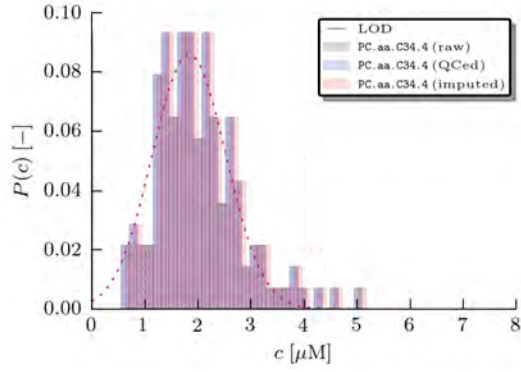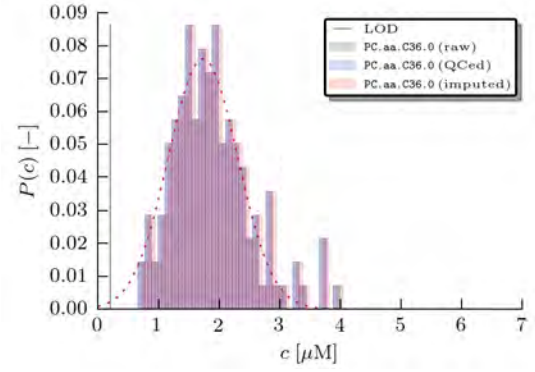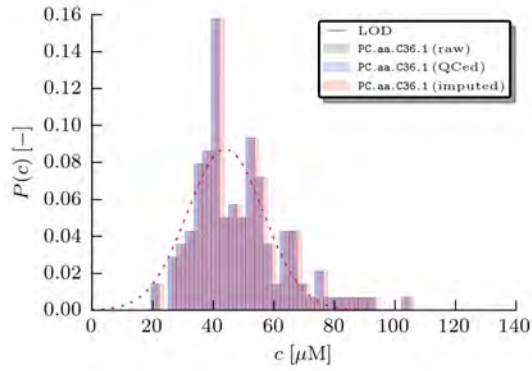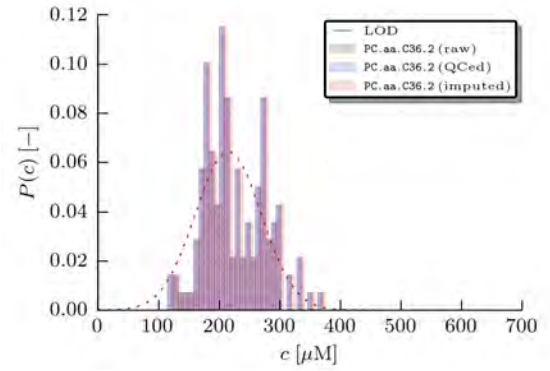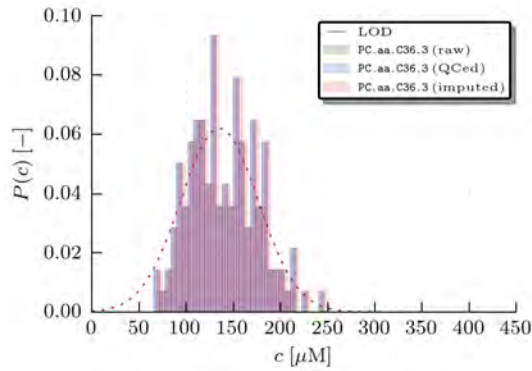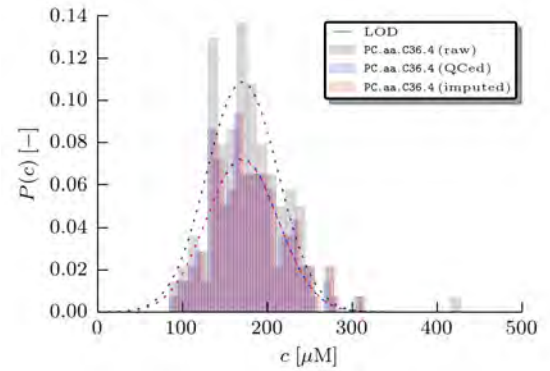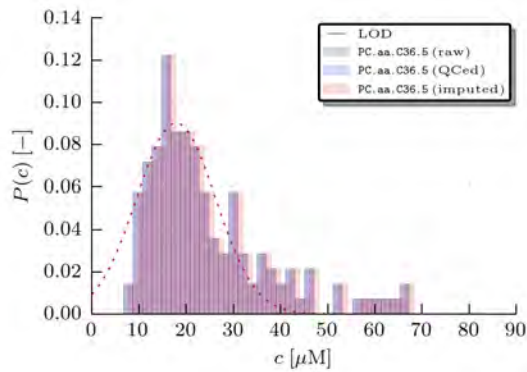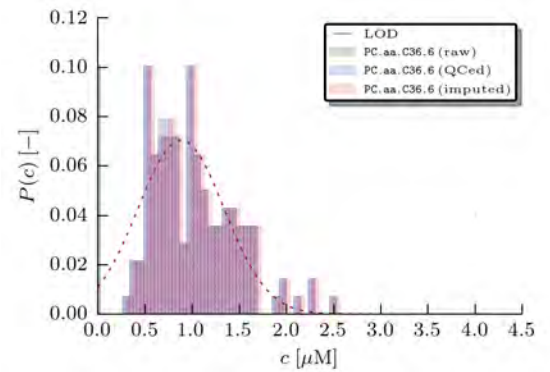

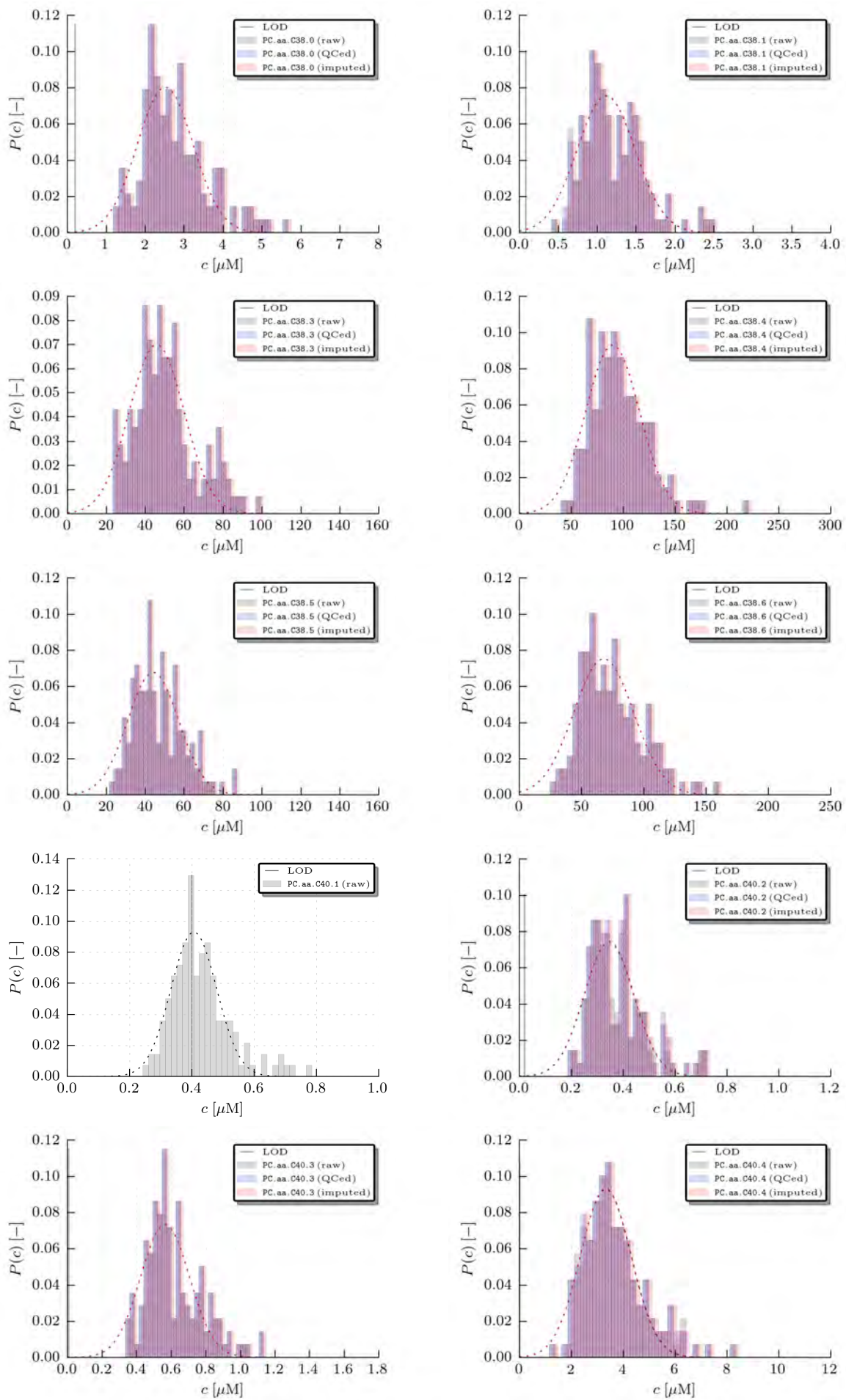

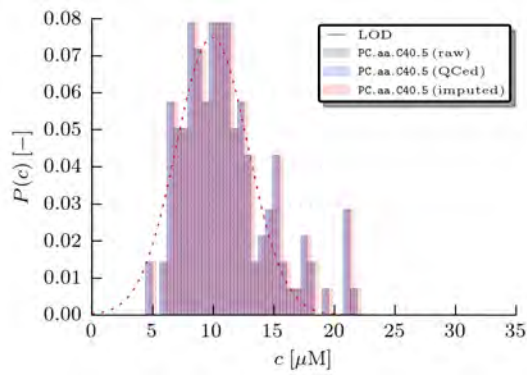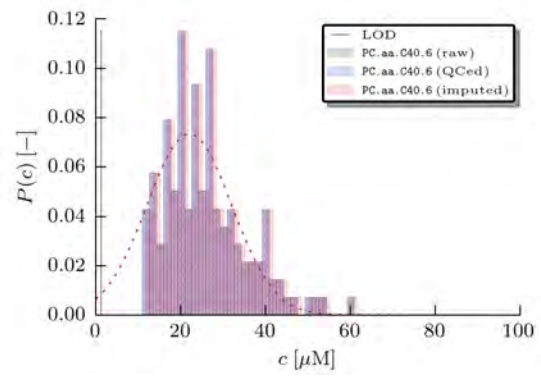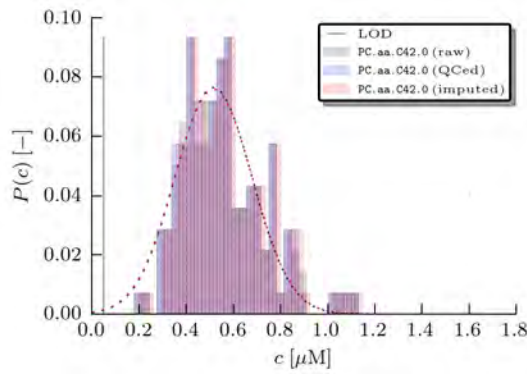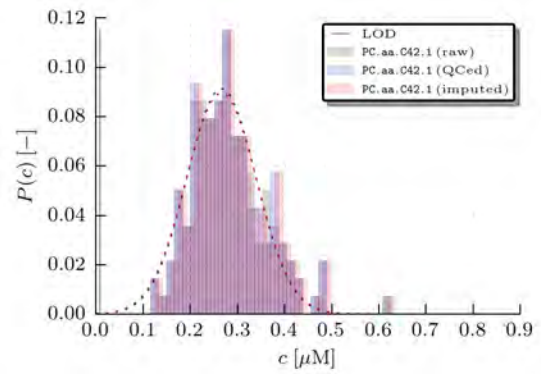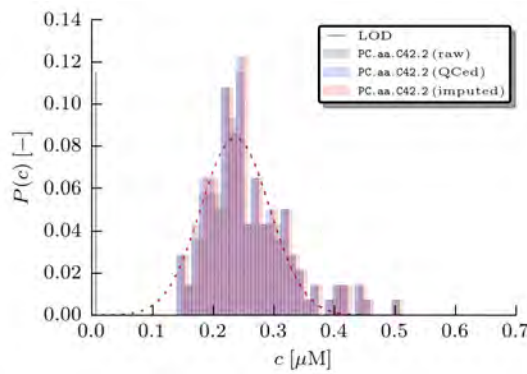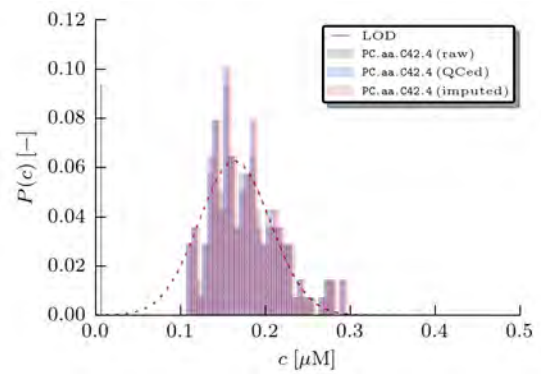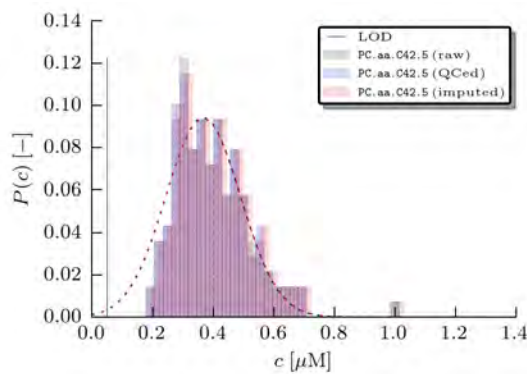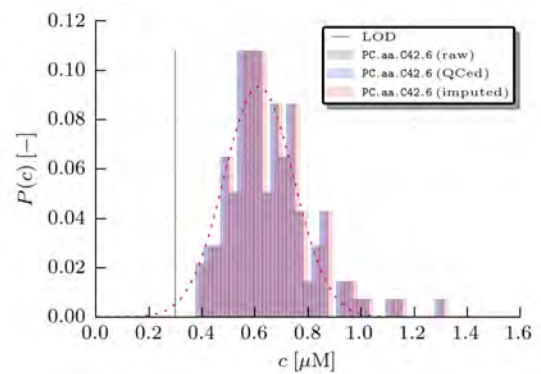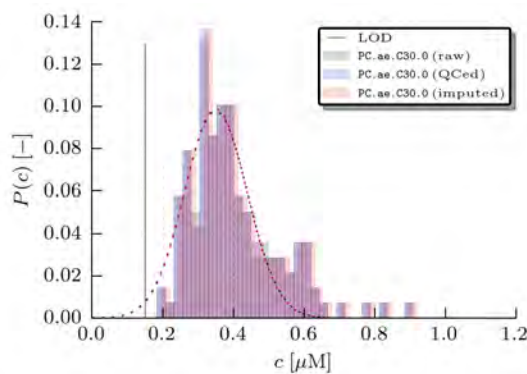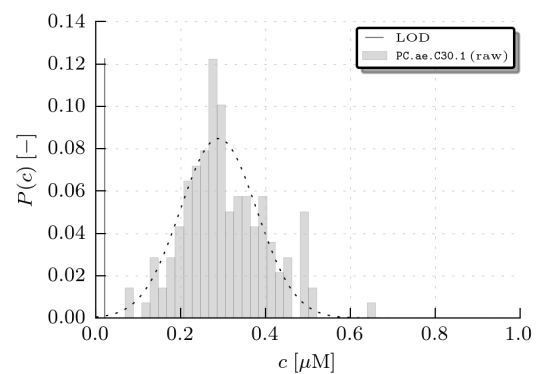

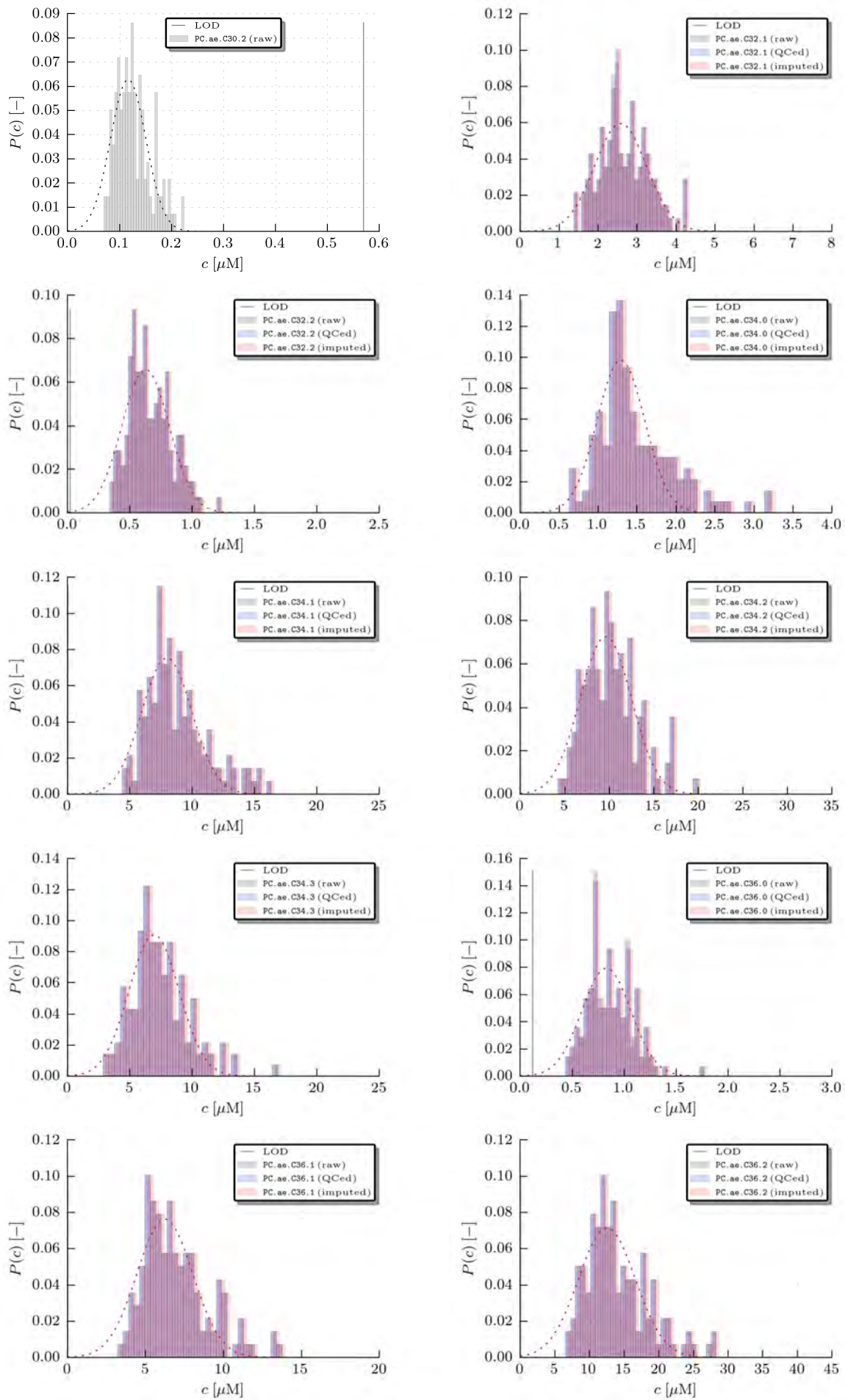

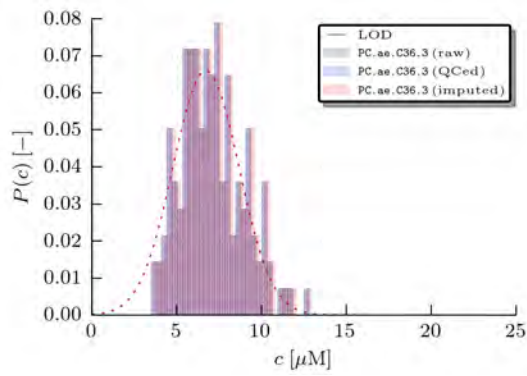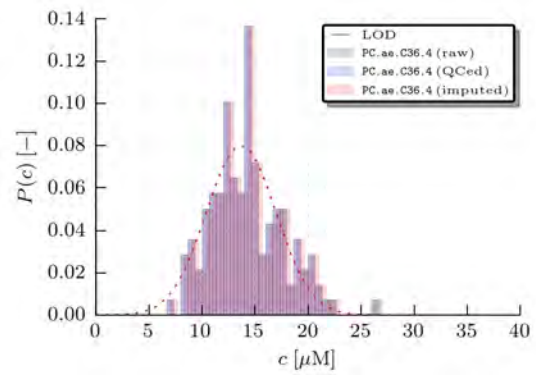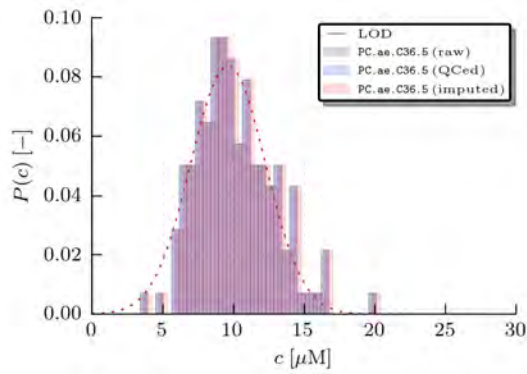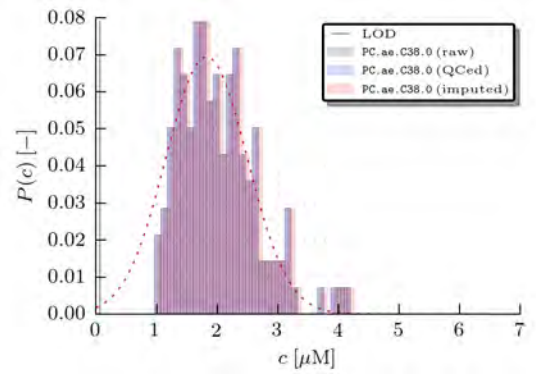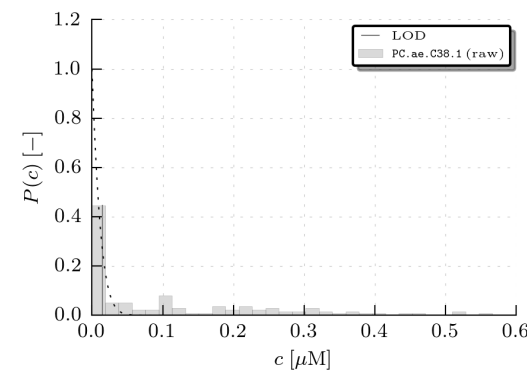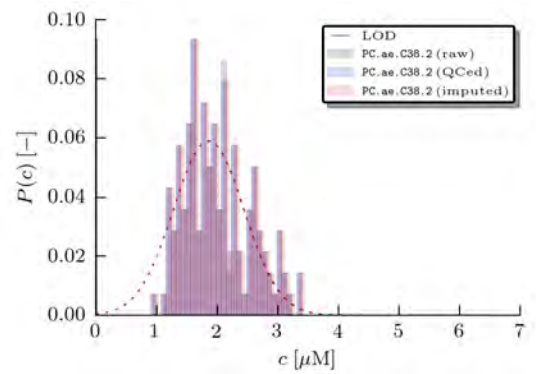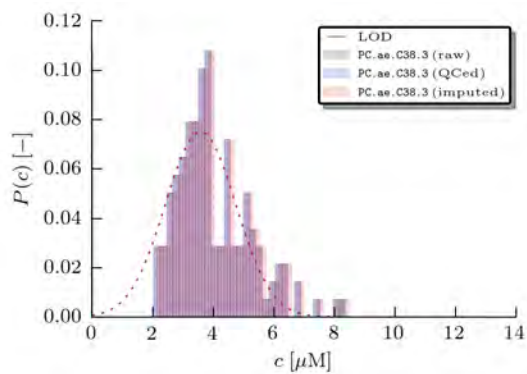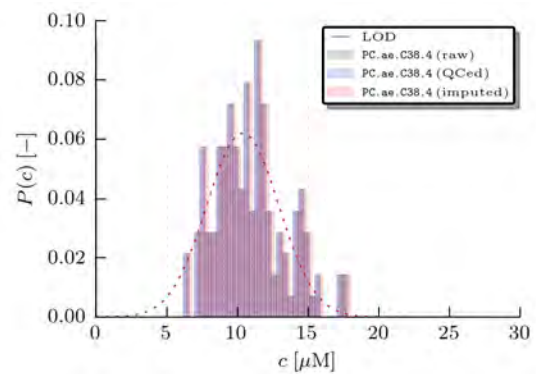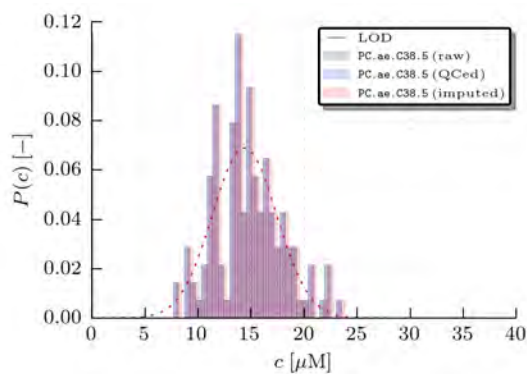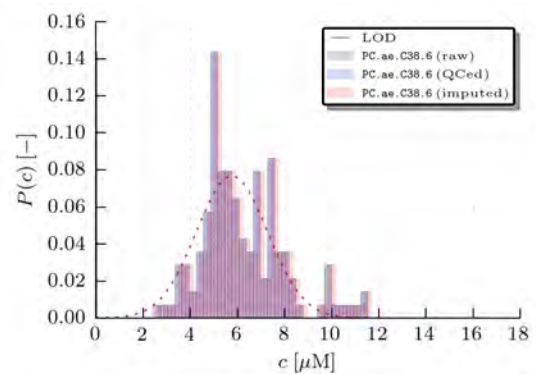

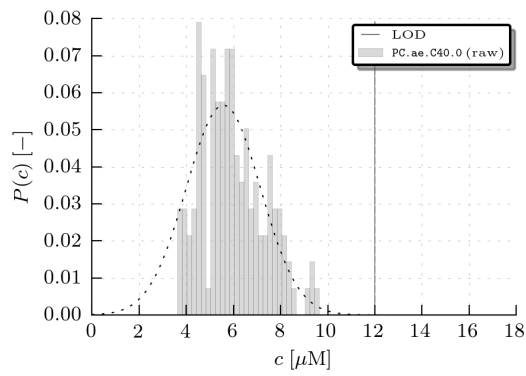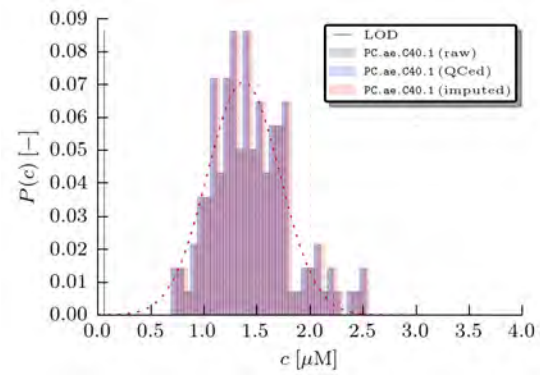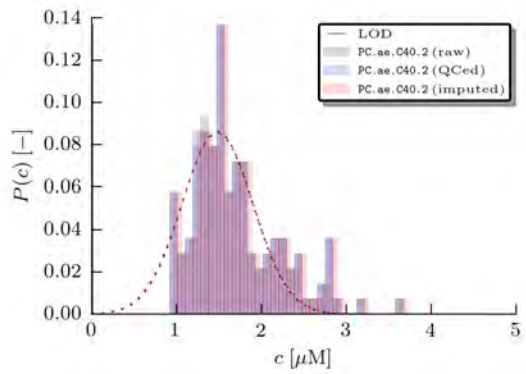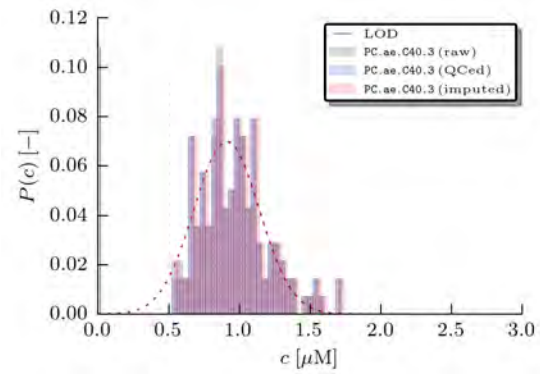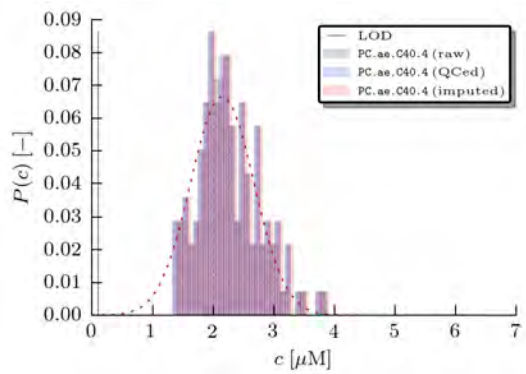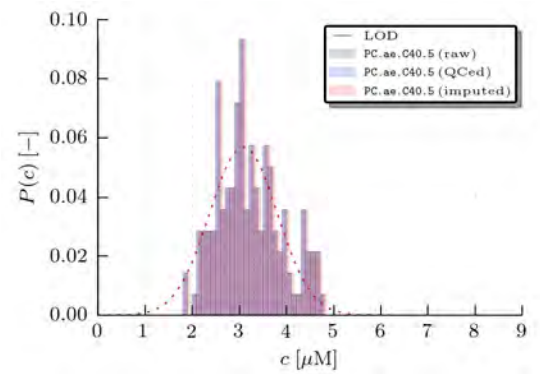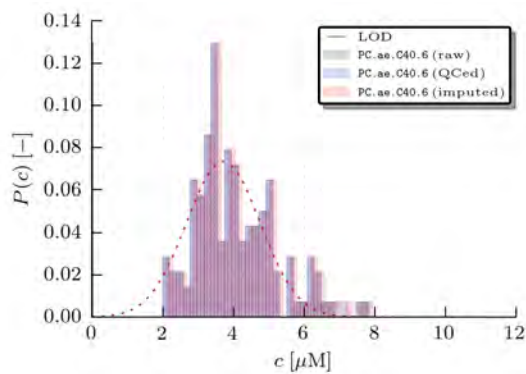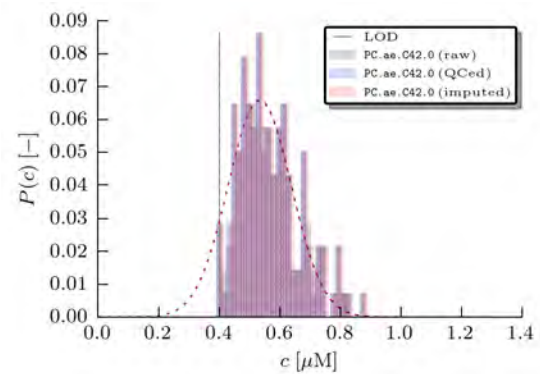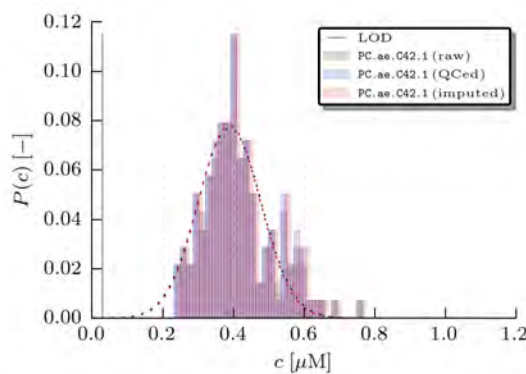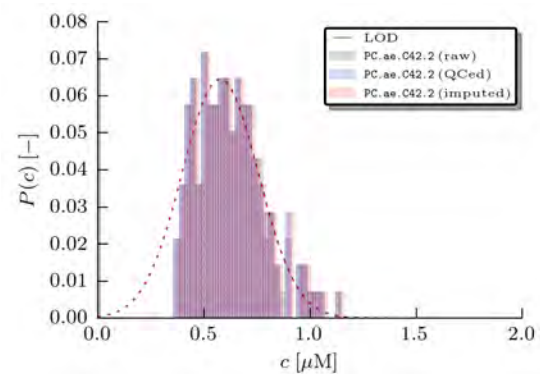

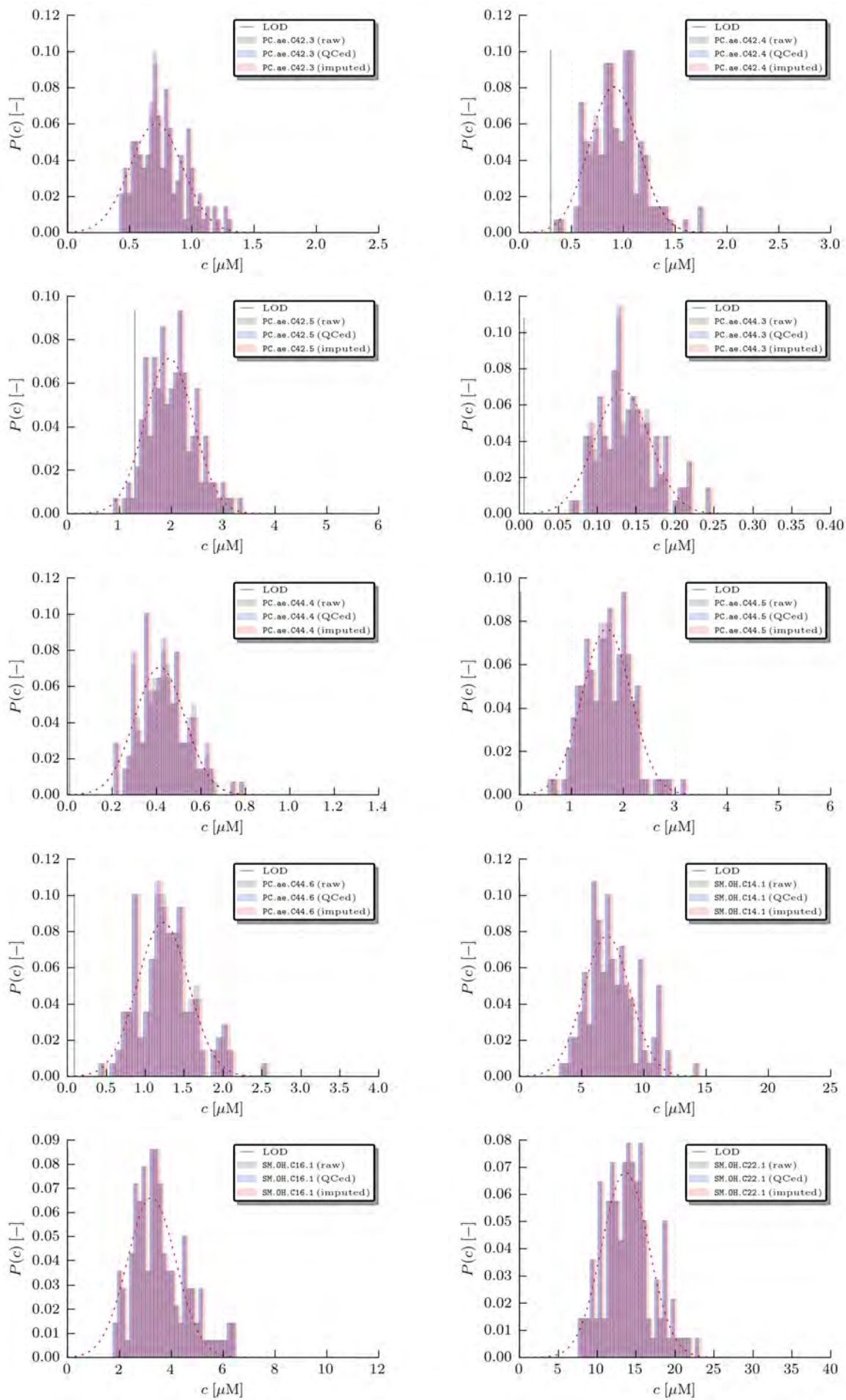

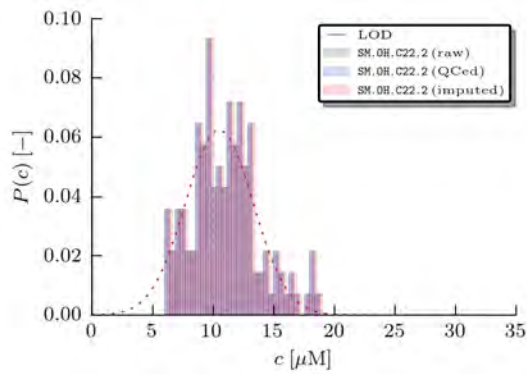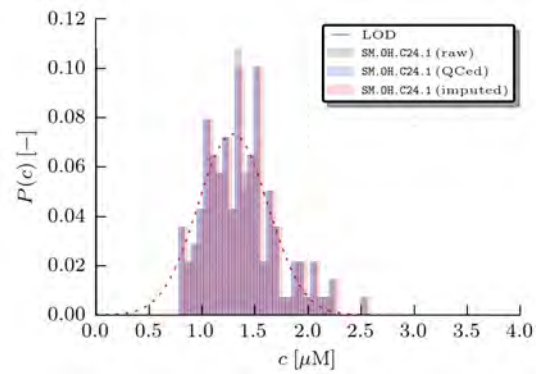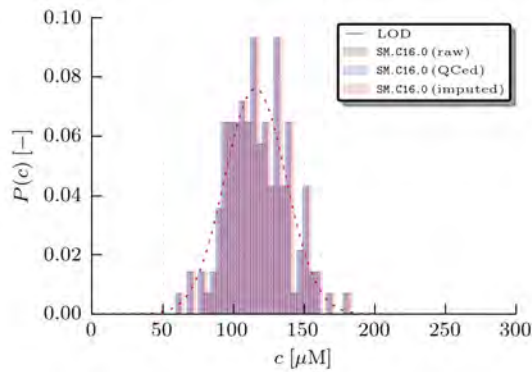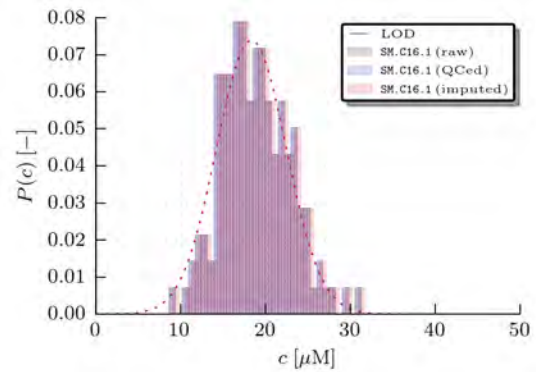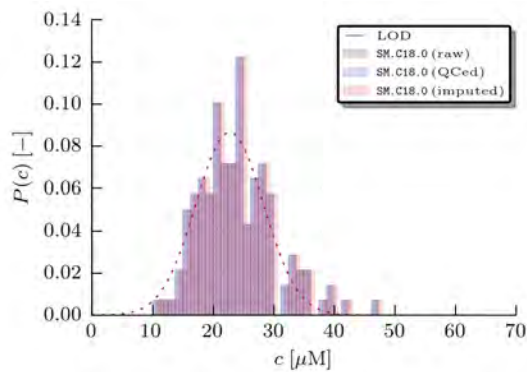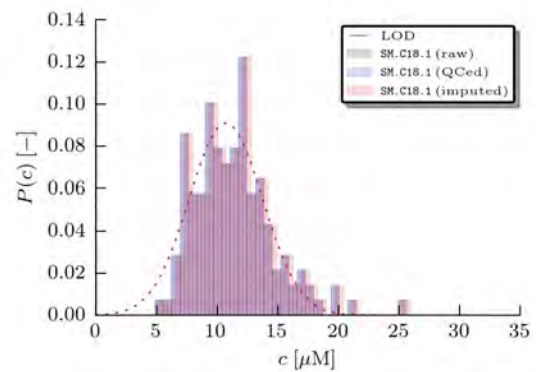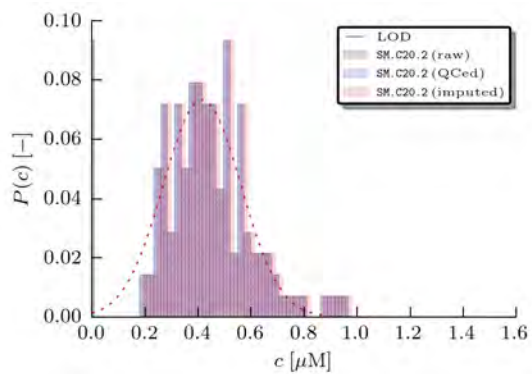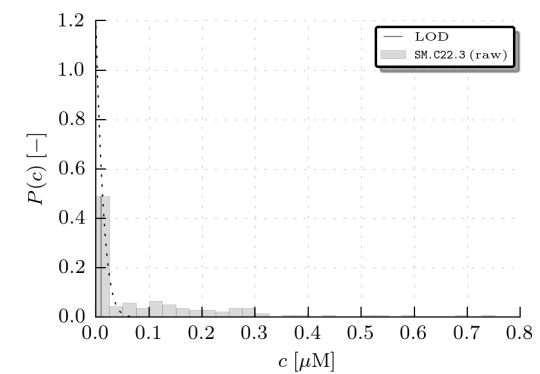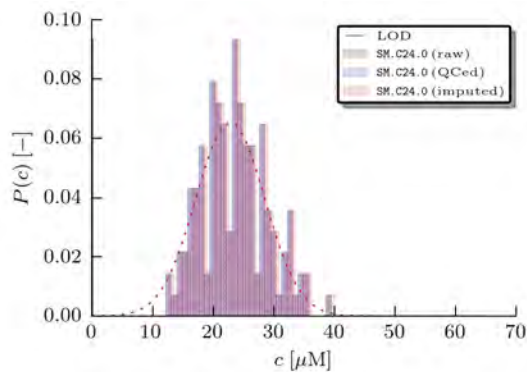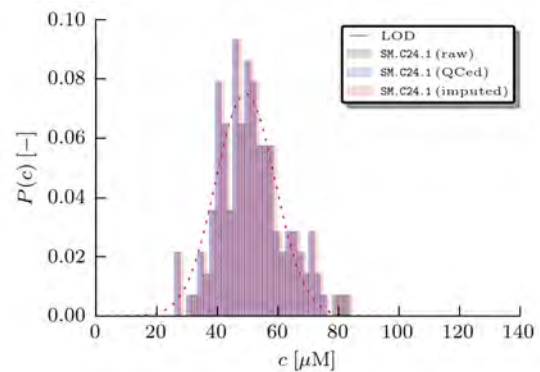

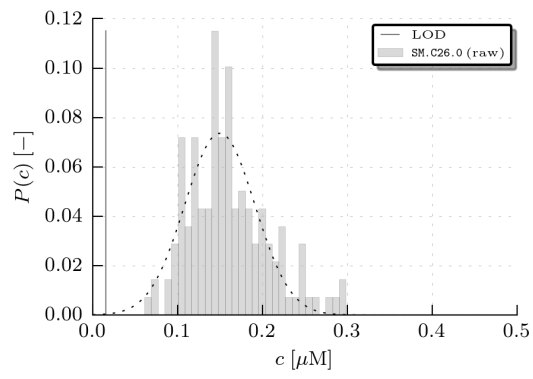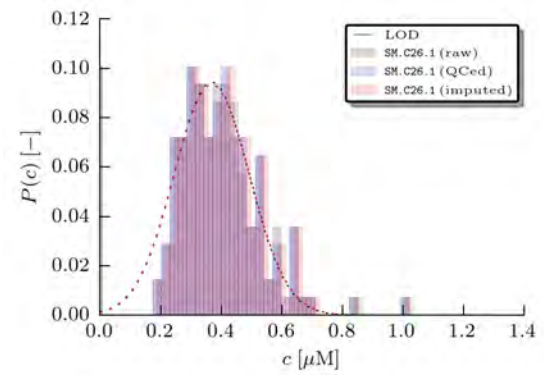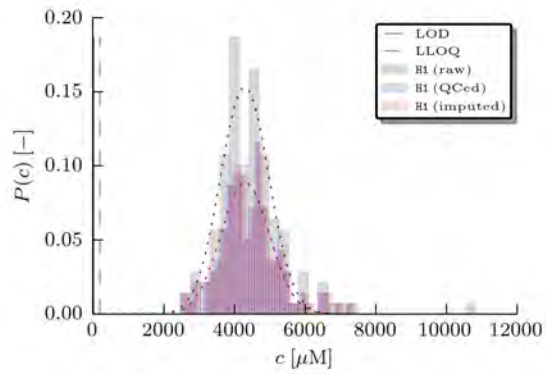

Supplement: Supplementary file 3 — Supplementary material 3 (PDF 2398 kb) [file 11306_2016_1084_MOESM3_ESM.pdf]
