# Supplementary material for: Integration of targeted metabolomics and transcriptomics identifies deregulation of phosphatidylcholine metabolism in Huntington’s disease peripheral blood samples
Source: Metabolomics. 2016 Jul 27;12:137. doi: 10.1007/s11306-016-1084-8 (PMC4963448; doi:10.1007/s11306-016-1084-8)
Supplement: Supplementary file 4 — Supplementary material 4 (PDF 392 kb) [file 11306_2016_1084_MOESM4_ESM.pdf]

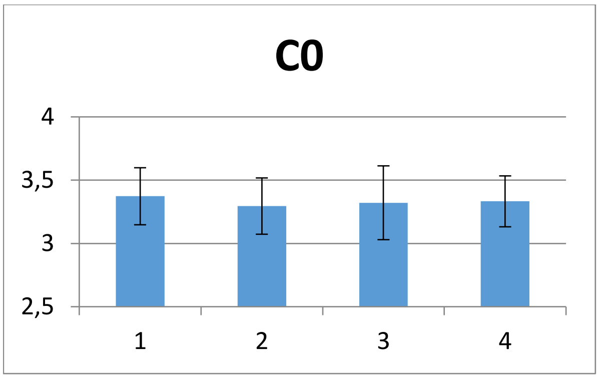
C0
4
3,5
3
2,5
1
2
3
4

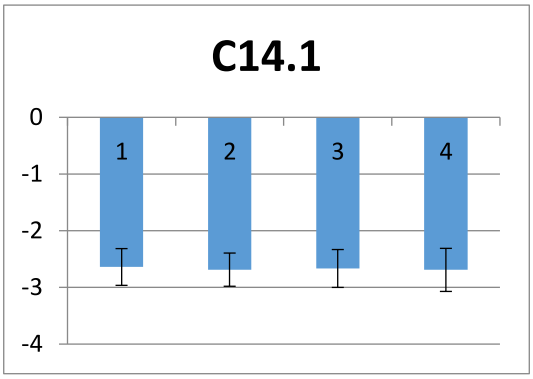
C14.1
0
-1
-2
-3
-4
1
2
3
4

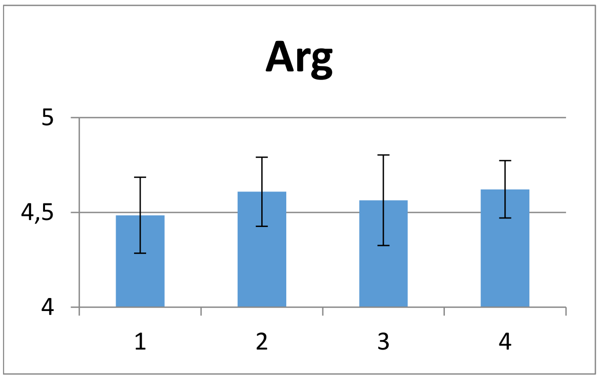
Arg
5
4,5
4
1
2
3
4

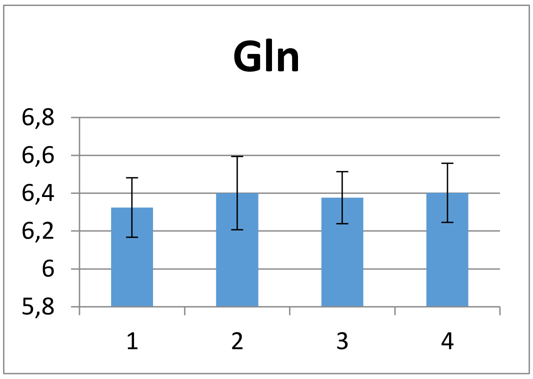
Gln
6,8
6,6
6,4
6,2
6
5,8
1
2
3
4

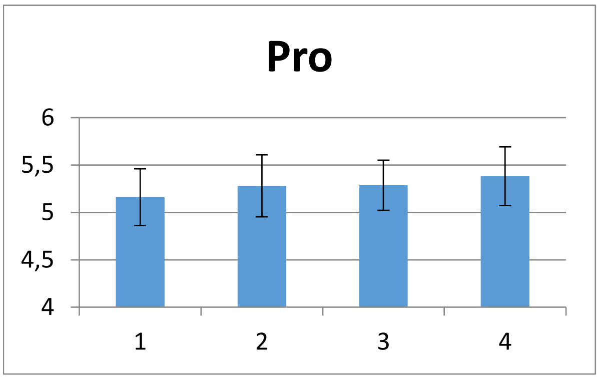
Pro
6
5,5
5
4,5
4
1
2
3
4

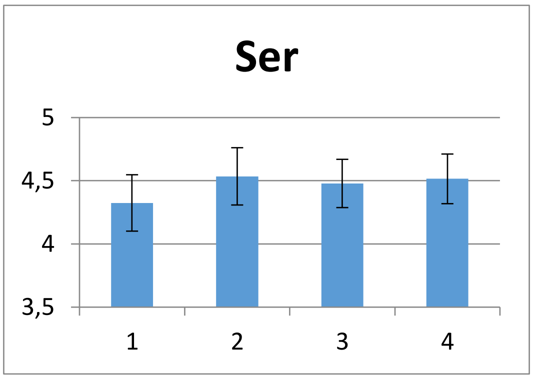
Ser
5
4,5
4
3,5
1
2
3
4

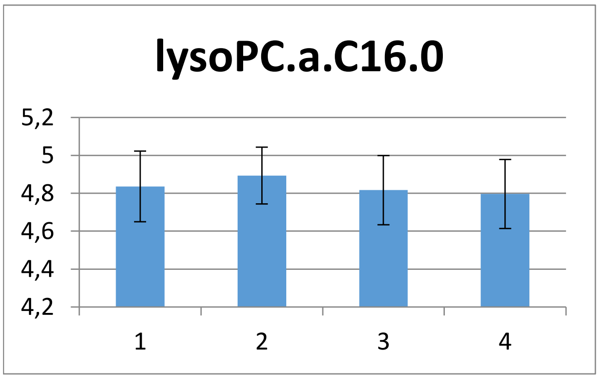
lysoPC.a.C16.0
5,2
5
4,8
4,6
4,4
4,2
1
2
3
4

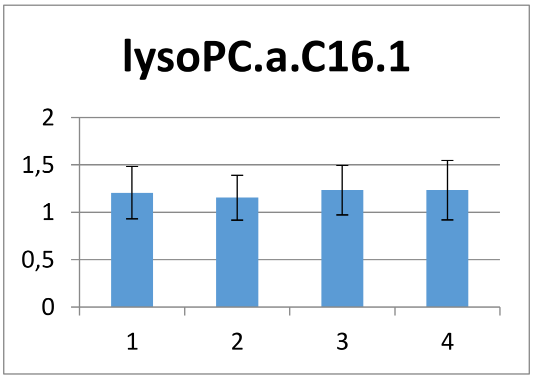
lysoPC.a.C16.1
2
1,5
1
0,5
0
1
2
3
4

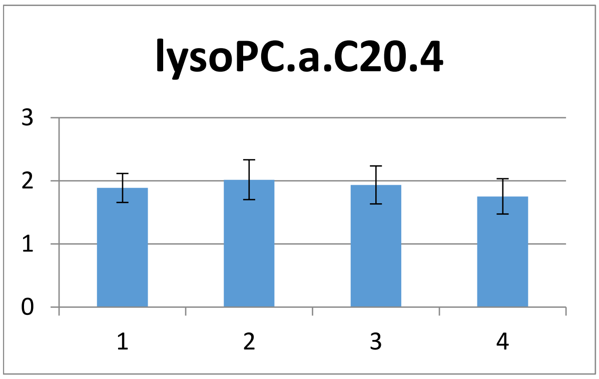
lysoPC.a.C20.4
3
2
1
0
1
2
3
4

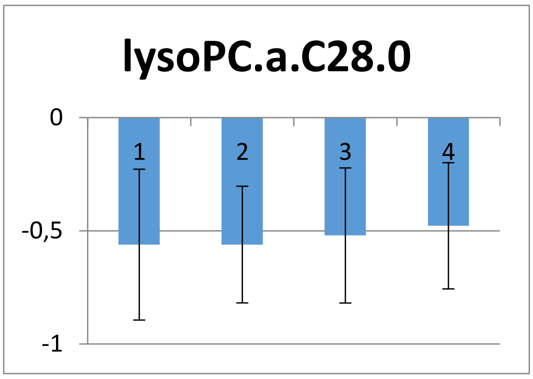
lysoPC.a.C28.0
0
-0,5
-1
1
2
3
4

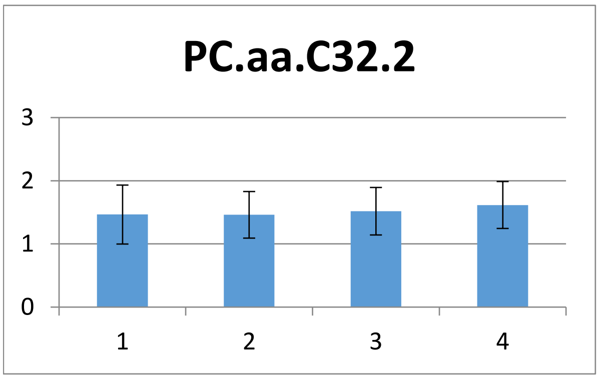
PC.aa.C32.2
3
2
1
0
1
2
3
4

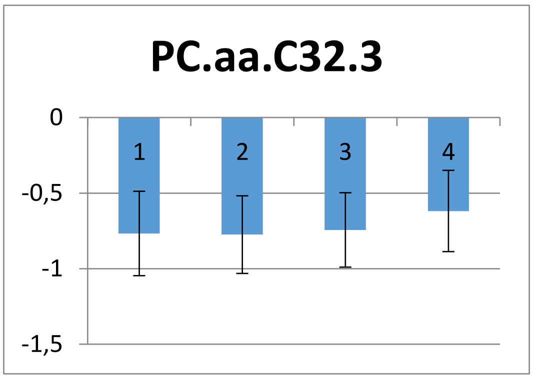
PC.aa.C32.3
0
-0,5
-1
-1,5
1
2
3
4

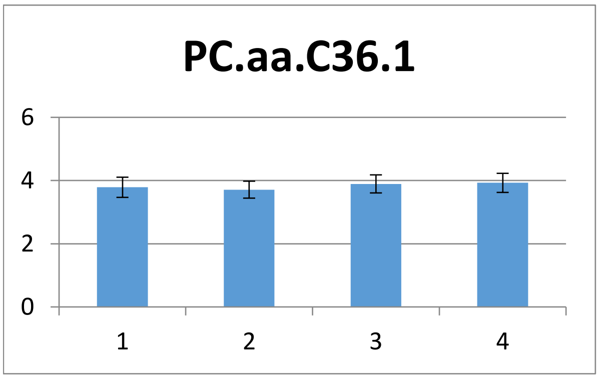
PC.aa.C36.1
6
4
2
0
1
2
3
4

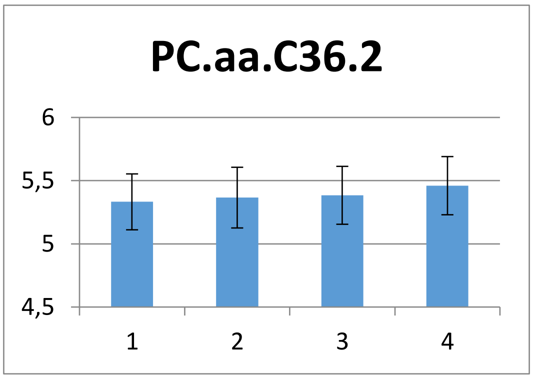
PC.aa.C36.2
6
5,5
5
4,5
1
2
3
4

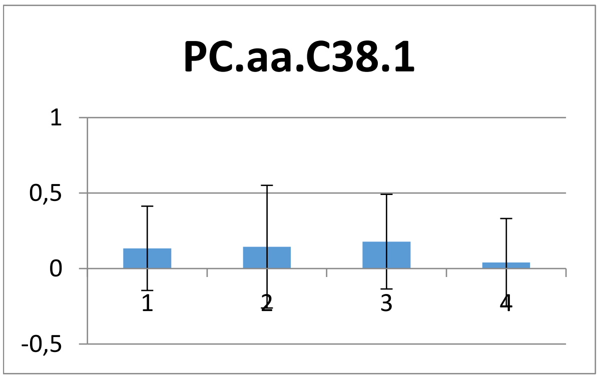
PC.aa.C38.1
1
0,5
0
-0,5
1
2
3
4

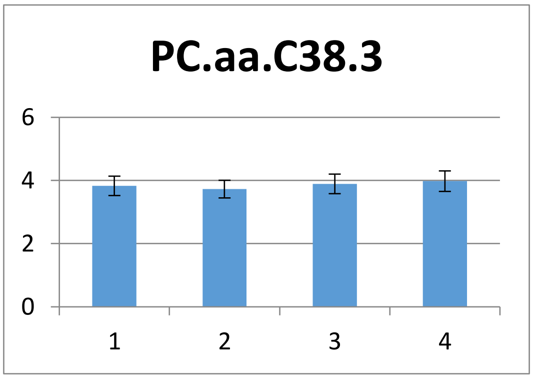
PC.aa.C38.3
6
4
2
0
1
2
3
4

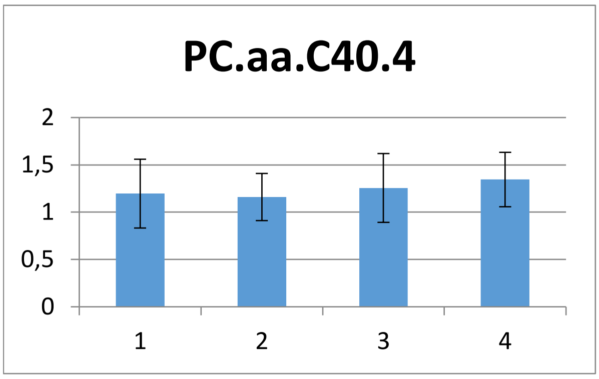
PC.aa.C40.4
2
1,5
1
0,5
0
1
2
3
4

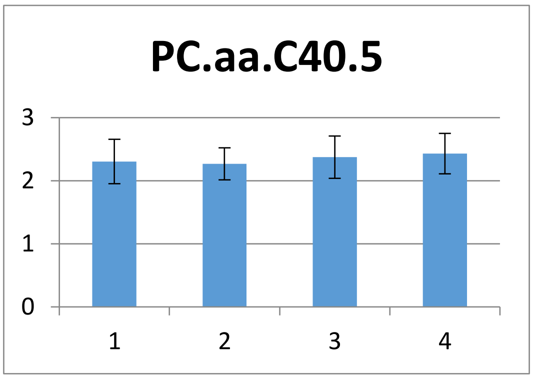
PC.aa.C40.5
3
2
1
0
1
2
3
4

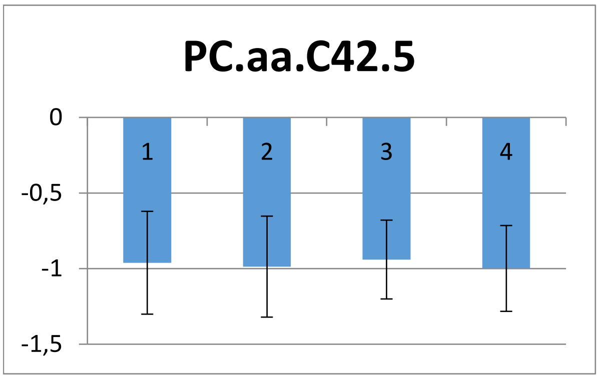
PC.aa.C42.5
0
-0,5
-1
-1,5
1
2
3
4

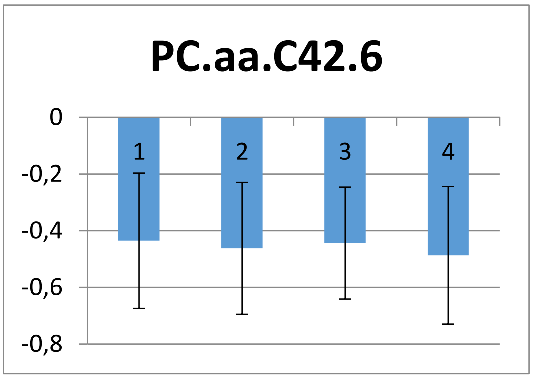
PC.aa.C42.6
0
-0,2
-0,4
-0,6
-0,8
1
2
3
4

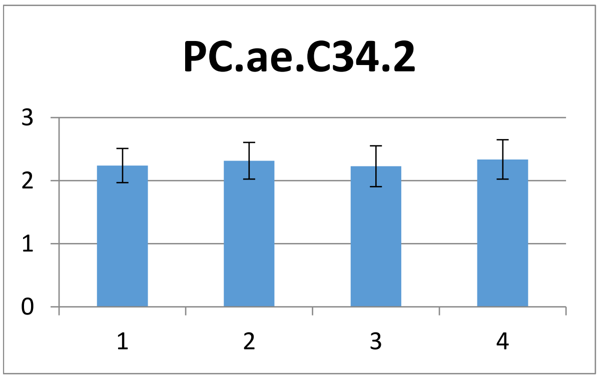
PC.ae.C34.2
3
2
1
0
1
2
3
4

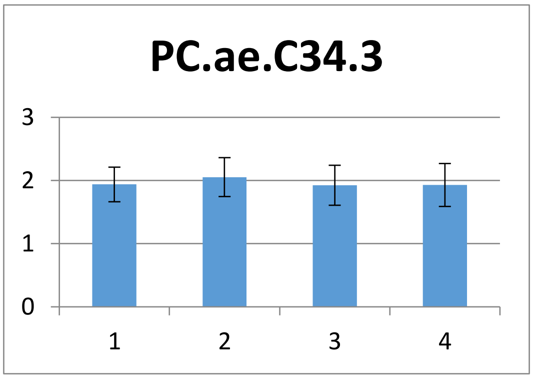
PC.ae.C34.3
3
2
1
0
1
2
3
4

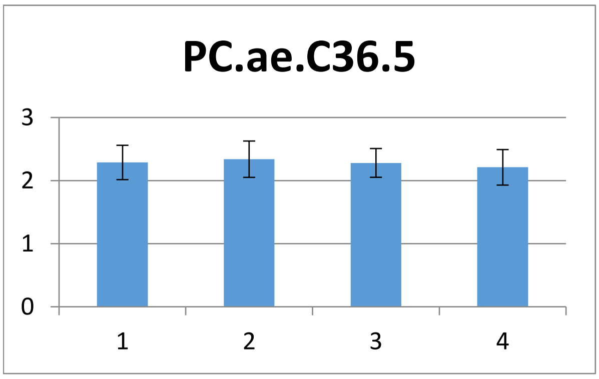
PC.ae.C36.5
3
2
1
0
1
2
3
4

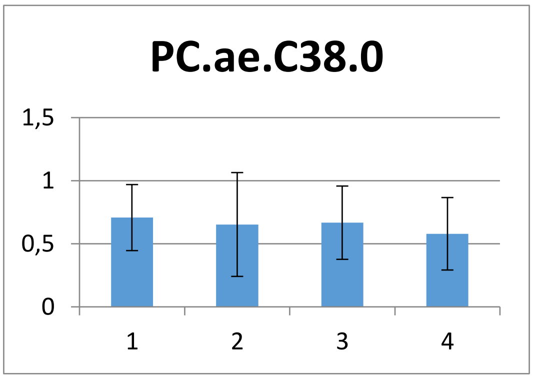
PC.ae.C38.0
1,5
1
0,5
0
1
2
3
4

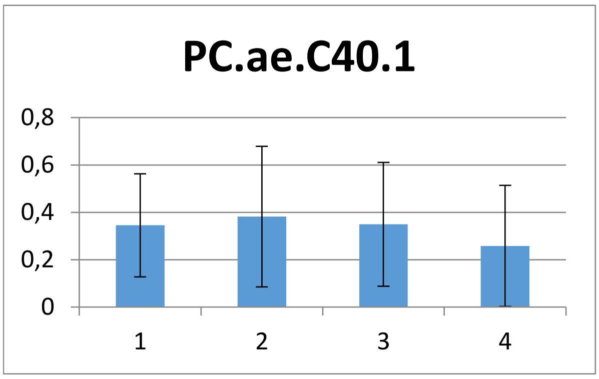
PC.ae.C40.1
0,8
0,6
0,4
0,2
0
1
2
3
4

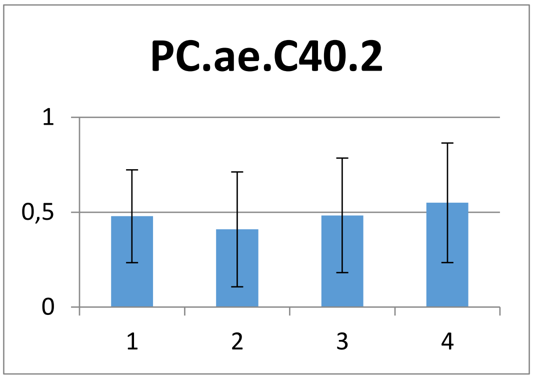
PC.ae.C40.2
1
0,5
0
1
2
3
4

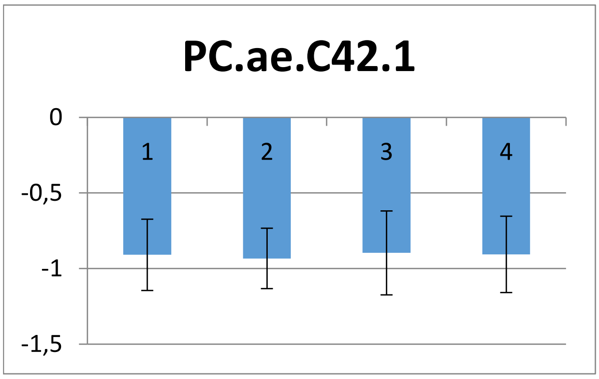
PC.ae.C42.1
0
-0,5
-1
-1,5
1
2
3
4

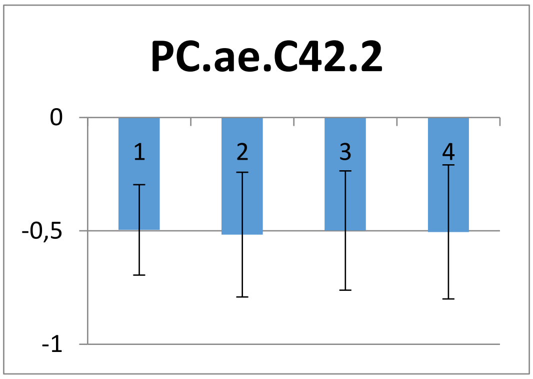
PC.ae.C42.2
0
-0,5
-1
1
2
3
4

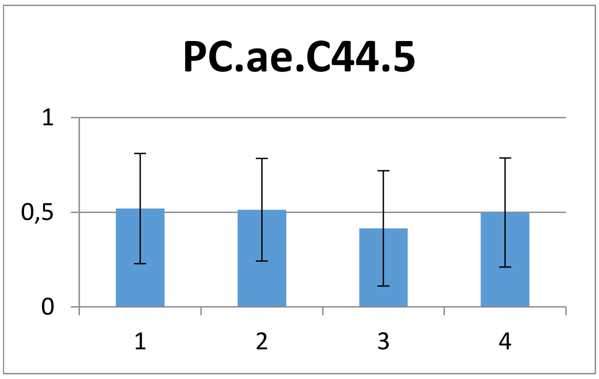
PC.ae.C44.5
1
0,5
0
1
2
3
4

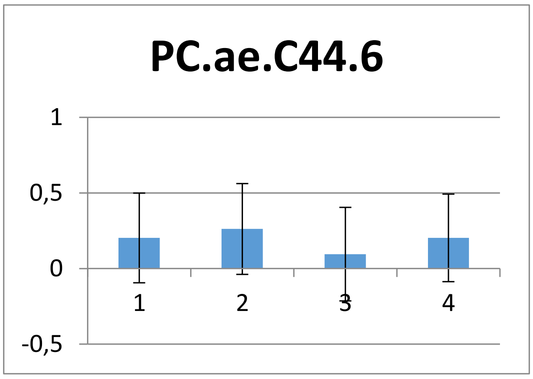
PC.ae.C44.6
1
0,5
0
-0,5
1
2
3
4

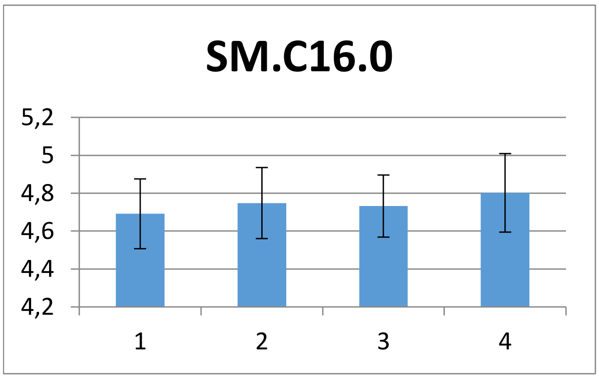
SM.C16.0
5,2
5
4,8
4,6
4,4
4,2
1
2
3
4

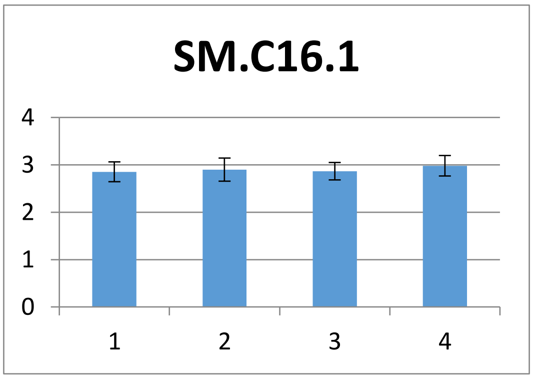
SM.C16.1
4
3
2
1
0
1
2
3
4

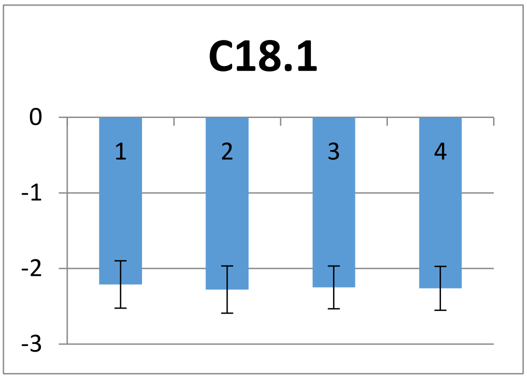
C18.1
0
-1
-2
-3
1
2
3
4

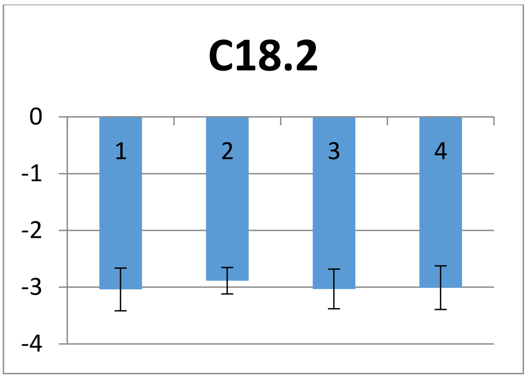
C18.2
0
-1
-2
-3
-4
1
2
3
4

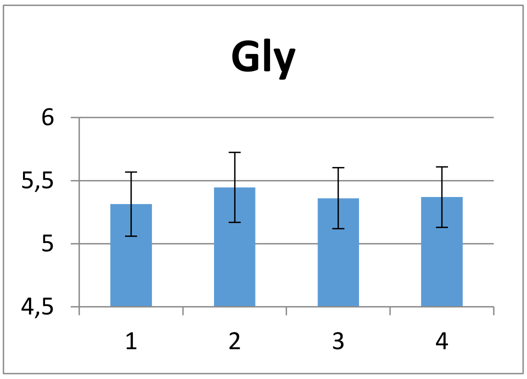
Gly
6
5,5
5
4,5
1
2
3
4

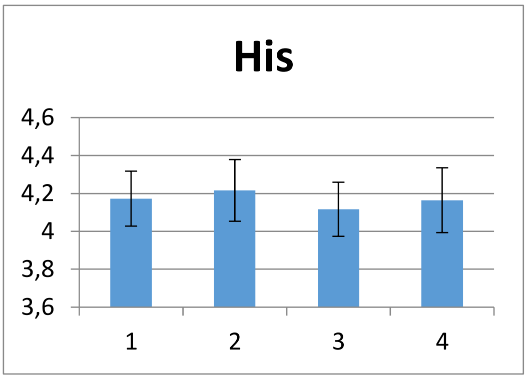
His
4,6
4,4
4,2
4
3,8
3,6
1
2
3
4

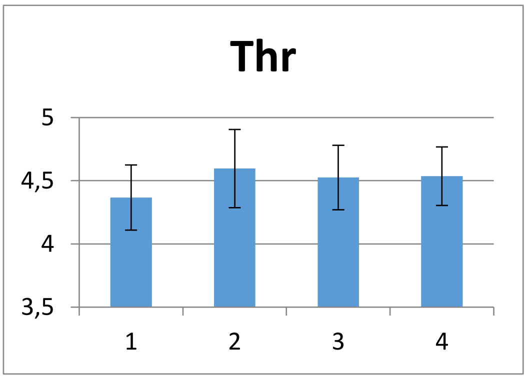
Thr
5
4,5
4
3,5
1
2
3
4

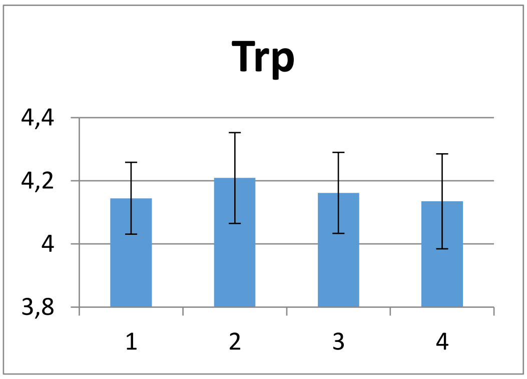
Trp
4,4
4,2
4
3,8
1
2
3
4

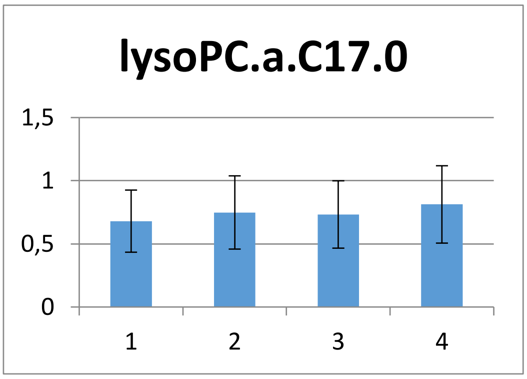
lysoPC.a.C17.0
1,5
1
0,5
0
1
2
3
4

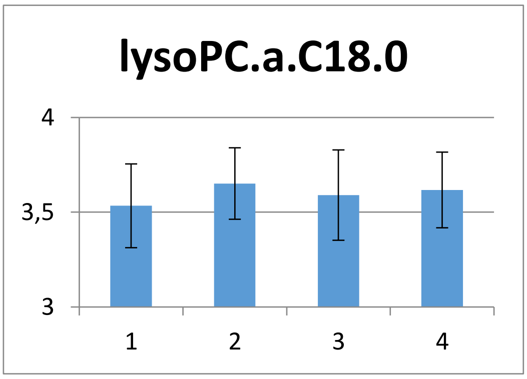
lysoPC.a.C18.0
4
3,5
3
1
2
3
4

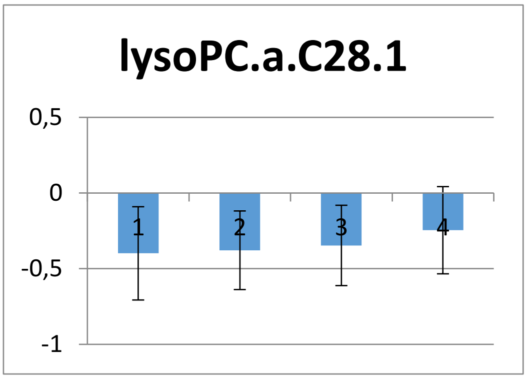
lysoPC.a.C28.1
0,5
0
-0,5
-1
1
2
3
4

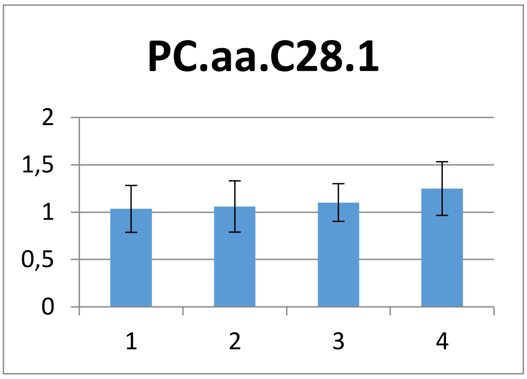
PC.aa.C28.1
2
1,5
1
0,5
0
1
2
3
4

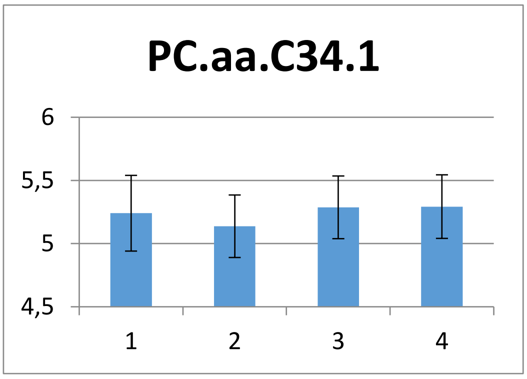
PC.aa.C34.1
6
5,5
5
4,5
1
2
3
4

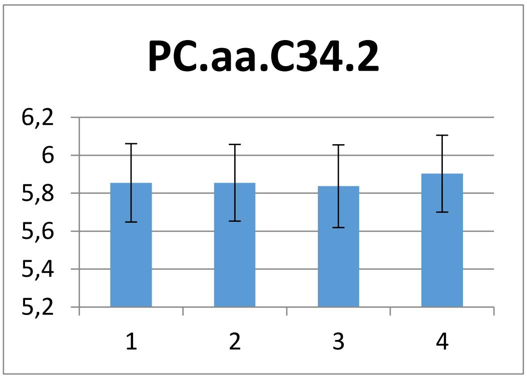
PC.aa.C34.2
6,2
6
5,8
5,6
5,4
5,2
1
2
3
4

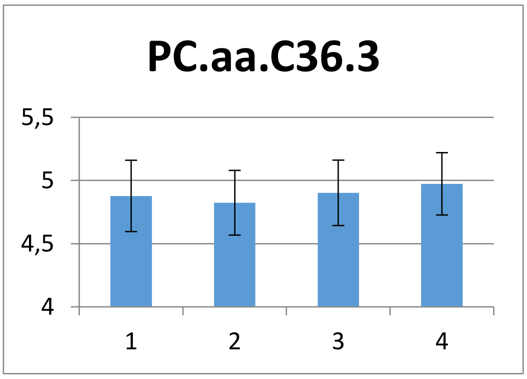
PC.aa.C36.3
5,5
5
4,5
4
1
2
3
4

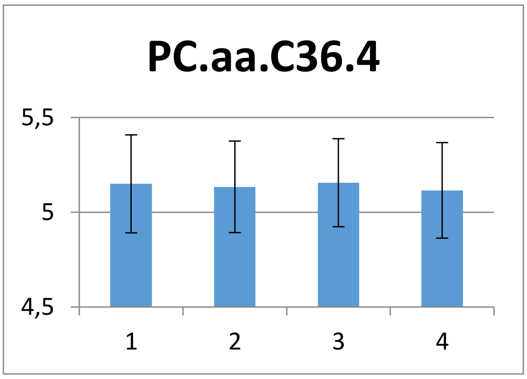
PC.aa.C36.4
5,5
5
4,5
1
2
3
4

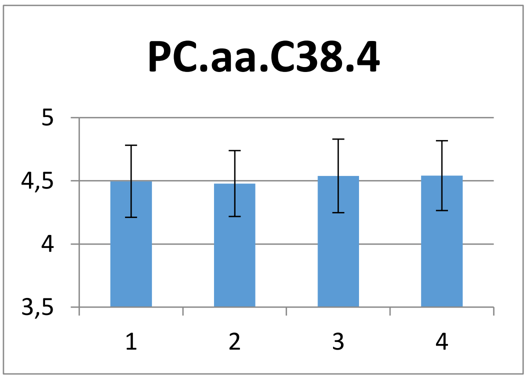
PC.aa.C38.4
5
4,5
4
3,5
1
2
3
4

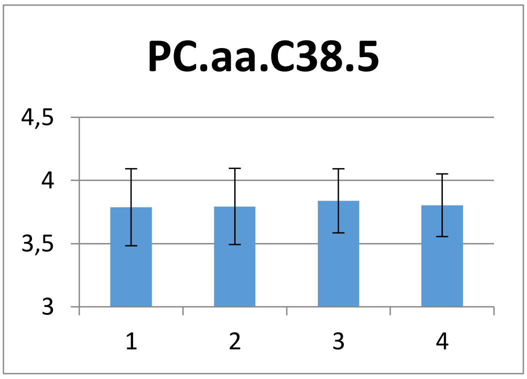
PC.aa.C38.5
4,5
4
3,5
3
1
2
3
4

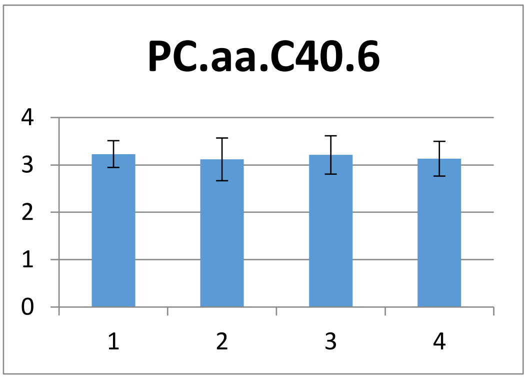
PC.aa.C40.6
4
3
2
1
0
1
2
3
4

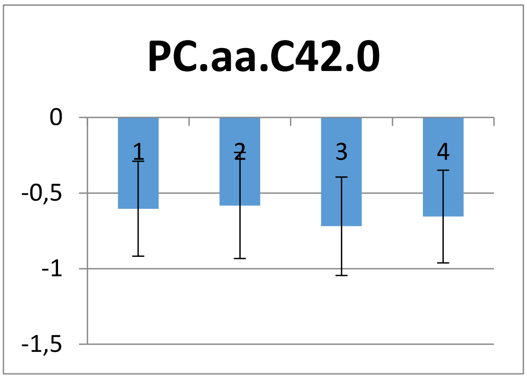
PC.aa.C42.0
0
-0,5
-1
-1,5
1
2
3
4

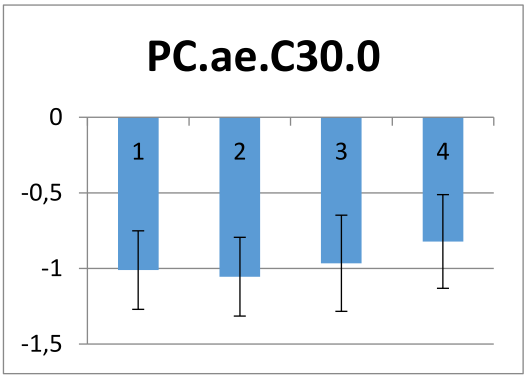
PC.ae.C30.0
0
-0,5
-1
-1,5
1
2
3
4

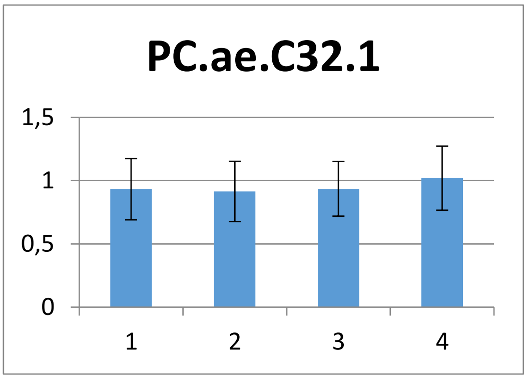
PC.ae.C32.1
1,5
1
0,5
0
1
2
3
4

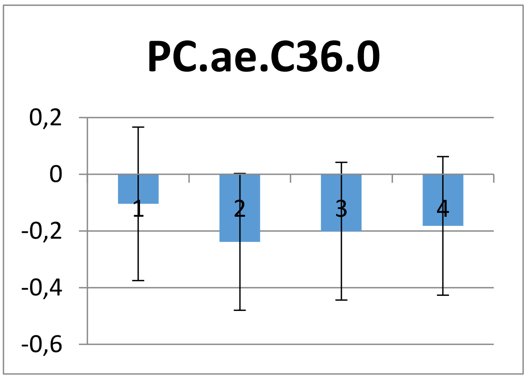
PC.ae.C36.0
0,2
0
-0,2
-0,4
-0,6
1
2
3
4

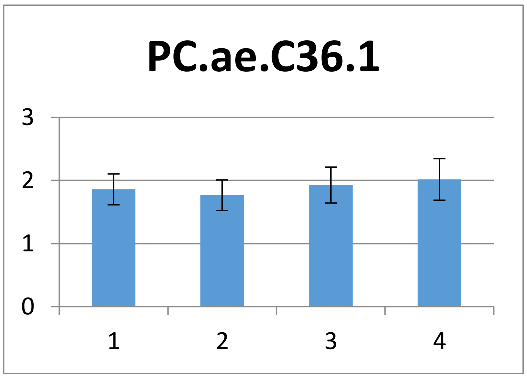
PC.ae.C36.1
3
2
1
0
1
2
3
4

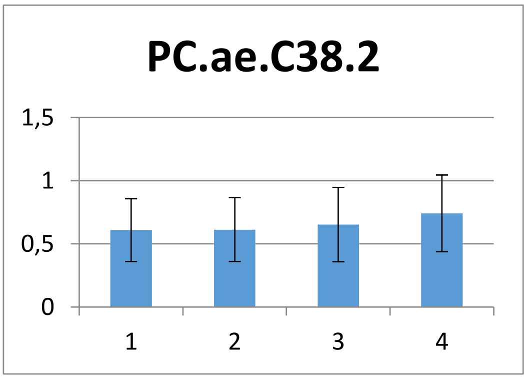
PC.ae.C38.2
1,5
1
0,5
0
1
2
3
4

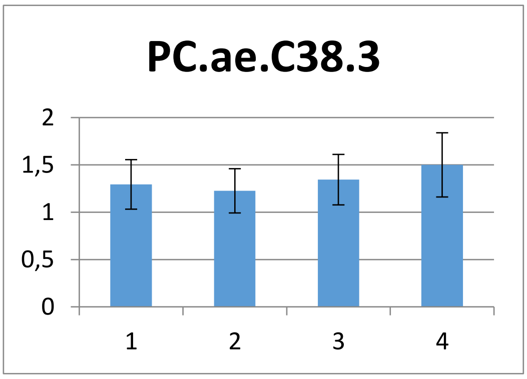
PC.ae.C38.3
2
1,5
1
0,5
0
1
2
3
4

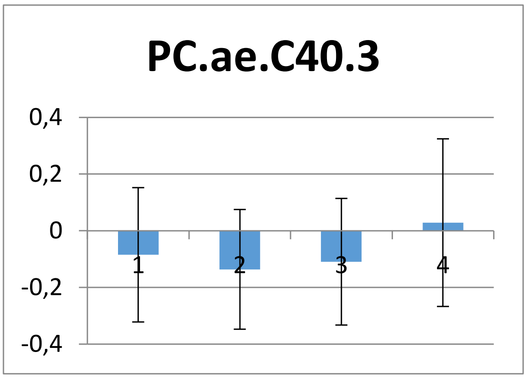
PC.ae.C40.3
0,4
0,2
0
-0,2
-0,4
1
2
3
4

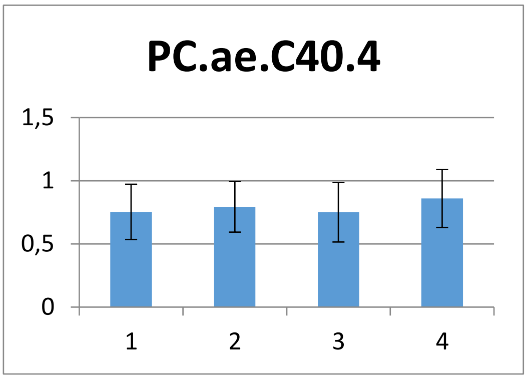
PC.ae.C40.4
1,5
1
0,5
0
1
2
3
4

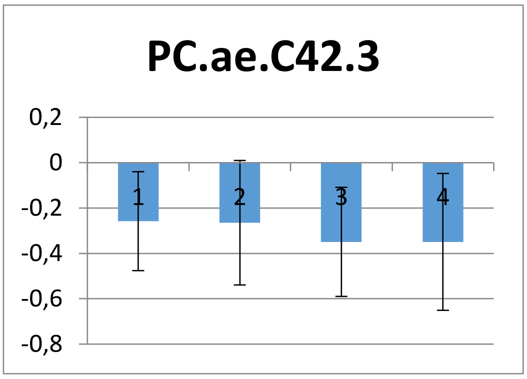
PC.ae.C42.3
0,2
0
-0,2
-0,4
-0,6
-0,8
1
2
3
4

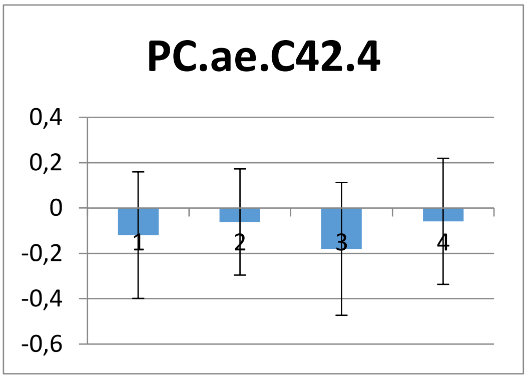
PC.ae.C42.4
0,4
0,2
0
-0,2
-0,4
-0,6
1
2
3
4

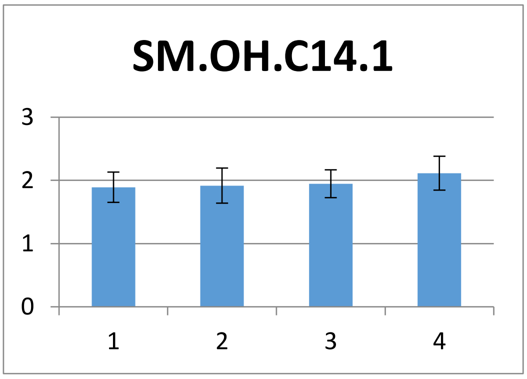
SM.OH.C14.1
3
2
1
0
1
2
3
4

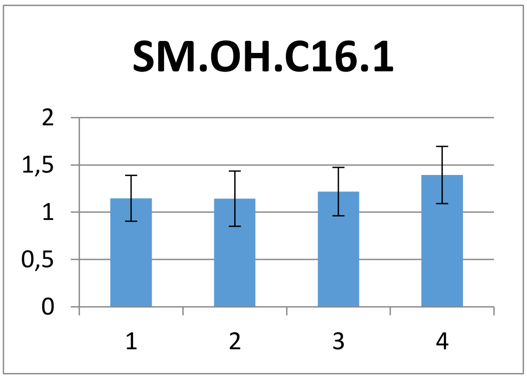
SM.OH.C16.1
2
1,5
1
0,5
0
1
2
3
4

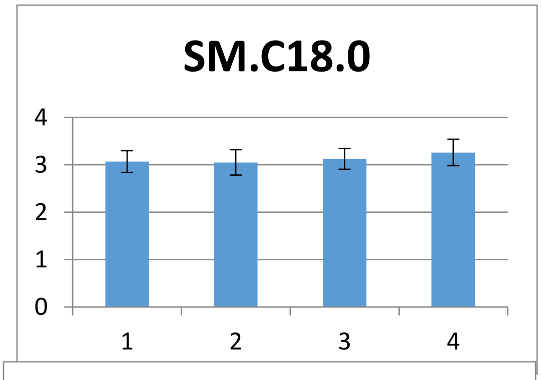
SM.C18.0
4
3
2
1
0
1
2
3
4

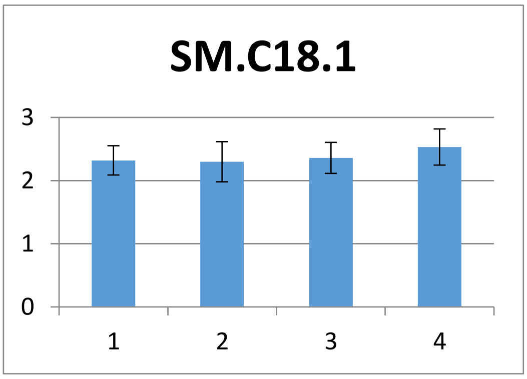
SM.C18.1
3
2
1
0
1
2
3
4

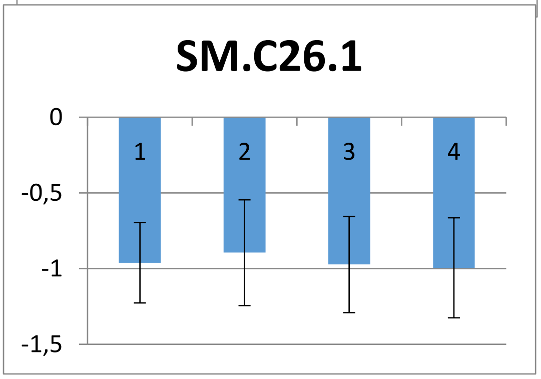
SM.C26.1
0
-0,5
-1
-1,5
1
2
3
4

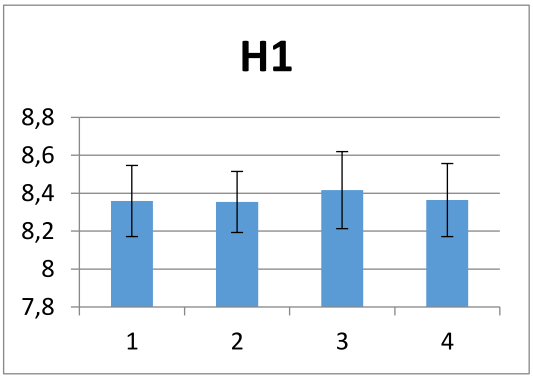
H1
8,8
8,6
8,4
8,2
8
7,8
1
2
3
4

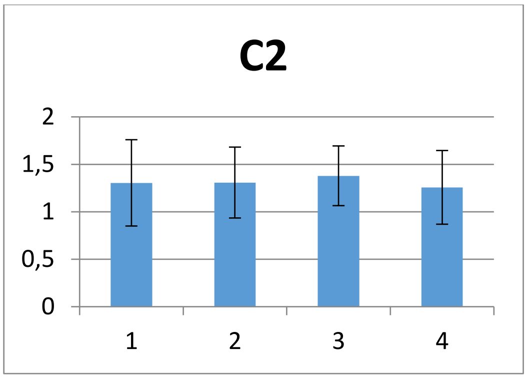
C2
2
1,5
1
0,5
0
1
2
3
4

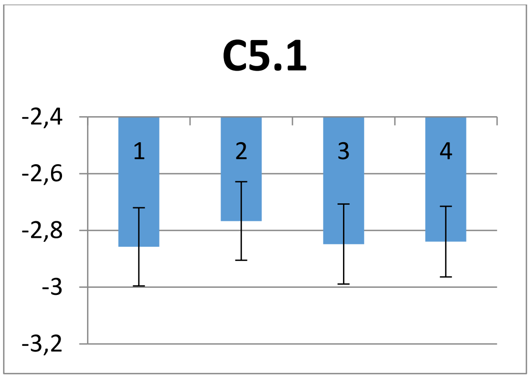
C5.1
-2,4
-2,6
-2,8
-3
-3,2
1
2
3
4

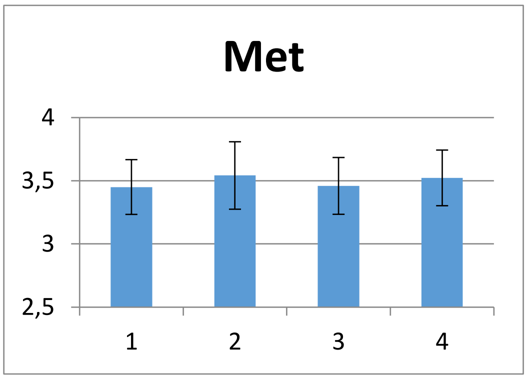
Met
4
3,5
3
2,5
1
2
3
4

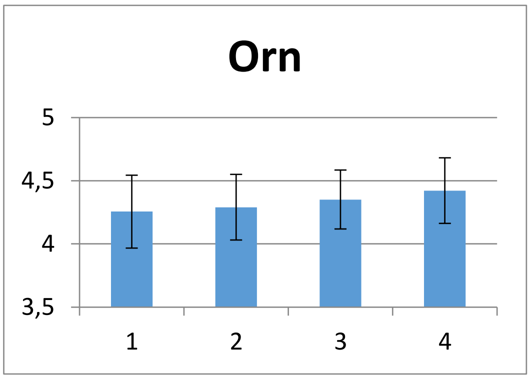
Orn
5
4,5
4
3,5
1
2
3
4

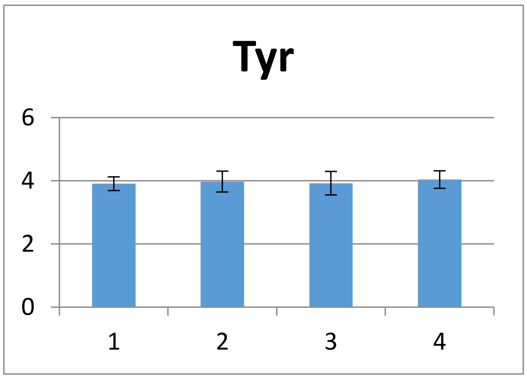
Tyr
6
4
2
0
1
2
3
4

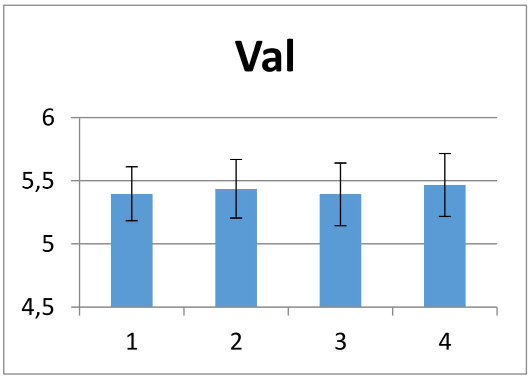
Val
6
5,5
5
4,5
1
2
3
4

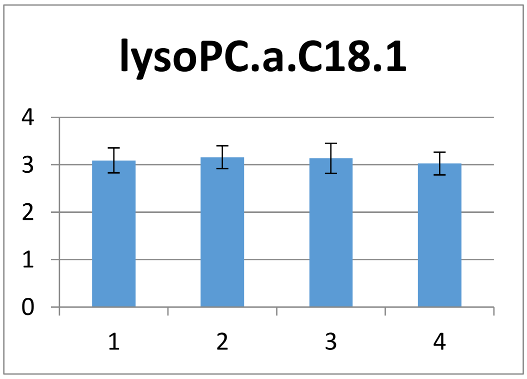
lysoPC.a.C18.1
4
3
2
1
0
1
2
3
4

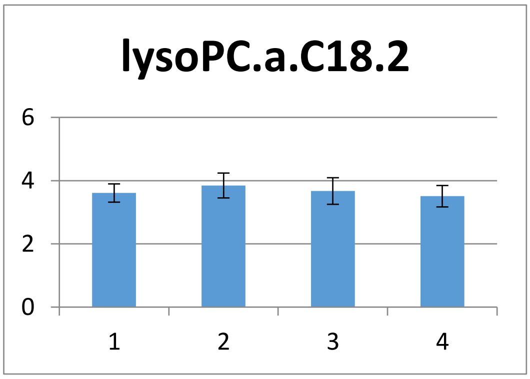
lysoPC.a.C18.2
6
4
2
0
1
2
3
4

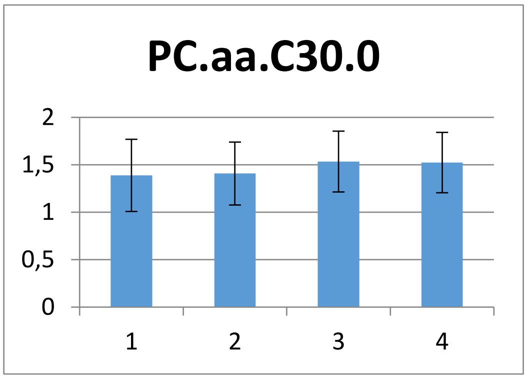
PC.aa.C30.0
2
1,5
1
0,5
0
1
2
3
4

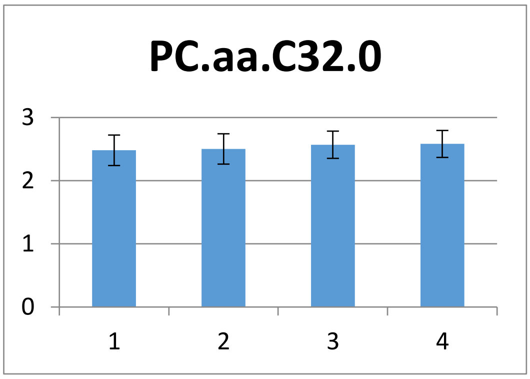
PC.aa.C32.0
3
2
1
0
1
2
3
4

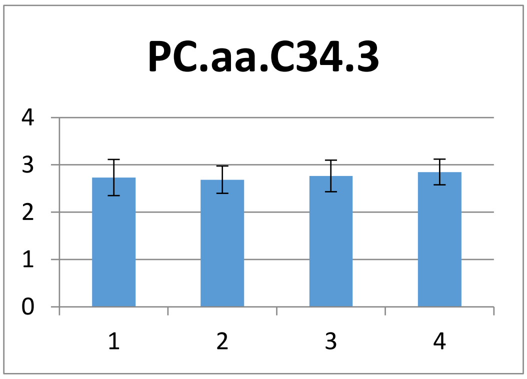
PC.aa.C34.3
4
3
2
1
0
1
2
3
4

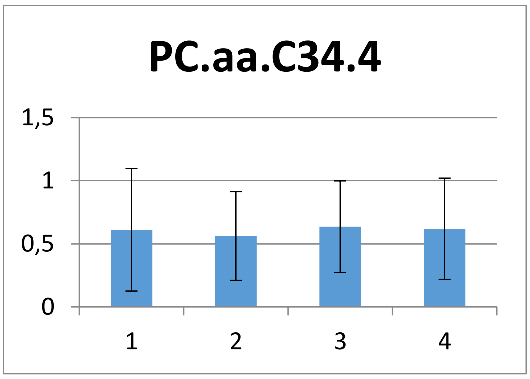
PC.aa.C34.4
1,5
1
0,5
0
1
2
3
4

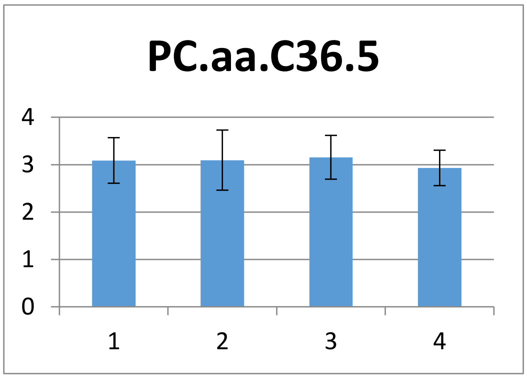
PC.aa.C36.5
4
3
2
1
0
1
2
3
4

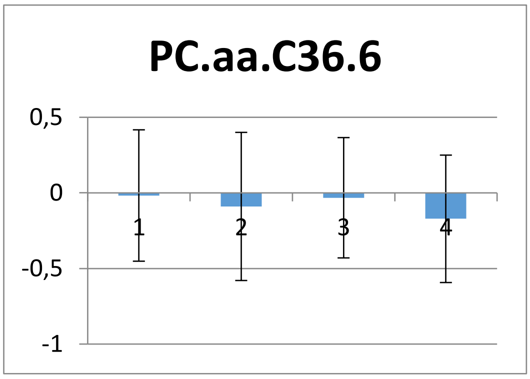
PC.aa.C36.6
0,5
0
-0,5
-1
1
2
3
4

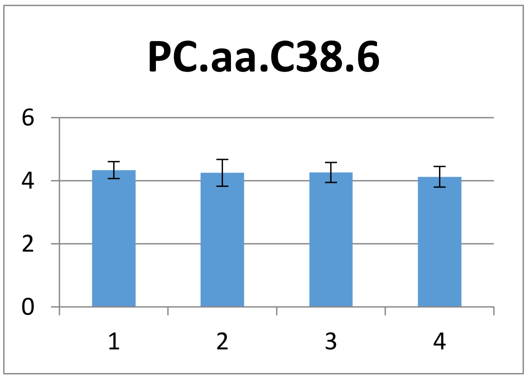
PC.aa.C38.6
6
4
2
0
1
2
3
4

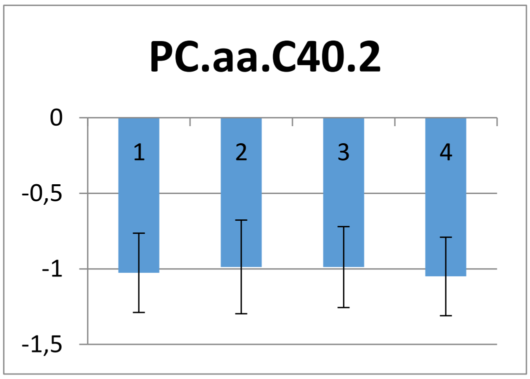
PC.aa.C40.2
0
-0,5
-1
-1,5
1
2
3
4

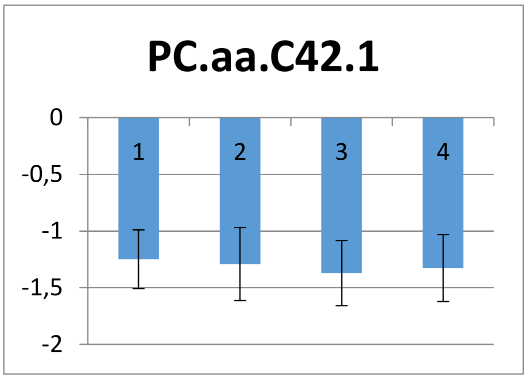
PC.aa.C42.1
0
-0,5
-1
-1,5
-2
1
2
3
4

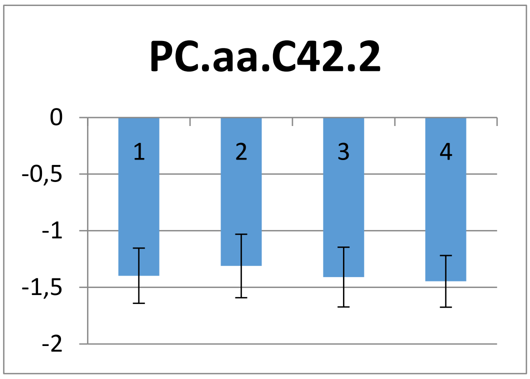
PC.aa.C42.2
0
-0,5
-1
-1,5
-2
1
2
3
4

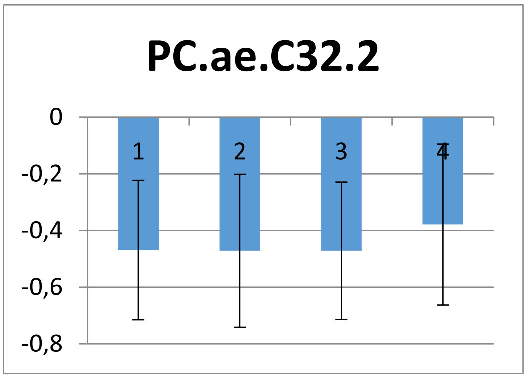
PC.ae.C32.2
0
-0,2
-0,4
-0,6
-0,8
1
2
3
4

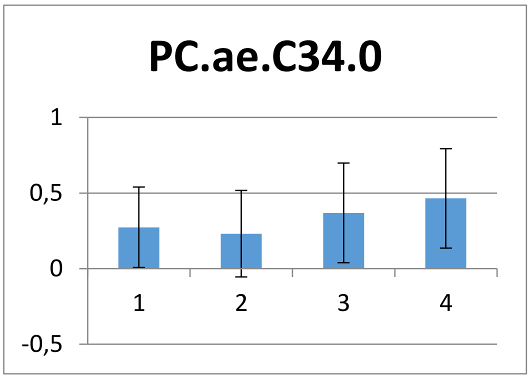
PC.ae.C34.0
1
0,5
0
-0,5
1
2
3
4

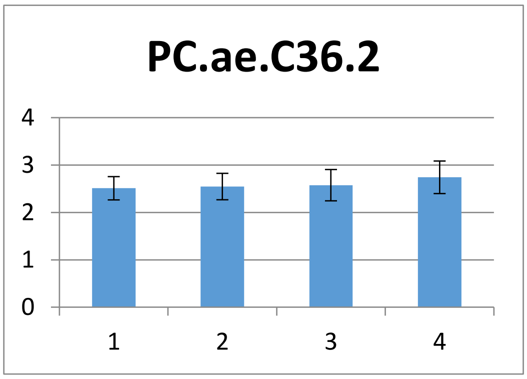
PC.ae.C36.2
4
3
2
1
0
1
2
3
4

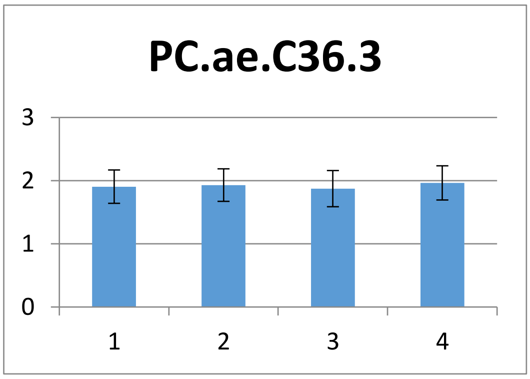
PC.ae.C36.3
3
2
1
0
1
2
3
4

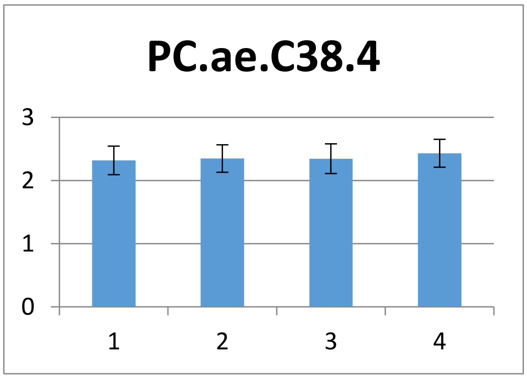
PC.ae.C38.4
3
2
1
0
1
2
3
4

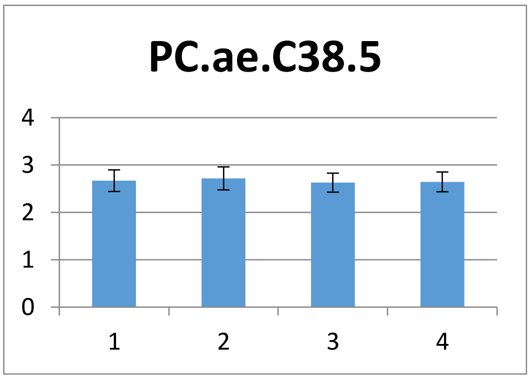
PC.ae.C38.5
4
3
2
1
0
1
2
3
4

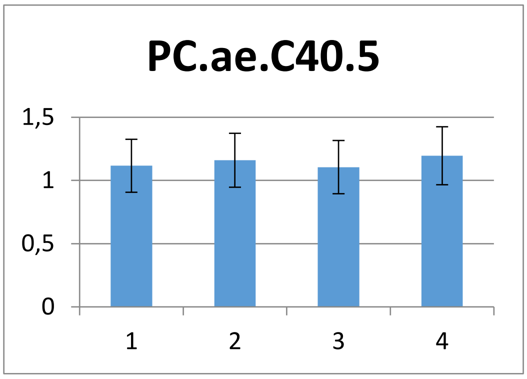
PC.ae.C40.5
1,5
1
0,5
0
1
2
3
4

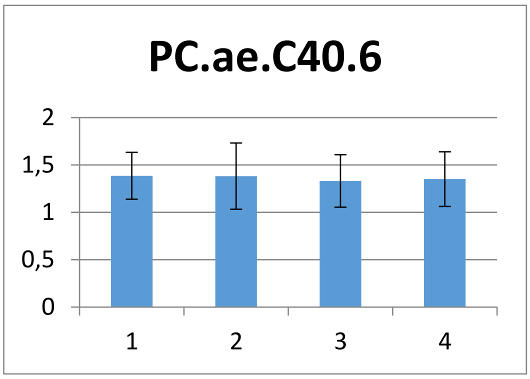
PC.ae.C40.6
2
1,5
1
0,5
0
1
2
3
4

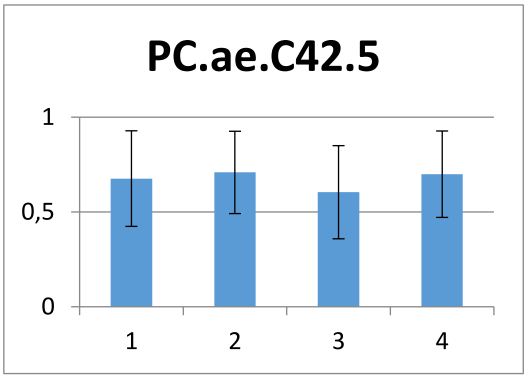
PC.ae.C42.5
1
0,5
0
1
2
3
4

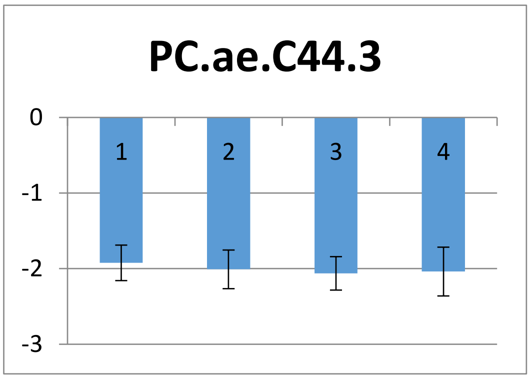
PC.ae.C44.3
0
-1
-2
-3
1
2
3
4

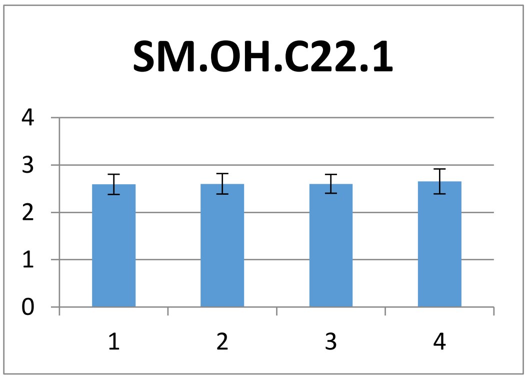
SM.OH.C22.1
4
3
2
1
0
1
2
3
4

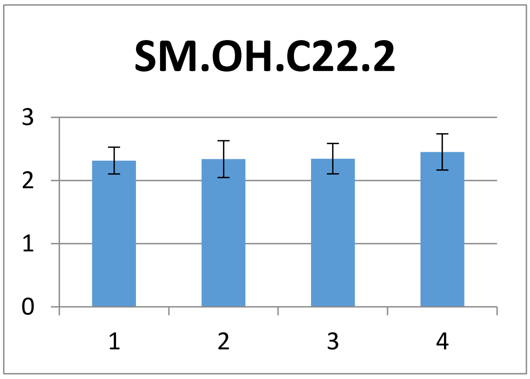
SM.OH.C22.2
3
2
1
0
1
2
3
4

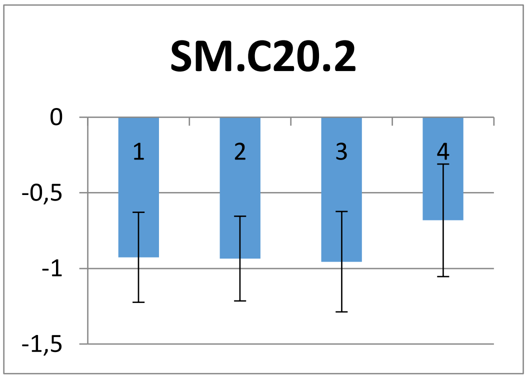
SM.C20.2
0
-0,5
-1
-1,5
1
2
3
4

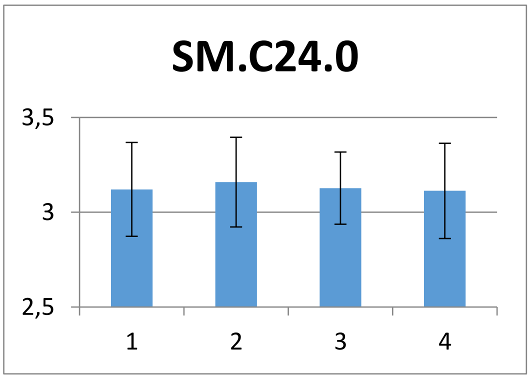
SM.C24.0
3,5
3
2,5
1
2
3
4

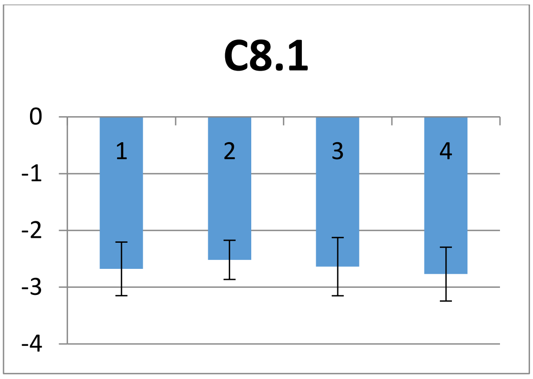
C8.1
0
-1
-2
-3
-4
1
2
3
4

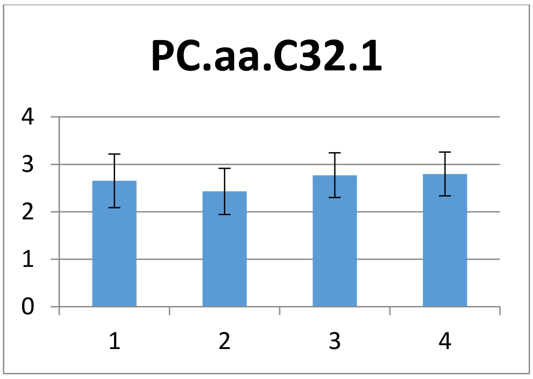
PC.aa.C32.1
4
3
2
1
0
1
2
3
4

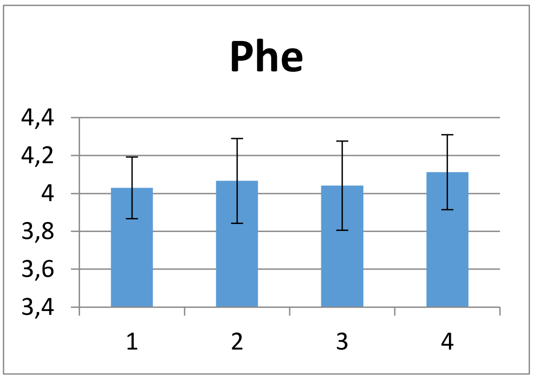
Phe
4,4
4,2
4
3,8
3,6
3,4
1
2
3
4

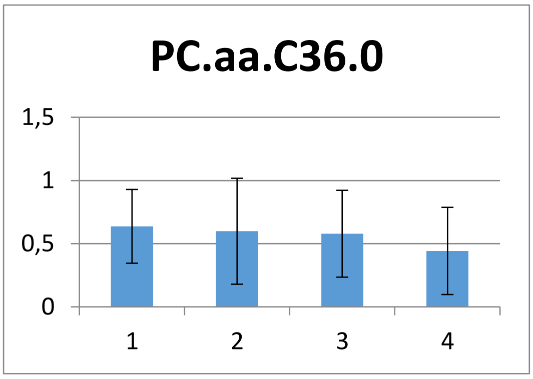
PC.aa.C36.0
1,5
1
0,5
0
1
2
3
4

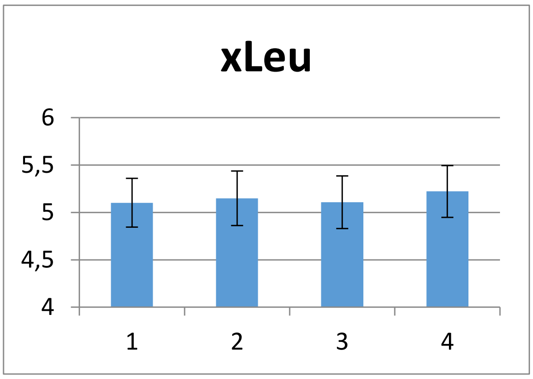
xLeu
6
5,5
5
4,5
4
1
2
3
4

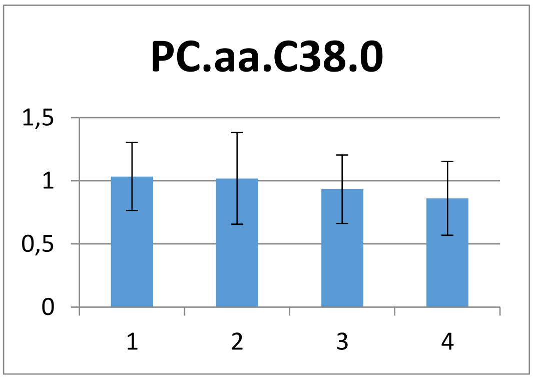
PC.aa.C38.0
1,5
1
0,5
0
1
2
3
4

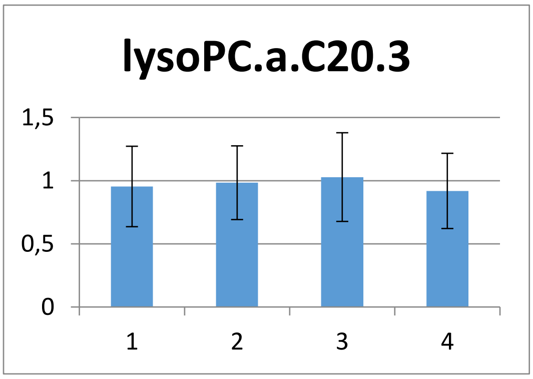
lysoPC.a.C20.3
1,5
1
0,5
0
1
2
3
4

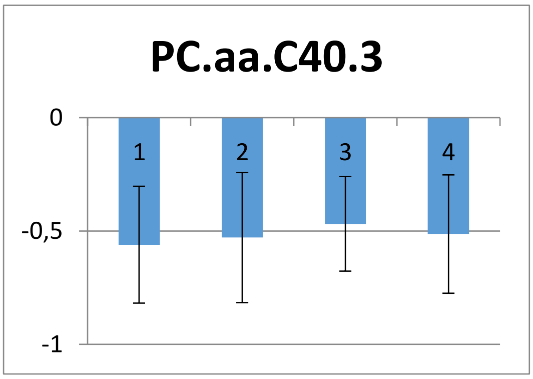
PC.aa.C40.3
0
-0,5
-1
1
2
3
4

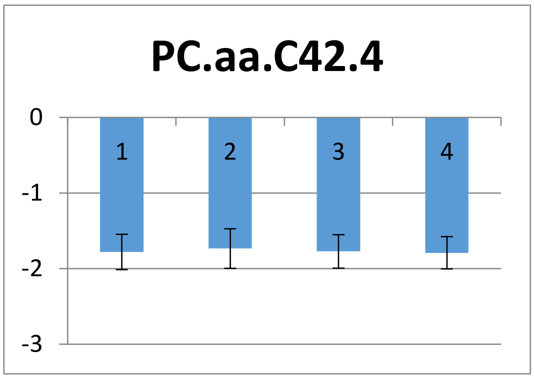
PC.aa.C42.4
0
-1
-2
-3
1
2
3
4

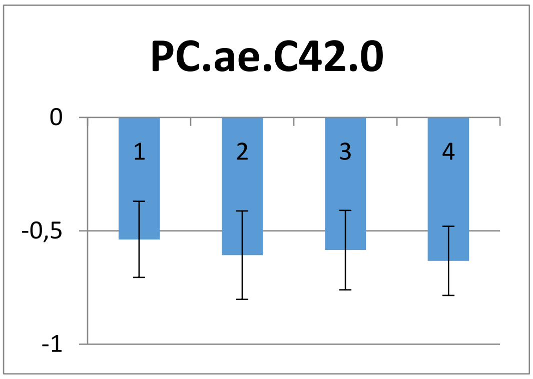
PC.ae.C42.0
0
-0,5
-1
1
2
3
4

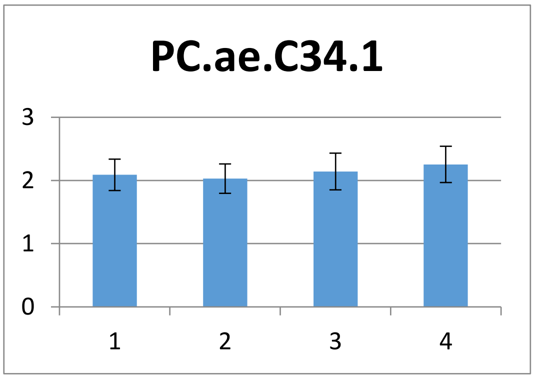
PC.ae.C34.1
3
2
1
0
1
2
3
4

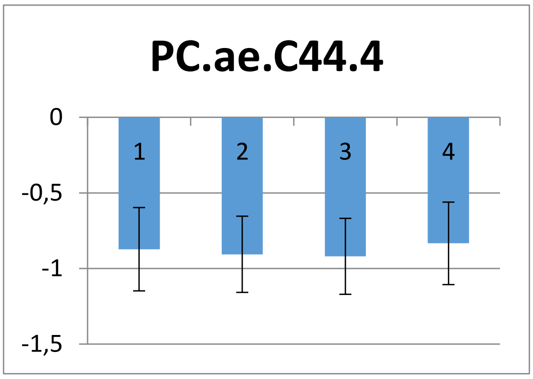
PC.ae.C44.4
0
-0,5
-1
-1,5
1
2
3
4

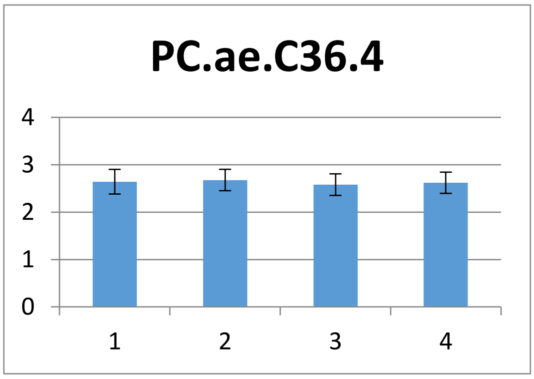
PC.ae.C36.4
4
3
2
1
0
1
2
3
4

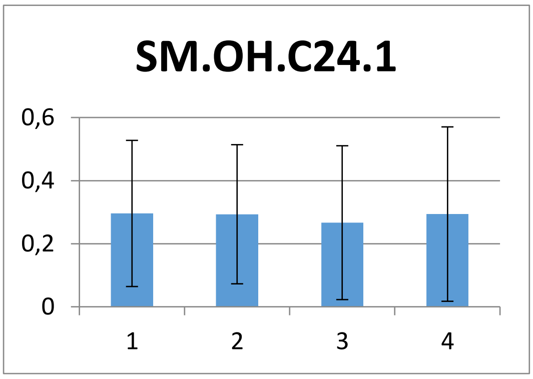
SM.OH.C24.1
0,6
0,4
0,2
0
1
2
3
4

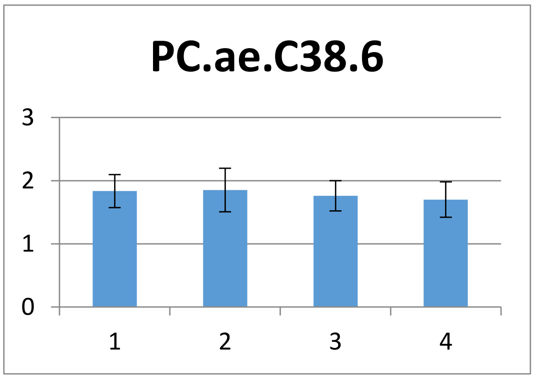
PC.ae.C38.6
3
2
1
0
1
2
3
4

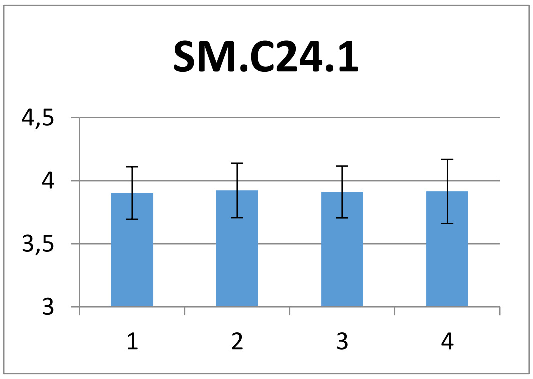
SM.C24.1
4,5
4
3,5
3
1
2
3
4
