# Supplementary material for: Integration of targeted metabolomics and transcriptomics identifies deregulation of phosphatidylcholine metabolism in Huntington’s disease peripheral blood samples
Source: Metabolomics. 2016 Jul 27;12:137. doi: 10.1007/s11306-016-1084-8 (PMC4963448; doi:10.1007/s11306-016-1084-8)
Supplement: Supplementary file 5 — Supplementary material 5 (DOCX 18 kb) [file 11306_2016_1084_MOESM5_ESM.docx]

**Supplementary File 5** Top 10 metabolite pair values from the association analysis of metabolite pair ratios using a 2 group design matrix (controls vs all HDs) and a 4 group linear modelling design matrix (controls and 3 HD disease stages). The metabolites pairs have been sorted according to decreasing p-gain values, resulting from the p-values of the metabolite pairs relative to the smaller of the individual metabolite p-values. Concentration changes were obtained from the fitted data of the metabolite pairs ratios using the full linear statistical model (see methods) and disease state and stage respectively as the main covariate.

| Metabolite pair IDs | HD Mutation Carriers vs Controls Analysis | P-gain | Concentration Change |
| --- | --- | --- | --- |
| Ser / PC ae C36:0 | Serine / Phosphatidylcholine acyl alkyl | 4.38E+03 | Higher in HD |
| PC.ae.C36:0 / PC.aa.C32:0 | Phosphatidylcholine acyl alkyl /diacyl | 2.06E+03 | Lower in HD |
| PC.ae.C36:0 / SM.C16:0 | Phosphatidylcholine acyl alkyl / Sphingomyelin | 8.62E+02 | Lower in HD |
| PC.ae.C36:0 / Thr | Phosphatidylcholine acyl alkyl / Threonine | 5.72E+02 | Lower in HD |
| PC.ae.C44.3 / Thr | Phosphatidylcholine acyl alkyl / Threonine | 4.45E+02 | Lower in HD |
| Arg / C0 | Arginine / Carnitine | 4.05E+02 | Higher in HD |
| PC.ae.C40.4:PC.ae.C44.3 | Phosphatidylcholine acyl alkyl / acyl alkyl | 1.96E+02 | Higher in HD |
| PC.ae.C42.0:Thr | Phosphatidylcholine acyl alkyl / Threonine | 1.83E+02 | Lower in HD |
| PC.ae.C44.3:Ser | Phosphatidylcholine acyl alkyl / Serine | 1.80E+02 | Lower in HD |
| PC.aa.C42.1:PC.ae.C44.6 | Phosphatidylcholine diacyl / acyl alkyl | 1.66E+02 | Lower in HD |
| Metabolite pair IDs | Disease Progression (4) Group Analysis | P-gain | Concentration Change |
| PC.ae.C36:0 / PC.ae.C34:1 | Phosphatidylcholine acyl alkyl /acyl alkyl | 1.16E+07 | Lower in HD |
| PC.ae.C36:1 / PC.ae.C36:0 | Phosphatidylcholine acyl alkyl /acyl alkyl | 7.26E+04 | Higher in HD |
| PC.ae.C40:6 / SM.OH.C16:1 | Phosphatidylcholine acyl alkyl / OH Sphingomyelin | 2.53E+04 | Lower in HD |
| PC.ae.C40:3 / PC.ae.C42:3 | Phosphatidylcholine acyl alkyl /acyl alkyl | 2.02E+04 | Higher in HD |
| PC.ae.C44:3 / PC.ae.C40:3 | Phosphatidylcholine acyl alkyl /acyl alkyl | 1.95E+04 | Lower in HD |
| PC.ae.C36:0 / PC.ae.C38:3 | Phosphatidylcholine acyl alkyl /acyl alkyl | 1.73E+04 | Lower in HD |
| SM.OH.C16:1 / PC.aa.C38:0 | OH Sphingomyelin / Phosphatidylcholine diacyl | 1.19E+04 | Higher in HD |
| PC.ae.C30:0 / PC.ae.C36:0 | Phosphatidylcholine acyl alkyl / acyl alkyl | 1.05E+04 | Higher in HD |
| PC.ae.C42:3 / PC.ae.C40:4 | Phosphatidylcholine acyl alkyl / acyl alkyl | 1.03E+04 | Lower in HD |
| PC.ae.C42:4 / PC.ae.C42:3 | Phosphatidylcholine acyl alkyl / acyl alkyl | 1.02E+04 | Higher in HD**^1^** |

**^1^** Median concentration lower in earlier symptomatics (group 3) and relative to pre-symptomatics (group2).
