# Supplementary material for: Integration of targeted metabolomics and transcriptomics identifies deregulation of phosphatidylcholine metabolism in Huntington’s disease peripheral blood samples
Source: Metabolomics. 2016 Jul 27;12:137. doi: 10.1007/s11306-016-1084-8 (PMC4963448; doi:10.1007/s11306-016-1084-8)
Supplement: Supplementary file 6 — Supplementary material 6 (DOCX 13 kb) [file 11306_2016_1084_MOESM6_ESM.docx]

| **Pathway ID** | **Pathway name** | **Pathway size** | **Hits** |
| --- | --- | --- | --- |
| WP2740 | Glycerophospholipid biosynthesis | 101 | *PLB1* - *PISD* – Serine - 1,2-diacyl-sn-glycero-3-phosphocholine (1+) |
| WP1880 | Phase II conjugation | 139 | *MTRR* - Serine - Threonine |
| WP1533 | Vitamin B12 Metabolism | 112 | MTRR - Serine |
| WP2650 | Arachidonic acid metabolism | 102 | ALOX5 - 1,2-diacyl-sn-glycero-3-phosphocholine (1+) |
| WP1857 | Metabolism of water-soluble vitamins and cofactors | 168 | MTRR - Serine |
| WP2693 | Metabolism of amino acids and derivatives | 358 | MTRR - Serine |
| WP176 | Folate metabolism | 132 | MTRR - Serine |
| WP2719 | Fc gamma receptor dependent phagocytosis | 79 | MYO10 - 1,2-diacyl-sn-glycero-3-phosphocholine (1+) |
| WP15 | Selenium Micronutrient Network | 188 | ALOX5 - Serine |
