# Supplementary material for: Integration of targeted metabolomics and transcriptomics identifies deregulation of phosphatidylcholine metabolism in Huntington’s disease peripheral blood samples
Source: Metabolomics. 2016 Jul 27;12:137. doi: 10.1007/s11306-016-1084-8 (PMC4963448; doi:10.1007/s11306-016-1084-8)
Supplement: Supplementary file 7 — Supplementary material 7 (DOCX 12 kb) [file 11306_2016_1084_MOESM7_ESM.docx]

**Supplementary File 1**

Cohort characteristics for the peripheral blood serum samples used for Biocrates targeted metabolomics profiling.

**Supplementary File 2**

Metabolite correlation heatmap. Color key represents correlation values (Red represents high correlation - Blue represents low correlation). Individual metabolite names are located at the bottom and at the right side of the heatmap.

**Supplementary File 3**

Data distributions of all 163 metabolites available in the Biocrates platform and as these were measured before quality control analysis. Each individual metabolite name is located at the bottom of the legend orthogonal boxes below the LOD and LLOQ values. (LOD = Limit of Detection / LLOQ = Lower limit of quantification).

**Supplementary File 4**

Observed concentration levels for the 114 metabolites that passed quality control prior to linear modelling fitting and for each of the 4 disease stage/progression groups used in the linear modelling analysis (see Materials and Methods) ranging from controls (1) to late symptomatics (4).

**Supplementary File 5**

Top 10 metabolite pair values from the association analysis of metabolite pair ratios using a 2 group design matrix (controls vs all HDs) and a 4 group linear modelling design matrix (controls and 3 HD disease stages). The metabolites pairs have been sorted according to decreasing p-gain values, resulting from the p-values of the metabolite pairs relative to the smaller of the individual metabolite p-values. Concentration changes were obtained from the fitted data of the metabolite pairs ratios using the full linear statistical model (see methods) and disease state and stage respectively as the main covariate.

**Supplementary File 6**

Metabolomics and transcriptomics overlapping pathways reported from the Wikipathways analysis.
